# A Tox21 Approach to Altered Epigenetic Landscapes: Assessing Epigenetic Toxicity Pathways Leading to Altered Gene Expression and Oncogenic Transformation In Vitro

**DOI:** 10.3390/ijms18061179

**Published:** 2017-06-01

**Authors:** Craig L. Parfett, Daniel Desaulniers

**Affiliations:** 1 Mechanistic Studies Division, Environmental Health Science and Research Bureau, HealthyEnvironments and Consumer Safety Branch, Health Canada, 50 Columbine Driveway, Tunney’s Pasture, Ottawa, ON K1A 0K9, Canada; 2 Hazard Identification Division, Environmental Health Science and Research Bureau, HealthyEnvironments and Consumer Safety Branch, Health Canada, 50 Columbine Driveway, Tunney’s Pasture, Ottawa, ON K1A 0K9, Canada; daniel.desaulniers@canada.ca

**Keywords:** carcinogenesis, carcinogenicity tests, carcinogens, chemical safety, chromatin, DNA methylation, environmental exposure, epigenesis, epigenomics, gene silencing, tumour suppressor, histones, neoplasms, nucleosomes, risk assessment

## Abstract

An emerging vision for toxicity testing in the 21st century foresees in vitro assays assuming the leading role in testing for chemical hazards, including testing for carcinogenicity. Toxicity will be determined by monitoring key steps in functionally validated molecular pathways, using tests designed to reveal chemically-induced perturbations that lead to adverse phenotypic endpoints in cultured human cells. Risk assessments would subsequently be derived from the causal in vitro endpoints and concentration vs. effect data extrapolated to human in vivo concentrations. Much direct experimental evidence now shows that disruption of epigenetic processes by chemicals is a carcinogenic mode of action that leads to altered gene functions playing causal roles in cancer initiation and progression. In assessing chemical safety, it would therefore be advantageous to consider an emerging class of carcinogens, the epigenotoxicants, with the ability to change chromatin and/or DNA marks by direct or indirect effects on the activities of enzymes (writers, erasers/editors, remodelers and readers) that convey the epigenetic information. Evidence is reviewed supporting a strategy for in vitro hazard identification of carcinogens that induce toxicity through disturbance of functional epigenetic pathways in human somatic cells, leading to inactivated tumour suppressor genes and carcinogenesis. In the context of human cell transformation models, these in vitro pathway measurements ensure high biological relevance to the apical endpoint of cancer. Four causal mechanisms participating in pathways to persistent epigenetic gene silencing were considered: covalent histone modification, nucleosome remodeling, non-coding RNA interaction and DNA methylation. Within these four interacting mechanisms, 25 epigenetic toxicity pathway components (SET1, MLL1, KDM5, G9A, SUV39H1, SETDB1, EZH2, JMJD3, CBX7, CBX8, BMI, SUZ12, HP1, MPP8, DNMT1, DNMT3A, DNMT3B, TET1, MeCP2, SETDB2, BAZ2A, UHRF1, CTCF, HOTAIR and ANRIL) were found to have experimental evidence showing that functional perturbations played “driver” roles in human cellular transformation. Measurement of epigenotoxicants presents challenges for short-term carcinogenicity testing, especially in the high-throughput modes emphasized in the Tox21 chemicals testing approach. There is need to develop and validate in vitro tests to detect both, locus-specific, and genome-wide, epigenetic alterations with causal links to oncogenic cellular phenotypes. Some recent examples of cell-based high throughput chemical screening assays are presented that have been applied or have shown potential for application to epigenetic endpoints.

## 1. Introduction

Somatic cells can develop long-lasting or multi-generational cellular phenotypes in response to a host of input signals originating from homeostatic physiological pathways, conditions of physiological stress, or abnormal cellular environments [1]. Induced phenotypic differentiation, either in normal or aberrant forms, is usually determined by changes to underlying programs of gene expression, shaped in part by epigenetic stabilization of gene transcriptional activities. The stabilized transcriptional state can persist even in the absence of the original stimulus because of the self-perpetuating properties of epigenetic systems [2]. Epigenetic controls are thought to respond to previous switches in gene activity through the actions of potentially self-maintaining, covalent DNA modifications and post-translational modifications of nuclear proteins that produce long- lasting, even mitotically heritable alterations in chromatin structure. The structural adaptations do not include changes in gene copy number or DNA sequence, as for example, in the generation polyploid hepatocytes in fully differentiated liver or in the early steps of B and T cell development that involve step-wise rearrangements of immunoglobulin and T cell receptor genes, respectively, or gene recombinations resulting from genotoxic DNA damage.

Epigenetic controls on gene expression mediated by cytosine methylation in DNA, covalent histone modifications, histone remodelling and long non-coding RNAs have often been studied for their independent or joint contributions to tumour initiation, promotion and progression. Epigenetic alterations have frequently been shown to accompany in vitro and/or in vivo experimental chemical exposure, but the series of essential, cause-and-effect molecular steps that lead from such exposure to the observed molecular-level alterations and beyond, to stable phenotypic changes, have seldom been explored in detail. At the outset of undertaking this review, there appeared to be an open question around whether or not there was sufficient understanding of epigenetic pathways controlling gene expression driving consequent cellular effects related to carcinogenesis and whether that knowledge might be usefully applied to furthering the development and implementation of chemical toxicity testing and risk assessment.

Currently, carcinogenic hazard characterization is dependent on two year rodent bioassays supported by adjunct in vivo or in vitro bioassays, such as the genotoxicity testing battery which assesses various damages and alterations to DNA sequence or copy number as processes causally linked to carcinogenicity. Additional assays may provide molecular biomarkers of effect related to increased cell proliferation, inhibition of programmed cell death, and receptor activation. The adjunct assays typically provide information on the mode or mechanism of action of the chemical agent in question, and help to establish whether the apical events in the animal model would be relevant to human cells and tissue responses. There is now much recognition that carcinogens may act through a large and diverse variety of mechanisms, including through induced epigenetic effects. Numerous investigations have shown that environmental influences (chemical, physical, biological) elicit epigenetic changes associated with adverse cell and tissue responses and a large number of these studies are related to cancer as an outcome [3,4,5,6,7]. Among these environmental influences are a number of substances that are of current concern to regulatory organizations responsible for chemical safety, due to wide-spread exposures of human populations. These substances span a wide spectrum of classifications, including both genotoxins and non-genotoxins, with endocrine disruptors (xenoestrogens, phthalates [8]), particulate matter (air pollution, nanoparticles [9]), and metals, being some prominent examples. Several recent literature reviews have examined how measures of epigenetic alterations have potential to contribute actionable information on modes and mechanisms of action as well as dose response analyses employed in human health risk assessment frameworks directed toward regulating environmental pollutants and consumer chemicals [6,10,11,12,13]. Epigenetic mechanisms are under consideration as crucial contributing information for an integrated approach to the testing and assessment of non-genotoxic carcinogens beginning to be developed internationally at the Organization of Economic Cooperation and Development (O.E.C.D.) [13].

In addition to further understanding mechanisms of action for carcinogens, there are unique advantages in considering epigenetic measurements [14,15]. Epigenetic endpoints offer multiple biomarkers to contribute to risk assessment frameworks, whether the oncogenic events originate from DNA reactive or non-reactive chemicals that may have genotoxic or non-genotoxic classifications. In clinical medical applications, it is recognized that alterations in DNA methylation are less sensitive to sample degradation than gene expression changes, which is a feature that facilitates robust and reliable non-invasive biomarker assays. Such changes also occur in higher frequencies than genetic mutations and at earlier stages of tumourigenesis, which strengthen their use as clinical biomarkers for diagnostic, prognostic, and therapeutic application. Similarly, for chemical risk assessment purposes, these features of marker stability and relatively high frequencies of occurrence, combine with growing evidence for causal participation in oncogenic processes to offer the promise that epigenetic alterations can become valuable molecular diagnostic and prognostic biomarkers of carcinogenic risk.

The present review focuses on what is known about epigenetic mechanisms of gene expression control that are causal contributors to the oncogenic transformation of human cells by environmental chemicals. The review highlights some notable experimental studies in human somatic cell cultures, combined with overviews from detailed reviews in order to summarize the basic molecular components forming in vitro epigenetic pathways leading to tumour suppressor gene silencing and to oncogenic cellular phenotypes. An emphasis has been put on knowledge obtained from human in vitro cellular systems, drawing on studies from over about the last 10 years that have included functional assessments of oncogenic phenotypic endpoints. Especially informative in this regard are studies with direct, experimental perturbations to epigenetic pathway components created by genetically engineered manipulations or by using chemical agents that are known activators or inhibitors of the pathway components. It is expected that when similar in direction and magnitude, perturbations caused by exposure to chemicals would function similarly to the engineered manipulations that created toxicity pathways leading to transformed cell variants. The review finally provides a summary of how the measurements and tools that have provided underlying mechanistic knowledge are beginning to be harnessed to provide high-throughput chemical screening assays based on key steps in the pathways.

Despite the increased insight that may become available by incorporating epigenetic information into carcinogenicity testing strategies, the costs incurred, in terms of financial resources, time, and issues regarding animal welfare, that are involved to produce traditional apical endpoint data combine to form a significant impediment to obtaining information for the large number of chemicals that await evaluation. These inherent difficulties accompanying animal testing have encouraged development of the Tox21 strategies for health risk identification and assessment that are summarized in the following sections.

### 1.1. Considerations in Applying the Tox21 Strategy for Human Health Risk Assessment to the Epigenetic Mode of Action and Mechanistic Pathways Leading to Carcinogenesis

The overarching narrative in the Tox21 vision for toxicity testing (TT21C) documents [16,17,18] and “next-generation” risk assessment framework [19] was an outline for a plan to develop and implement a reversal of traditional “top-down” toxicological testing approaches. Traditional testing has relied primarily on measures of apical phenotypic events in high-dose animal studies, supported by mechanistic studies, as mentioned above. The proposed new direction encompasses a more “bottom-up” approach to testing, starting with measures of perturbations to molecular interactions that are known to trigger toxicologically-relevant cell biological effects. Preferably, the molecular measurements would be conducted in cell culture systems of human origin. The re-directed in vitro approach takes advantage of the most recent scientific advances in quantitative high-throughput and high-content measurement methodologies to aid in describing molecular pathways leading from the initiating triggers through to cell-level toxicity endpoints [20,21]. Such “pathways of toxicity” (PoT) are thought to be derived from cellular response and signaling pathways that may result in adverse cellular effects when their responses become sufficiently perturbed. For example, exposures to chemicals may induce excessive activating or suppressive responses, or may continue over an extended duration. Adverse phenotypic endpoints mediated by epigenetic controls, as a mode of action, may be traced back through the key events of toxicity pathways to various start-points or molecular initiating events, including responses to cellular stresses. Several examples in the present review are linked to oxidative stress, DNA damage, or DNA replication stress caused by aberrant replication fork structures. Toxicity pathways may also initiate as receptor-mediated responses, including inappropriate chemical interactions of certain non-mutagenic carcinogens with the nuclear receptors of circulating hormones [e.g., xenoestrogens, 2,3,7,8-tetrachlorodibenzo-*p*-dioxin (TCDD)]. In addition, maladaptive responses in the homeostatic controls on cellular physiology may cause changes to critical metabolites involved in epigenetic functions. Important metabolic co-factors in epigenetic functions include: glutathione which is important for DNA and histone methylation reactions; ATP for histone phosphorylations; acetyl-coenzyme A for histone acetylations; flavin adenine dinucleotide (FAD) as a cofactor for the lysine-specific histone demethylase 1 (LSD1) histone demethylase; NAD+ as cofactor for the SIRTUIN family of histone deacetylases; α-ketoglutarate, as a co-factor for histone demethylases (HDMs) and for the Ten-eleven translocation (TET) protein dioxygenase family members that oxidize the methyl group of 5-methylcytosine and facilitate DNA demethylation [22,23,24]. Thus, under the Tox21 view, perturbations of the molecular pathways to the point that they extend beyond the ranges measured in model human cell cultures, or lead in new directions, to become “drivers” of cellular toxicity, are the mechanistic links between the chemical exposures at the cellular level and the toxicologically-relevant changes in cellular behaviors. The Pathway of Toxicity (PoT) concept places emphasis on the cellular context, but is linked to the broader Adverse Outcome Pathway (AOP) approach that has been under development within the chemical safety testing guideling programme of the O.E.C.D. AOPs are an organizing scheme for existing knowledge about toxicity mechanisms, beginning with molecular initiating events created by chemical stressors, leading to a series of key measurable events spanning many levels of biological organization from the molecular to subcellular, cellular, tissue, organ, organism and finally population-level effects [25]. The salient features of the PoT and AOP concepts have been compared and contrasted by Hartung [26].

For the purposes of chemical toxicity testing, a central feature of the Tox21 strategy requires that the dynamics of the measurable molecular steps in functionally validated toxicity pathways can be used as surrogate measures for the adverse cell-biological phenotypic endpoints that they control. Several important considerations need to be satisfactorily addressed before any presumptive toxicity pathway and short-term assays for its key steps may be employed effectively in making regulatory decisions regarding chemical safety [27]. These considerations will also be relevant to the molecular pathways emerging from investigations more specifically concerned with epigenetic control of gene expression.

Firstly, the choice of key molecular perturbations along the pathway requires solid, experimentally-confirmed insights into their causal linkages (essentiality in the AOP vocabulary), together with dose- and time-related contributions to adverse cellular outcomes. Secondly, such acquired knowledge, gained in uncovering the linkages between exposure and phenotypic response, should be applied to the development of efficient tests that permit medium- to high-throughput chemical screening. Suitable validations of key high throughput tests for quantitative accuracy and interlaboratory transferability, as well as specification of performance standards, would provide the confidence to undertake screenings and dose-response analysis of large numbers of chemicals for human health hazards. Finally, multi-modal high through-put test batteries would be chosen to enhance the sensitivity of screening, based upon a selection of the key (sufficient or causally required) events within the described toxicity pathways.

Following Tier 1 high-throughput screenings and considering the key assay outputs and jurisdictional regulatory requirements, priority assignments might be given to selected chemicals for confirmative, in-depth evaluation in more labour intensive tests (Tier 2 & Tier 3). For example in Tier 2 work, more complex in vitro tissue models could add information on intercellular effects contributing to toxicity, followed by Tier 3 limited animal experimentation to ascertain in vivo relevance [19].

An outline of a toxicity pathway-based approach to cancer-related human health risk assessment relying on in vitro data was recently presented for quercetin [28]. Adversity was defined as the “tipping point” leading from non-transcriptional to transcriptional responses of the p53 tumour suppressor protein which was interpreted to be a surrogate for risks from unrepaired DNA damage. In vitro dose-response information, combined with computational modelling of p53 pathway dynamics, were extrapolated to presumed human plasma and tissue concentrations. Likely human tissue concentration ranges were derived from in vivo exposure estimates and estimations of in vivo doses by pharmacokinetic modelling. These data were combined to predict human systemic exposure levels below which adverse pathway perturbations would not occur. It is expected that further refinements to characterizing hazards, based on dose-dependent perturbations to toxicity pathways, will provide a practical framework for making decisions concerning chemical safety.

With this provisional framework in view, it becomes important to assess whether our current understanding of toxin-induced epigenetic effects that drive cell transformation can provide a basis to develop mechanism-based toxicity testing suitable for regulatory decision-making according to the Tox21 paradigm.

### 1.2. TSG Silencing Models in Cell Transformation

**Figure 1:**
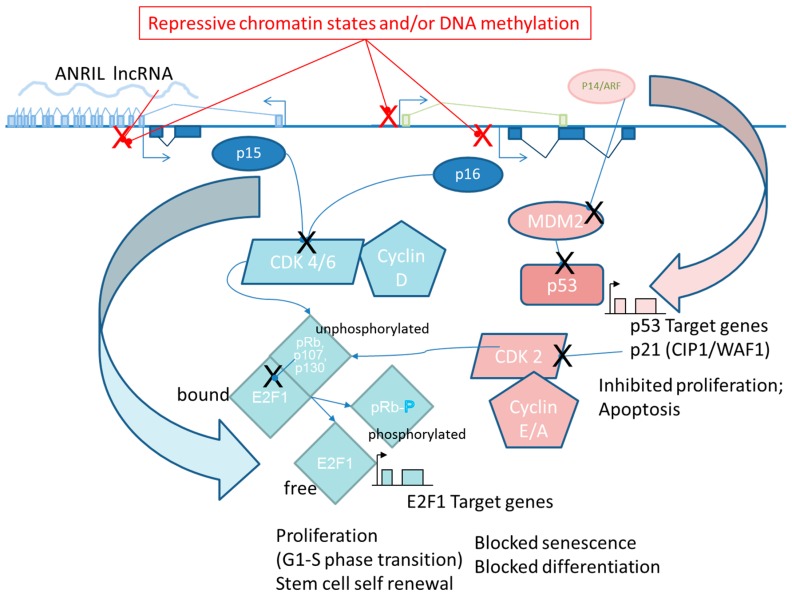
Schematic representation of the human 42 Kb *INK4-ARF* (*CDKN2A/B*) locus (not to scale) and its participation in two pathways controlling cell-cycling and differentiation. Two pathway branches leading to tumor suppression originate from the locus, influencing E2F1 transcription factor availability (large blue arrow) and the p53 protein stability (large red arrow). Suppressive chromatin states and/or DNA methylation (red Xs) can silence gene expression from the locus. Expression of p16 and p15 is controlled by a collection of activating and repressive transcription factors (not shown). When expressed, p16 and p15 bind to cyclin-dependent kinases (CDK4 and CDK6) which inhibits their phosphorylation of the retinoblastoma tumor suppressor protein (pRB) (small bule arrows). Hypophosphorylation of pRB and its homologues (p107, p130) due to lack of cyclin-dependent kinase activity allows them to sequester E2F1 transcription factor and prevent its transport to the nucleus, where it would otherwise activate a battery of genes involved in cell proliferation and stem cell renewal. Expression of p14/ARF inhibits the E3 ubiquitin ligase activity of MDM2 (mouse double minute 2) thereby stabilizing p53 transcription factor, resulting in up-regulated expression of its target genes promoting apoptosis and inhibiting proliferation. One p21 inhibitor of CDK2 (cyclin-dependent kinase 2) which targets pRB. One important cross connection between the two branches is indicated, operating through the cyclin-dependent kinase inhibitor, p21/CIP1/WAF1. Normal inhibitory interactions between components within the two pathways are indicated by black Xs. Not indicated are many other interconnections between the two signaling circuits that provide both complexity and redundancy to the pathways, a topic covered more thoroughly by references provided herein.

Epigenetic silencing of tumour suppressor genes (*TSG*) is considered to be a causative mechanism in human carcinogenesis and these events are often described as early molecular events in the progressive transformation of normal cells into highly malignant derivatives [29,30]. Tumour-suppressor proteins function in cell cycle checkpoints that delay the cell cycle in response to DNA damage or other physiological stresses. Many also function in DNA repair pathways, in apoptosis (programmed cell death) or suppressing tissue invasion and metastasis by blocking loss of contact inhibition, or loss of anchorage dependence thereby preventing tumour cell dispersal [31,32]. Moreover, epigenetic states of *TSG* can be experimentally altered in human cells cultured in vitro resulting in *TSG*s expression changes that can directly modulate in vitro transformed phenotypes (increased or decreased oncogenic behavior) through perturbations to many of the same pathways deregulated in tumour cells. Such engineered changes, anchored to phenotypic outcomes, are illustrated in many studies using various human cell models cited throughout this review. In light of the previously mentioned, precedent-setting use of a toxicity pathway based upon p53 tumour-suppressor functions as a basis for dose-response assessment, particular attention has been given in this review to epigenetic controls regulating (silencing) the expression of the archetypal INK4B-ARF-INK4A-pRB (inhibitor of cyclin-dependent kinase 4B; ADP-ribosylation factor; inhibitor of cyclin-dependent kinase 4A; retinoblastoma protein) tumour suppressor pathway as a mechanism in oncogenic transformation of human cells. This pathway is functionally impaired or inactivated by genotoxic or epigenetic mechanisms in almost all human tumours and its inactivation, in vitro, also leads to stable changes in cell growth/differentiation phenotypes. The phenotypic effects resulting from long-lasting changes in gene expression or loss-of-function among the pathway components have important effects on cell cycle inhibition, stress-induced apoptosis, stem cell proliferation and oncogene- or stress-induced cell senescence [33,34,35] (Figure 1). These changes impact the frequency and severity of oncogenic phenotypes that can be revealed in human cell cultures. Induced changes in epigenetic toxicity pathways controlling well-understood *TSG* functions should provide a means to gauge carcinogenic hazards due to an emerging mode-of-action that has now begun to be more widely assessed for its contributions to both oncogenesis in vivo and cell transformation in vitro.

### 1.3. A Molecular Interpretation of the Waddington Epigenetic Landscape Model for the Development of Cellular Identity and as the Basis for Insight into the Epigenetic-Mode-of-Action of Carcinogens

**Figure 2:**
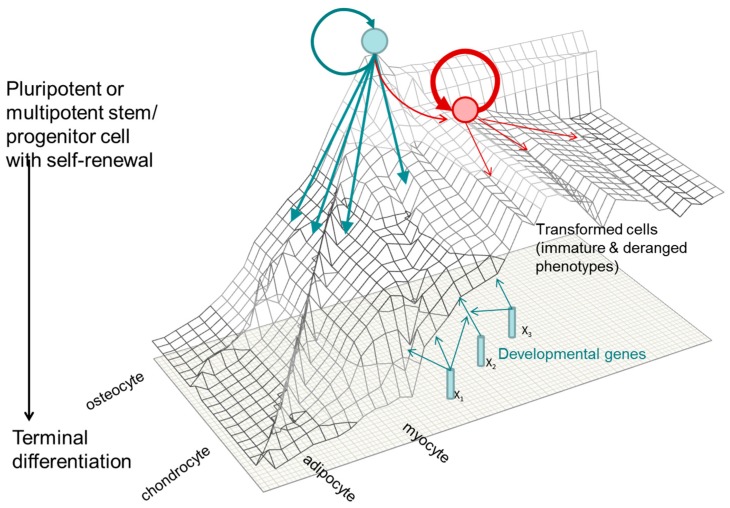
The epigenetic landscape metaphor for cell transformation. The model presents a progenitor/tissue stem cell with an early multipotent phenotype and self-renewal capacity (circular arrow), as determined by the expression state/profile of a host of genes within a developmental gene network (*X*_1_, *X*_2_, *X*_3_, …, *X*_n_), which is represented by the horizontal plane below the landscape. Three example genes within the larger network are shown, as they might appear at a particular stage of cell development. The gene regulatory interactions are the “forces” that shape the hills and valleys in the epigenetic landscape, propelling the progenitor toward development while constraining transitions among available phenotypes to particular channels (green arrows indicate normal progression toward separate developmental fates). A series of gene network states, with changes in expression of its individual genes, determine the topography of the development landscape. In this model, cell transformation is depicted as a diversion from the predetermined developmental channels, where self-renewal capacity is greatly diminished, into gene network expression states that create alternate, immature phenotypes with limited developmental potential and increased and perpetual self-renewal capacity (red arrows indicate abnormal progression due to transformation events).

For the purposes of this review, we have chosen the context of the “epigenetic landscape” metaphor in order to explore available experimental evidence for epigenetic pathways of toxicity leading to human cell transformation. The metaphor was developed over 60 years ago by the developmental biologist Conrad Waddington and our modification to the concept is presented in Figure 2 (see Text S1 for additional information on the origin of the epigenetic landscape metaphor and some recent applications related to human cell oncogenic transformation).

**Figure 3:**
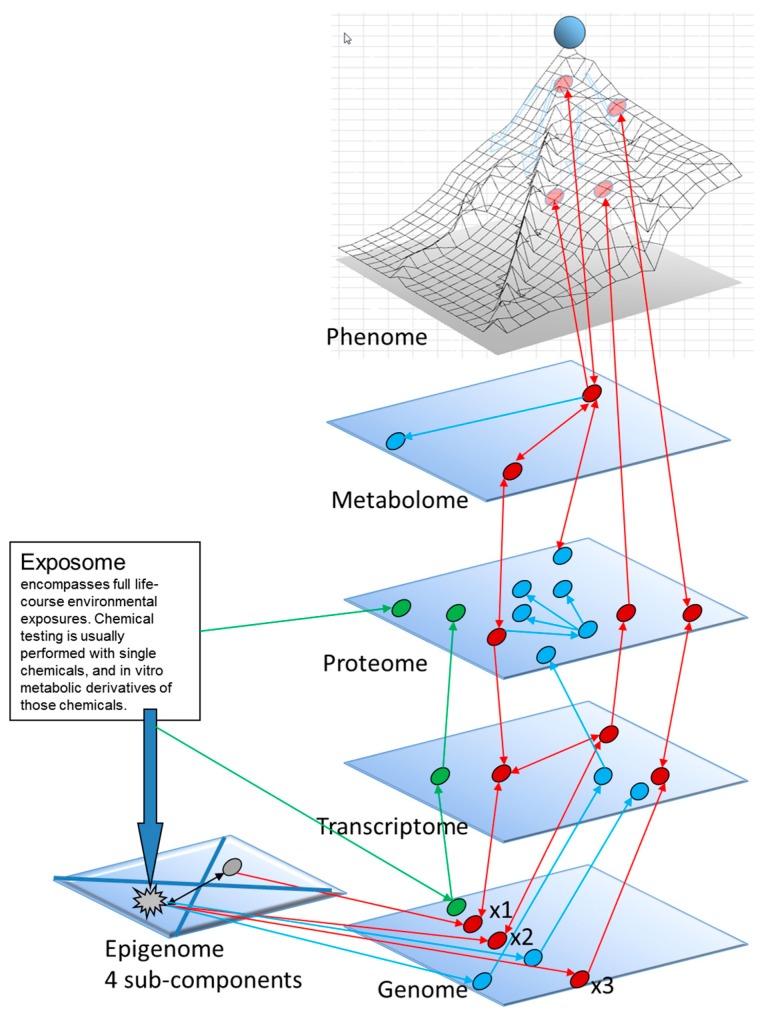
Hypothetical epigenetic pathway of toxicity pathways (showing key causal events) leading to adverse phenotypic changes in an experimental cellular system sensitive to changes among multiple “omic” levels and in keeping with Waddington’s metaphorical epigenetic landscape sub-structure, controlled by developmental genes. In this case, the target portion of the phenome consists of an epigenetic landscape determining the developmental fates of a tissue stem or progenitor cell (as drawn in Figure 2). In concert with one-another, the interacting “omic” domains participate in creating the observable “landscape” of physiological and molecular features that provide “shape” to an observable cellular phenotype. The epigenome, rather than being a single classification of molecular structure, is a functional categorization that is composed of several categories of molecular species. The four molecular sub-categories in the epigenomic domain considered in this review (see Figure 4) include protein, non-coding RNA and metabolite-derived covalent modifications of histones and DNA that have phenotypic consequences in cell transformation systems. The exposome potentially interacts at many places within the landscape, including the epigenome. The pathway schematic portrays an hypothetical situation in which chemically-induced perturbations to the epigenome subsequently propagate through the system-level domains, from gene transcriptional alterations, to perturbations in metabolite concentrations or structural protein expressions that are shown to affect or divert developmental choices at critical steps shaping cellular identity. PoT: A Pathway of Toxicity is a molecular definition of the cellular processes shown to mediate adverse outcomes of toxicants [21]. Pathway components: red, causal events; blue, consequential/associated events; green, coincidental events.

Our toxicity pathways-related view of the forces that can alter an epigenetic landscape is based upon a systems biology perspective (Figure 3), with key molecular perturbations that generate molecular linkages traversing several “omic” levels, ultimately leading to the development of adversely altered cellular phenotypes. Measurable key perturbations that are outside the ranges measured in particular in vitro human cell models and proven experimentally to be causal for subsequent phenotypic effects could be the basis for high throughput assays useful for Tox21 chemical screening.

**Figure 4:**
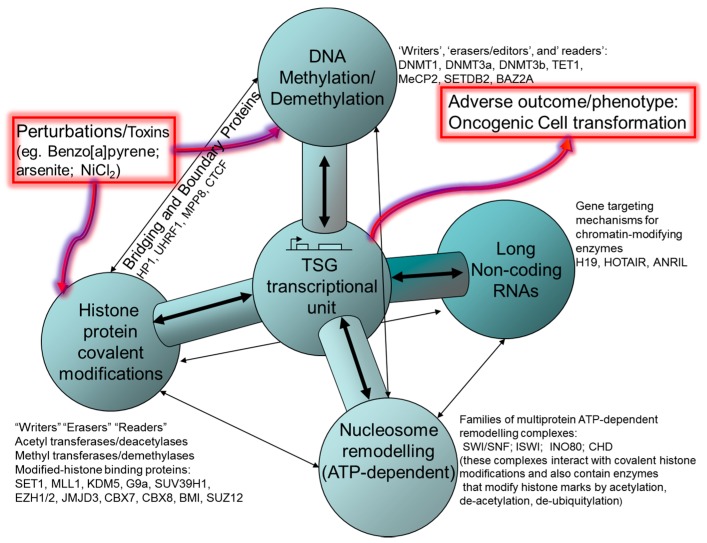
An epigenetic framework for pathways regulating persistent tumour suppressor gene transcription effects related to oncogenic transformation. The four main processes in the epigenetic framework acting upon the nucleosomes and DNA template of a gene transcription unit (larger arrows) are driven by multi-subunit protein complexes that act (1) to enzymatically modify DNA or (2) modify histone proteins, (3) remodel chromatin structure by moving nucleosomes along DNA and exchanging specific histones into and out of assembled chromatin, and (4) act via long non-coding RNA molecules to direct and anchor some of these complexes in a sequence-dependent manner. Smaller arrows indicate types of cross-talk among the four epigenetic processes in the framework. The framework does not imply a particular molecular structure, but represents the epigenetic “spheres of influence” that can impart persistent or even heritable functionality on transcriptional units. *TSG*: tumour suppressor gene (several examples are discussed in the review). The 23 specific components that act within the framework and for which there is experimental evidence confirming their functions as “drivers” of human cell oncogenic transformation are listed within the four spheres of epigenetic functions.

Over the last decade, the field of epigenetics has become more sharply focused and considerably strengthened by a rapidly accelerating number of molecular studies. Research activity is now centered on several interacting molecular domains closely associated with genomic DNA and acting to impart long-term, or heritable modulations of gene expression programs (networks) determining cellular identity. This extensive body of work has explored the elaborate network of molecular interactions forming the basis for chromatin-based heritable control of transcriptional functions. The present review does not cover related epigenetic functions controlling chromosome structure/stability, or influences on DNA repair capacity, nor does it discuss microRNA that functions in RNA silencing and post-transcriptional regulation of gene expression nor the operation of self-sustaining feed-forward transcriptional controls within transcription factor networks. The prevailing molecular paradigm for epigenetic control of gene expression is centered on the idea that transcription factor access to (or affinity for) the DNA helix is influenced by chromatin organization and compaction, which may be shaped by several interacting processes: DNA methylation, covalent histone modifications, substitution of histone variants or nucleosome remodeling and chromatin modulation by long, non-coding RNAs. For background on these individual topics in epigenetic controls, information has been provided in other overviews [36] and detailed multi-part reviews [37,38,39]. Herein are provided brief descriptions for histone post-translational modifications (HPTM) (Part 2), chromatin remodeling (Part 3), DNA methylation (Part 4), higher-order chromatin structures that control over epigenetic modifications (Part 5) and long non-coding RNA (Part 6), in order to add context to a number of examples of experimental or chemically-induced epigenomic perturbations affecting *TSG* transcription as a causal step in toxicity pathways in human cells. An organizing framework for the several epigenetic processes that could participate in stable alterations of gene expression in response to toxic or stressful events is presented in Figure 4, with an emphasis on the reciprocal molecular interactions (cross-talk) among the four main framework components.

## 2. Histone Post-Translational Modifications (HPTM), Histone Remodeling and Interaction with DNA Methylation Systems

The basic chromatin repeating unit is built upon histone protein families (H1 to H4), forming an octamer of four histone pairs (H2A, H2B, H3, H4) that is circled nearly twice by 147 bp of DNA helix. Covalent post-translational histone modifications are maintained enzymatically in a highly dynamic fashion [40] and contribute to the ability of chromatin to change and adapt in response to cellular events (e.g., DNA replication, DNA repair, transcription) or the demands of environmental, nutritional or other stresses. Several amino acids (lysine (K), arginine (R), serine (S) and threonine (T)) can be reversibly modified at more than 30 sites along the amino-terminal tails [41,42]. Many different post-translational modifications are known to occur (acetylation, methylation, phosphorylation, sumoylation, ubiquitination, ADP-ribosylation and biotinylation), each catalyzed by specific chromatin modifying enzymes. The added marks are removed by specific de-modifying enzymes (Table 1) and measurable levels of each modification reflect a steady-state balance between the actions of the two sets of enzymes (writers and erasers or editors, using the broad terminology that has been applied to several three-component molecular signaling systems in cells [43]). Transcription factors that respond to environmental signals, by interacting with key co-activator and co-repressors can, in turn, recruit or inhibit the various histone-modifying enzymes, and thereby contribute to the regulation and stable alterations of histone modifications at specific gene loci [44]. An important example is the epithelial-to-mesenchymal phenotypic transition, occurring during cancer cell progression, in response to various extracellular factors (WNT, Notch, TGF-β, hypoxia) comprising the active components of inducing microenvironments. The responses, at a molecular level, involve complex interactions among transcription factors and epigenetic regulators, acting in a deterministic manner to silence epithelial gene expression [45](reviewed in [46]).

Histone modifications have half-lives ranging from several minutes (acetylation) to up to several days (methylation) (summarized in [40]). Integrated effects of the various histone modifications influence histone-DNA interactions. For example, lysine acetylation (e.g., H3K16ac) or serine phosphorylation (e.g., H3S10p, H3S28p) reduce the net positive charge on the histone molecule. This may reduce local charge-dependent interactions of the histone tail region with nucleosomal DNA or adjacent histones, which can promote a more open chromatin configuration and facilitate access to DNA by the transcription machinery. Although lysine or arginine methylation would not affect histone protein charge, the increased size and hydrophobicity of the methylated amino acids can alter interactions between histones and other proteins. Histone modifications may form recognition sites for chromatin-binding, or reader proteins (the third component of the information system) that are thought to be the effectors/transducers of the HPTMs. The readers play roles in the organization of the extended chromatin proteome and thereby modulate transcriptional activity by enhancing or repressing the binding of specific DNA binding factors to their cognate DNA motifs [47]. These two mechanisms (reading and writing) are thought to work in combination to modulate gene expression.

Histone proteins provide both positive and negative influences on gene expression, with the direction of influence determined by the particular post-translational modifications on specific amino acid residues in the amino-terminal tails. The spectrum of the individual modifications distributed among the histones within a gene locus can serve as reasonable epigenetic indicator of chromatin state associated with gene activation or repression. For example, six classes of H3 modifications are most frequently assessed in epigenomic profiling experiments. Transcription start sites of actively transcribed genes are, generally, marked by trimethylated H3K4 (H3K4me3) and acetylated H3K27 (H3K27ac), while active enhancers can be identified by enrichments of both monomethylated H3K4 (H3K4me1) and H3K27ac. Gene bodies of actively transcribed genes are associated with trimethylated H3K36 (H3K36me3). Repression of gene expression can be mediated through two distinct mechanisms involving trimethylated H3K9 (H3K9me3) and trimethylated H3K27 (H3K27me3). The effects of de novo repressive methylation of H3K27 or H3K9 are of particular interest in relation to the subject of this review, because of their abilities to repress or silence *TSG* expression and their potential to be preserved as repressive marks over subsequent cell divisions. As well, there is growing ability to perform high-throughput screenings for enhancement or depression of the prevalence of these HPTMs and perturbed abundances of the mRNAs encoding “writers, readers and erasers” of these marks by chemicals (see Table 3, in Section 8, below).

Cross-talk is the norm between the HPTM and DNA methylation systems. This epigenetic combination confers to the chromatin segments specific structural and functional roles (e.g., centromere, DNA repeated elements, heterochromatin, euchromatin, gene promoters, intron-exon junctions, gene bodies), ultimately contributing to chromosomal stability and gene expression. DNA methylation, histone marks and nucleosome positioning interact and create chromatin regions of different accessibility for transcription factors and the transcription machinery [51,52]. The predominant marker of active genes is the presence of a Nucleosome Depleted Region (NDR) which consists of one or more missing nucleosomes upstream of the transcription start site (TSS) thereby providing access to the transcription machinery. Thus, active promoters have hypomethylated DNA, H3K4me3 marks and a NDR prior to the TSS [51]. Histone variants, such as H2A.Z, can also be found at active promoters. Genes that are in a poised/repressed state (in a bivalent region) have inactive promoters that can either show DNA hypo or hypermethylation, but with densely packed nucleosomes and with the co-occurrence of the H3K27me3 and H3K4me3 marks. Finally, genes that are silenced have DNA hypermethylation, densely packed nucleosomes and the H3K27me3 marks.

Imbalances in histone post-translational modifications are considered to be a molecular-level hallmark of most cancer types [53]. The idea that perturbations of the histone modification pathways could be contributory mechanisms in chemical carcinogenesis is circumstantially supported by several studies that have shown direct effects of organic and metal carcinogens on writing and erasing enzyme activities or abundances (e.g., histone acetyltransferase, histone deacetylase [54,55,56,57], KDM3A/JMJD1A H3K9 demethylase [58,59,60], G9A H3K9 methyltransferase [61], lysine methyltransferases [62]). However, downstream epigenetic changes found in a tumour or a tumour cell line or in chemically-exposed precursor cells are not considered as evidence for their causality in chemically-induced carcinogenesis. The epigenetic changes may be either a non-functional consequence (or branch) of the mechanistic pathway that leads to oncogenic effects or may be mechanistically unrelated, co-incidental events. The following sections of this review highlight experimental molecular biology that provide authentication for causal links among (1) experimentally- or chemically-induced epigenetic changes, (2) silencing of *TSG*s by repressive epigenetic factors functioning in various combinations and (3) induced oncogenic phenotypic effects in human cell culture models. Criteria for types of experimental evidence that can contribute to establishing causality among epigenetic pathways leading to cell transformation and carcinogenesis are outlined in Table S1 and form the basis for the selection of many of the experimental studies included in this review.

### 2.1. Polycomb and Trithorax Group Proteins

Many of the enzymes catalyzing histone modifications are contained within evolutionarily-conserved, multi-subunit protein complexes that function as gene silencers or activators. Classic examples of these complexes are the polycomb group (PcG) proteins, discovered in *Drosophila*, and typically associated with silent gene transcriptional states. The more heterogeneous trithorax group (TRXG) protein complexes act to maintain their target genes in active transcriptional states and are defined genetically as antagonists to PcG-protein complexes. TRXG protein complexes may be sub-classified either as histone modifiers or as nucleosome remodelers. Disruptions to the dynamic interplay of the opposing actions of PcG and TRXG, such as excessive PcG or compromised TRXG activities, can contribute to tumourigenesis [63].

PcG proteins are part of a widely studied developmental model of epigenetic silencing machinery in mammalian cells that creates transcriptionally repressive histone modifications controlling developmentally important genes [64]. In a temporally-regulated manner, the PcG repressive protein complexes PRC1 and PRC2 function, in concert, to control activation or repression of thousands of target genes associated with stem cell pluripotency, lineage commitment and progenitor cell differentiation [65,66,67].

The PRC1 complex is thought to exist in many variant forms due to combinations of the large number of subunit homologues in mammalian cells. The complex contains five different subunit types: (1) The polyhomeotic protein (HPH1, HPH2, HPH3); (2) The polycomb-/CBX protein (HPC1/CBX2, HPC2/CBX4, HPC3/CBX8, CBX6 and CBX7) recognizes the H3K27me3 mark through chromodomains. (Note that the H3K27me3 mark, written by the PRC2 complex, contributes to the binding of PRC1 complexes that, in turn, ubiquitinylate histone H2A and the modified polynucleosomes compact as a consequence [68,69]); (3) RING1- and 2- (RING1A/B) catalyzes ubiquitinylation of histones and other proteins; (4) A posterior sex comb protein (BMI1, MEL18, MBLR and NSPC1) required to stimulate the E3 ubiquitin-protein ligase activity of RNF2/RING2; (5) A sex comb on midleg (SCML1–2) zinc-finger protein.

PRC2 is a multi-component protein complex including EZH2, EED, SUZ12, and RBBP4. EZH2 (enhancer of zeste) is the well-studied methyltransferase that trimethylates histone H3 on lysine 27 (H3K27me3). EED (embryonic ectoderm development) is required for the methyltransferase activity of EZH2 and binding to the H3K27 site in the histone tail region [70]. A nucleosome binding module is formed by suppressor of zeste-12 homolog (SUZ12) and histone-binding retinoblastoma binding protein 4 (RBBP4), which functions as an adaptor to hold EZH2 in place. Cross-talk with DNA methylation pathways is facilitated by the PRC2 complex, via the EZH2 histone methyltransferase, which directly recruits DNA methyltransferases and is required for DNA methylation of EZH2-target promoters in human tumour cell lines [71].

Trithorax H3 methyltransferases participate in the long-term maintenance of activated gene transcription through enzymatic trimethylation of H3K4 (H3K4me3) at specific sites in chromatin recognized by the complex. In mammalian cells, the Trithorax H3 methyltransferases are categorized according to their single catalytic subunit that harbors a conserved SET domain. Six family members are known: SET1A/SET1B and four MLL-family H3 methyltransferases. MLL and SET1 complexes all contain three structural components, RBBP5, ASH2L and WDR5. The WDR5 subunit mediates interactions between the MLL1 catalytic unit, its H3 substrate and the other structural subunits [72].

#### 2.1.1. Evidence for Causal Roles of Polycomb Complex H3K27 Methyltransferase Activities in Oncogenic Transformation of Human Cells

Among the well-known polycomb targets in the genome are CpG islands in genes encoding proteins known for their participation in multiple roles: (1) intercellular signaling factors, such as WNT, TGF-β, FGF, BMP; (2) tumour suppressor proteins, for example, p16/IINK4A/CDKN2A and p15/INK4B/CDKN2B at the *INK4-ARF* gene locus; (3) stem cell transcription factors, important for maintaining pluripotency in embryonic stem cells (NANOG, OCT4, SOX2) (Polycomb-mediated silencing of such pluripotency factors allows cellular differentiation to proceed. These factors are employed in induced pluripotent stem cell (iPSC) reprogramming where they are considered to be “pioneering” factors, capable of opening the compacted chromatin of silenced genes); (4) Members of the Homeobox (HOX) transcription factor family of about 40 members that includes the four *HOX* gene clusters located on chromosomes 2, 7, 12, and 17 as well as the many more unclustered homeobox genes, such as *ENGRAILED*, *SIX*, *LHX*, *PAX* and *DLX*.

##### PcG Targets: Homeobox Genes, the “Bivalent State” and Epigenetic Switching during Carcinogenesis

The homeobox transcription factors play decisive roles during embryogenesis and in differentiation of adult cells. Normally, their target gene promoters are maintained, in embryonic stem cells, in a “bivalent” state with the chromatin at each gene typically displaying a combination of active, Trithorax-mediated (dimethylated H3K4) and repressive PcG-mediated (trimethylated H3K27) histone marks. The resultant chromatin state usually silences or reduces gene expression of these differentiation genes, in order to maintain “stemness” in pluripotent and multipotent stem cells. This “poised” chromatin state can be rapidly reversed to an active expression state when appropriate developmental signals trigger a cellular differentiation program and at later points during cell lineage progression. Similar to homeobox genes, greatly reduced *p16/INK4A* tumour suppressor gene expression seems to be a hallmark of different kinds of stem cells (both embryonic and adult/tissue/somatic), wherein epigenetic silencing does not result from enhanced DNA-methylation of the *INK4A* gene promoter, but is associated with the “bivalent chromatin” state [73].

Homeobox genes are more likely than other non-PcG targets to display cancer-specific gene promoter DNA hypermethylation, compared to untransformed tissue cells. In cancer cells, during the phenomena known as “epigenetic switching” (or 5mC reprogramming), the H3K27me3 mark in the promoters of bivalent polycomb target genes is replaced by, or becomes accompanied by, de novo DNA methylation. The added DNA methylation is thought to generate a more permanent form of gene silencing than is provided by the bivalent histone methylation state found in stem cells [73,74,75,76,77]. Many of the PcG target genes assessed in these studies are known to function as cell proliferation inhibitors. They either promote cell adhesion or function as antagonists of the WNT signaling pathway controlling growth and differentiation of stem cells. Therefore, suppressed epigenetic signatures among the PcG target genes may provide useful biomarkers for the oncogenic state in various tissues.

A mechanism underlying cancer-associated epigenetic switching events has been proposed to act via the influence of intracellular oxidative stress on redistribution of repressive histone modification machinery across the genome. It was shown in human embryonic carcinoma cells in vitro that H_2_O_2_-induced oxidative stress reduced transcription from thousands of genes with CpG islands or high G/C content in their promoter regions, including several PcG targets. The reduced gene expression levels of the PcG targets were linked to aberrant DNA hypermethylation, increased repressive H3K27me3 marks and loss of activating H3 acetylation at the respective gene promoters [78]. The stress-induced changes to the epigenetic marks were coincident with increased 8-oxo-2′-deoxyguanosine (8-oxo-dG) adducts, recruitment of gamma-H2AX (hypothesized to indicate oxidant-induced DNA base damage), and a silencing complex composed of SIRT1 histone deacetylase (a component of PRC4 silencing complex in stem and cancer cells [79]), as well as the PRC2 EZH2 histone3 methyl transferase and its interacting DNMT1 and DNMT3B enzymes. Preliminary evidence also pointed to the possibility that recruitment of the DNMT enzymes to G/C-rich promoters may be facilitated by OGG1, the glycosylase that removes oxidized deoxyguanosine (8-oxo-dG) during DNA base excision repair. These molecular changes and concomitant DNA hypermethylation were especially pronounced among PcG targeted genes that had constitutively low basal expression and that frequently become hypermethylated in human tumours. It was suggested that in situations of chronic oxidative stress among susceptible cells in a population, such molecular/epigenetic perturbations would lead to creation and spread of methylated DNA sites within poised or repressed gene promoters and thereby, lead to a more persistent silencing of the affected genes, while actively transcribed genes could more rapidly resume full expression after resolution of the oxidative stress state. Subsequent work has shown that H_2_O_2_ treatment induced interaction between DNMT1 and mismatch repair proteins, MSH2–MSH6 and recruitment to damaged DNA, and repression of expression from genes with CpG island promoters [80]. Although cell phenotypic effects were not investigated in these studies, a cellular toxicity pathway relevant to carcinogenesis could be expected to lead from these molecular steps involving PcG function to targeted repressive effects on critical tumour suppressor genes, such as *p16/INK4A* and *p14/ARF*. Indeed, increased 8-OHdG adducts were shown to be highly correlated with increases in the repressive H3K27me3 mark in the promoter sequences of 27 tumour suppressor genes, including *p16/INK4A*, in HepG2 and primary human liver cells treated with H_2_O_2_ [81]. Persistently repressed tumour suppressor gene expression resulting from the perturbed distribution, activity and interactions of these epigenetic modifiers would constitute key, epigenetically-heritable molecular changes with the capacity to generate cancer-related phenotypes likely to endure in measurable fractions of cells in chemically-treated cultures.

Changes to the histone modification landscape and the DNA methylation patterns within a genome are known to be interdependent. Demonstrations of functional crosstalk between these two epigenetic controls have been revealed through experimental modifications of the abundances or activities of the TRXG, PcG histone methylation or DNA methylation enzymes. Such experiments have produced global redistributions of the various epigenetic marks across the genome following depletion of any one of the others. Moreover, there are preferential sites for change within promoter CpG islands in many Polycomb target genes [82,83], especially when the native chromatin at a locus is marked with both PRC2 and PRC1 histone marks, H3K27me3 and H2AK119ub [84]. In another approach, a novel, single-DNA molecule, flow-cytometric analysis using antibody probes to detect specific epigenetic marks on small genomic DNA fragments demonstrated a loss of the antagonism between 5mC placement in DNA and polycomb-related H3K27me3 marks found in normal cell chromatin fragments, as compared to the chromatin from immortalized or *Ha-RAS/v-MYC* oncogene-transformed 3T3 mouse fibroblasts and in human promyelocytic leukemia cells [85]. Reduction of 5mC levels in response to the DNA methylation inhibitor 5-aza-2′-deoxycytidine resulted in opposite responses of the H3K27me3 marks in the normal cells compared to transformed cells. In the normal cells, H3K27me3 increased upon reduction of 5mC, (i.e., antagonistic placement of the two epigenetic marks), while in transformed cell states, H3K27me3 decreased along with the reduction of 5mC. These results pointed to a breakdown in the coordinated control of DNA and histone methylation that may be an early event in carcinogenesis, perhaps related to epigenetic switching events. This loss of antagonistic placement of the two epigenetic marks was also demonstrated using immunoprecipitation of methylated DNA and H3K27me3-marked chromatin to show that the fraction of genes with the dual modification was increased 10- to 20-fold in cancer cell lines as compared to primary tissue cells and immortal cell lines [86]. In this study, enhanced re-expression of genes with the dual modification, including several tumour suppressor genes, was obtained after combined treatments with an inhibitor of DNA methyltransferase and of EZH2. Notably, the combined treatments produced an additive inhibitory effect on cancer cell growth in vitro.

##### Links between the pRB/p16 Tumour Suppressor Pathway and PRC2, PRC1 Proteins

**Figure 5:**
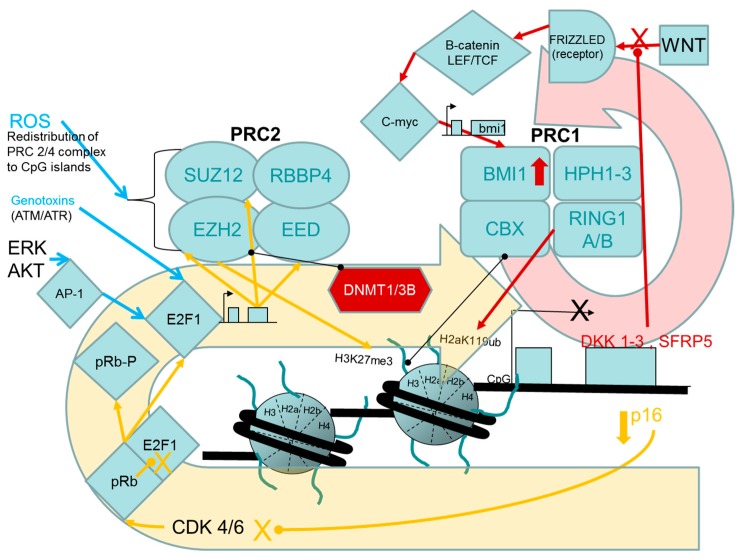
Examples of two pathways of histone modification by polycomb complexes leading to suppression of selected *TSG*. Two potentially reinforcing pathways (one pathway leading from experimentally up-regulated *BMI1* expression, in red connecting arrows, and the other pathway leading from experimental down-regulation of *p16* the gene, in orange connecting arrows, respectively.) that could lead to stable *TSG* repression are mapped. Vertical (up or down) arrows beside pathway components indicate experimental manipulations that resulted in *TSG* suppression (*p16* down-regulation, in orange, *BMI1* up-regulation, in red, at the origin of the two large arrows indicating the pathways of subsequent effects) (note increased H3K27me3 levels at the *p16/INK4A* gene promoter following from its engineered down-regulation were not confirmed, although other gene promoters were affected. In other cell lines, over-expression of PRC2 components resulted in *p16/INK4A* repression). Several points at which chemical perturbations affect the pathway and mentioned in the text of the review are also indicated (blue arrows). The two reinforcing epigenetic pathways were constructed from information in: [87,89] (small connecting arrows and large Xs indicate the normal forward or blocking activities, respectively, within the pathways that were changed due to p16 down-regulation or BMI1 up-regulation. AP-1 Activator protein 1 transcription factor; ATM, Ataxia telangiectasia gene mutated serine/threonine kinase; ATR, ataxia telangiectasia and Rad3 related serine/threonine kinase; AKT, serine/threonine kinase, protein kinase B; CDK 2/4, cyclin-dependent kinases 2 or 4; ERK, extracellular regulated MAP kinase; E2F1, E2F transcription factor 1/retinoblastoma-associated protein 1; DKK1-3, dickkopf WNT signaling pathway inhibitors 1-3; LEF/TCF, lymphoid enhancer binding factor/T cell-specific transcription factor; pRB, retinoblastoma protein; pRB-P, phosphorylated retinoblastoma protein; ROS, reactive oxygen species; SFRP5, secreted frizzled related protein 5; WNT, wingless-type MMTV integration site.

Beyond the correlative associations between changes among polycomb marks, DNA methylation and phenotype, there is work in which direct experimental manipulations of PcG protein activities has begun to reveal causal connections between the activities of PcG proteins and the expression of oncogenic phenotypes in human cells. Loss of expression among polycomb-targeted growth control genes is considered to be a driver of uncontrolled cellular growth, perhaps more so when these genes are subjected to the perpetual DNA methylation-associated silencing subsequent to an “epigenetic switch” event that is often found in human tumours. These two underlying mechanisms of carcinogenesis were demonstrated experimentally by indirectly creating up-regulated expression of PcG proteins in human mammary epithelial cells [87]. The following description of this work is illustrated in Figure 5. An engineered reduction in *p16/INK4A* gene expression permitted the activation of E2F1 transcription factor due to increased phosphorylation of pRB and release of bound E2F1. The unbound E2F1 is a known upstream inducer of the PRC2 components EZH2, EED and SUZ12 [88]. As expected, short hairpin RNA (shRNA)-mediated knockdown of *p16/INK4A* expression in the mammary epithelial cells caused up-regulated expression of the E2F1-regulated PRC2 proteins EZH2 and SUZ12, independent of proliferation status of the cells (cycling or quiescent). The knockdown of *p16/INK4A* expression resulted in a targeted repression of the polycomb target gene *HOXA9* and subsequent DNA hypermethylation of an upstream control region in this gene. HOXA9 transcription factor is a positive regulator of cell fate determination and terminal differentiation. DNA hypermethylation at the upstream control region was shown to be dependent on the simultaneous over-expression of EZH2 and SUZ12 proteins in these cells, which, along with DNMT1, DNMT3A and DNMT3B, were found to be enriched at the *HOXA9* regulatory region. Because repression of *p16/INK4A* expression can induce some of its own epigenetic repressors through activation of the E2F1 transcription factor, there is potential for the development of a self-reinforcing negative feedback loop that could tend to stabilize polycomb suppression of *p16/INK4A* and perhaps other genes such as *HOXA9* in this cell system, although the status of the endogenous *p16/INK4A* chromatin was not actually addressed in this study.

In summary, it is likely that cells enter a state of epigenetic plasticity upon loss of a functional p16/pRB pathway and that subsequent DNA methylation events may follow, downstream of PcG-mediated histone modifications, resulting in overt transcriptional repression of susceptible genes. It is notable that numerous carcinogenic chemicals have been reported, in the Comparative Toxicogenomics Database, to reduce p16/INK4A mRNA and/or protein expression in human cells [90]. Taken together, these studies suggest that consideration should be given to the idea that chemical “knockdown” of *p16/INK4A* expression could be the origin of a toxicity pathway with potential to produce long-lasting, self-perpetuating, epigenetic suppression of various *TSG*s via E2F1-mediated increases in PRC2 activity.

Table 2 lists several experiments in which the functional contributions of other PcG components acting on the *p16/INK4A* locus and results for cell growth regulation were directly demonstrated. The four studies are reasonably good examples of experimental investigations of causality (Table S1) for epigenetic control of cell transformations, in that changes to specific epigenetic reader/writers/erasers were determined by specific molecular tools, the expected changes were usually demonstrated within the *p16/INK4A* locus and the growth behavior of the cells could be linked those molecular changes. These and many other examples of studies on other epigenetic controls, that follow in the other sections of this review, show that the *p16/INK4A* locus is the most well-understood TSG target for various forms of epigenetic regulation across different cell models.

These studies employed stable transfections of vectors expressing shRNA or ectopically expressed genes to silence or up-regulate, respectively, components of PRC2 (SUZ12) or PRC1 (CBX8, CBX7, BMI1) protein expression which resulted in effects on *p16/INK4A* tumour suppressor gene expression and proliferative ability in several primary human cell models [91]. These results revealed some of the key molecular players that control the well-known senescence-type growth arrest phenotype that eventually overwhelms cultures of primary human cells. The arrested state arises when cellular stresses (ROS, stalled replication forks, telomere erosion, stromal disorganization) generated by in vitro culture conditions, or by activated oncogenes Those stresses initiate a complex series of molecular controls in primary, diploid cells that function to increase the activities of the p16/CYCLIN D/pRB and/or the p14ARF/MDM2/p53 tumour suppressor pathways, leading to impaired G1- to S-phase cell cycle progression (reviewed in [34,35]) (Figure 1).

The studies consistently showed that engineered manipulations of the polycomb components were effective controls on the extended lifespan and growth rates of the several different primary cells studied. Not all studies also measured the effects on changing the epigenetic marks and/or PcG components that were bound to the target *p16/INK4A* locus. Ultimately, this is important information in order to develop the full understanding of these particular pathways of epigenetic toxicity. It is also important to note that the changes in abundances of the PcG components were all created via stable transfections of expression constructs targeting the respective components. It would be very interesting to explore, in the context of simulating chemical exposures, if more transient modulations in the expression of the PcG components would produce lasting effects on *p16/INK4A* expression and growth phenotypes in any fraction of the experimentally modified cells.

An interesting outcome in the last study listed in Table 2 was that DNA methylation was inconsistently present within the *p16/INK4A* gene promoter in cell clones fully immortalized by simultaneous *BMI1* and telomerase overexpression in HMEC, suggesting that in this case, the BMI1 activity, alone, may have been sufficient for functional *p16* repression, without the necessity for DNA methylation. The epigenomic state of the *p16/INK4A* locus in the extended lifespan cells may be similar to that of other silenced tumour suppressor genes in immortalized cells, such as human MCF7 breast cancer cells, in which increased abundance in H3K27me3 marks and, in some cases, increased BMI1 binding, were frequently associated with relatively low levels of promoter DNA methylation. [95]. Therefore, PcG-mediated suppression, alone, is likely to be operative in chemical-stress-induced suppression of *TSG*s during chemical carcinogenesis and underscore a need for development of methodologies that can detect these silencing mechanisms within treated populations.

##### Experimental Support for the Participation of PRC2 EZH2 H3K27 Methyltransferase in Driving the Progression of Oncogenic Phenotypes beyond the Extended Lifespan and Immortalization Stages

The following two paragraphs are supported by Figure 5. Overexpression of *EZH2* in immortalized breast epithelial cells and a prostate epithelial cell line induced anchorage-independent growth in vitro and produced an invasive phenotype when measured with in vitro and in vivo assays [96,97], although participation of the H3K27me3 product at specific gene promoters was not directly assessed in these studies. In immortalized human hepatocytes, ectopic over-expression of *EZH2* enhanced cellular proliferation in vitro, due to PcG-mediated suppression of several inhibitors of the WNT/β-CATENIN signaling pathway [98]. This result was consistent with the observation that up-regulated *EZH2* expression is frequently detected (42%) among hepatocellular carcinoma cells (HCC) and that their growth rate was reduced in small interfering (siRNA)-mediated *EZH2* knockdown experiments. The increased growth rate of HCC in vitro was related to transcriptional repression due to increased occupancy of PcG components and histone deacetylase 1 (HDAC1) at the promoters of WNT-inhibitor genes, along with relative increases of the H3K27me3 repressive mark and decreases of the H3K9ac activating mark. Similarly, phenotypic reversions of cancer phenotypes have been accomplished by the targeted knockdown of EZH2, with subsequent effects on H3K27me3 levels and target gene expression, in studies on a variety of tumour cells [95,99,100,101].

Over-expression of the BMI1 component of PRC1, in a human teratoma cell line, increased cell proliferation and induced loss of contact inhibition, manifested as dense foci in monolayer cultures, in vitro [76]. In the *BMI1* over-expressing cells, DNA methylation increased in the WNT antagonist gene, *SFRP5* (Secreted frizzled-related protein 5), as the culture passages increased, perhaps modelling similar hypermethylation of this gene that has been catalogued among human tumours. Recently, a multi-component, autoregulatory loop of *BMI1* overexpression was described in human mammary MCF10A cells, in which it was shown that BMI1 could indirectly transactivate its own promoter via the increased c-MYC transcription factor activity that followed a BMI1-induced activation of the WNT pathway, resulting from the BMI1-mediated repression of the WNT antagonist genes, *DKK1-3*, and probably involving PRC2 to create H3K27me3 marks [89] (Figure 5). The *BMI1* over-expression resulting from this cascade was demonstrated to increase the ability of the cells to form mammospheres in culture, a manifestation of a cancer stem cell phenotype that was antagonized by DKK1 inhibitor expression, in this cell model. Since many breast cancer cells express relatively high levels of BMI1, it is possible that this positive feed-back loop participates in maintaining that enhanced expression state and the cancer cell phenotype.

In addition to gaining extended replicative capacity, primary human epithelial cells and cell lines may also gain enhanced invasiveness and anchorage-independent growth characteristics upon experimental overexpression or sustained up-regulation of *BMI1* [102,103]. In primary nasopharyngeal cells, the *PTEN* (Phosphatase and tensin homolog, a protein tyrosine phosphatase and tumour suppressor gene), is a direct binding target of over-expressed BMI1 gene product, resulting in reduced *PTEN* expression that was comparable to the BMI1-induced reduction of *p16/INK4A* expression. PTEN is a negative regulator of the PI3K/AKT pathway through its dephosphorylation of the inositol triphosphate activator of AKT, a tyrosine kinase that participates in a wide range of cellular behaviors, including: regulation of the cell cycle, proliferation, apoptosis, cell adhesion, epithelial-to-mesenchymal transition (EMT) and cancer progression. It was shown in this study that gain of AKT activity upon BMI1-mediated repression of *PTEN* resulted in stabilization of the EMT-associated, SNAIL transcriptional inhibitor protein and consequent down regulation of its target, *E-CADHERIN*, ultimately leading to the invasive cellular phenotype.

##### Stress- and Chemically-Induced Expression of PcG Proteins

Chronic up-regulation of EZH2 histone methyltransferase is found in a variety of human tumours [79]. In human colonic tumour cell lines, up-regulation results from stress and oncogene-activated signaling pathways involving protein kinases ERK, AKT and the AP1 transcription-factor. The increased expression of *EZH2* plays a causal role in the enhanced invasive capacity of these cell lines, possibly through H3K27me3-mediated suppression of key genes functioning in anchorage-dependence and contact inhibition regulation of cell migration [104]. It is also possible that activation of the AKT pathway by increased BMI1 abundance (discussed above) could be a reinforcing condition for *EZH2* expression and repression of polycomb gene targets. Considering that elevated expression of histone modification proteins appears to be a driving effect for the oncogenic phenotype, it is interesting to note that exposure of human cells to several chemical stressors and carcinogens can increase expression of EZH2, as well as SUZ12 and BMI mRNAs or proteins (Comparative Toxicogenomic Database [90]). As discussed above, in the context of experimentally repressed *p16/INK4A* expression, *EZH2* and *SUZ12* expression are also positively controlled by the E2F1 transcription factor, which may be induced and stabilized by agents inducing genotoxic stress [105]. Chemical stress responsiveness presents the intriguing possibility that, in situations of chronic exposure, up-regulation of PcG proteins, outside of their tightly controlled expression within an unperturbed cell cycle, could increase the density of repressive H3 modifications at susceptible gene loci. Future research focused on PcG protein activities will be necessary to more fully determine (1) whether the documented gene expression increases induced by a broad range of chemical agents are mediated by activation of the aforementioned, transformation-linked, stress-response pathways; (2) if the chemically-induced gene expression increases in PcG proteins are sufficient to result in polycomb-mediated suppression of polycomb-targeted *TSG*s; and (3) whether or not the increased target gene suppression subsequently leads to PcG-directed DNA methylation and long-term (over multiple cell generations) tumour suppressor gene silencing contributing to transformed cell phenotypes in any fraction of cells in chemically exposed cell cultures.

#### 2.1.2. Evidence for Causal Involvement of Trithorax H3K4me Histone Modifying Complexes (MLL/SET1 Complex) in Oncogenic Transformation

The H3K4me activating mark leads to up-regulation of certain developmental genes, including *HOX* family members. There are six related H3K4 methyltransferase complexes in humans, each with a distinct, but conserved catalytic subunit (MLL1, MLL2, MLL3, MLL4, hSETD1A, hSETD1B). The SETD1/MLL catalytic subunit interacts with the three other core complex components: WDR5, ASH2L, and RBBP5 to provide the minimal subunit composition required for H3K4 methylation [72].

The *p16/INK4A* locus is epigenetically regulated by both PcG H3K27me3 repressive marks and by TRXG H3K4me3 activating marks. In human diploid fetal lung fibroblasts (WI-38/E7 expressing HPV E7 oncogene inhibitor of pRB, thereby creating an oncogene-induced stress), the sequestration of active pRB by E7, reduces pRB-mediated recruitment of PcG proteins to the *p16* locus and removes PcG-mediated repression, whereby *p16* becomes strongly expressed. The up-regulated *p16* expression is accompanied by an increase in the H3K4me3 mark in regions surrounding the *p16/INK4A* transcription start site and globally across the genome [106]. Knocking down *MLL1* methyltransferase or the structural RBBP5 subunit of the MLL1 complex reduced both p16/INK4A mRNA and protein levels (as well as those for p18/INK4C tumour suppressor), supporting direct action of the methyltransferase on up-regulating *p16* expression. MLL1 methyltransferase activity and binding to the *p16/INK4A* promoter was found to be facilitated by DDB1 (UV-induced DNA damage binding protein) and by CUL4 which are part of an E3 ubiquitin ligase complex. Induction of *p16/INK4A* expression by forced *Ha-RAS* oncogene expression (creating oncogene-mediated replicative stress) was also dependent on the active MLL1 methyltransferase and its associated proteins in the complex. Thus, the MLL1 complexes mediate *p16/INK4A* activation during the oncogene-stress checkpoint response (and during normal cell ageing) that is repressed by the PcG proteins in the cells of developing tissues and embryos. Further along in the p16-pRB pathway, the H3K4me3 demethylases JARID1A, JARID1B are contributors to pRB-linked silencing of E2F-regulated genes during replicative and oncogene-induced senescence in primary human IMR90 cells. Their simultaneous knockdown compromises H3K4 demethylation and silencing of a pRB-E2F target gene subset during senescence [107]. It is conceivable that some chemical exposures could interfere with the assembly and activity of these gene activating complexes on the p16 checkpoint gene or hinder the removal of the H3K4me3 activating mark on E2F-driven cell cycle genes, leaving cells with reduced senescence-checkpoint function and open to the effects of oncogene activations.

In various human malignant cell lines, in which it is likely that the p16-pRB pathway has been compromised by either genotoxic or PcG-mediated silencing, and in which MLL1 complex activity is increased, gene knockdown experiments have shown that *MLL1* is a key player in maintaining several aspects of the malignant phenotype, including cell proliferation, tumour growth, hypoxia signaling and angiogenesis in vivo. As one example, *MLL1* knockdown with antisense oligonucleotides, in the HeLa cervical cancer cell line, resulted in decreased cell cycle progression and decreased expression of various cell-cycle regulatory genes (*CYCLIN A* and *B*, *p57/KIP2*), associated with decreased histone H3K4me3 and RNA polymerase II recruitment at their promoters [108]. The dependence of the oncogenic phenotype was also investigated in vivo. The growth of a cervical cancer xenograft into nude mice was strongly inhibited by systemic administration of *MLL1* antisense oligonucleotides.

It is known that H3K4 methyltransferase SETD1A and H3K4me3 marks are generally up-regulated in human colorectal tumours. When a vector-mediated shRNA knockdown of *SETD1A* in HCT116 and SW84 colonic tumour cell lines was performed, in vitro, the H3K4me3 mark was globally reduced (but not H3K4me2 or H3Kme1 marks). Cell growth and anchorage-independent colony formation were also inhibited by the knockdown [109]. This reduced growth capacity was accompanied by over 6000 genes with differential expressions, with approximately half of the WNT pathway target genes (56/113) being affected. The large effect on the WNT pathway genes was significant given that it is known that the WNT-signalling pathway is affected in the majority of human colorectal cancers. It was shown in this work that SETD1A interacts with β-CATENIN transcriptional activator of the WNT pathway. *SETD1A* knockdown decreased the H3K4me3 mark and binding of the SETD1A complex (containing ASH2L, RBBP5, WDR5) to the *c-MYC* gene promoter, and other WNT-responsive genes. Thus, up-regulated *SETD1A* and its H3K4me3 product probably play a causal role in colorectal tumour cell growth by dysregulating the WNT signaling pathway. Similar gene knockdown experiments in the HeLa tumour cell line have shown that the MLL1 complex component, ASH2, that is over-expressed in many human cancers, also contributes to a growth advantage measurable in in vitro experiments [110].

### 2.2. Evidence for Causal Participation of H3K9 Methyltransferases in Oncogenic Transformation of Human Cells

The methylation of H3K9 residues provides a parallel pathway to H3K27 methylation catalyzed by PcG EZH2 methyltransferase and this additional repressive mark may act synergistically to promote inactivation of tumour suppressor genes. Methylation of H3K9 in human cells is mostly catalyzed by SUV39 (suppressor of variegation 3–9 homolog family) lysine methyltransferases SUV39H1, SUV39H2, GLP /EHMT1, G9A /EMHT2, SETDB1/ESET and SETDB2, and non-SUV39 enzymes PRDM2 and ASH1L [111]. It has been known for over a decade that complex interactions exist between histone lysine methylation, DNA methylation and other chromatin modifications. H3K9 methylation can recruit DNMTs that will subsequently methylate DNA and, reciprocally, CpG methylation in DNA may serve as a signal for establishing histone modifications.

H3K9 methylation is thought to reinforce DNA methylation in the repression of gene expression. This function may derive from the several H3K9 modification enzymes (SUV39H1, SETDB1, G9A) that are able to interact directly with the de novo DNA methyltrasferases (DNMT 3A/B), which could facilitate new DNA methylation within the gene loci where the histone methyltransferase enzymes are active. The G9A histone methyltransferase also associates with the DNA maintenance methyltransferase, DNMT1, during replicative DNA synthesis (reviewed in Rose and Klose [112], Fuks [113], Brenner and Fuks [114]).

**Figure 6:**
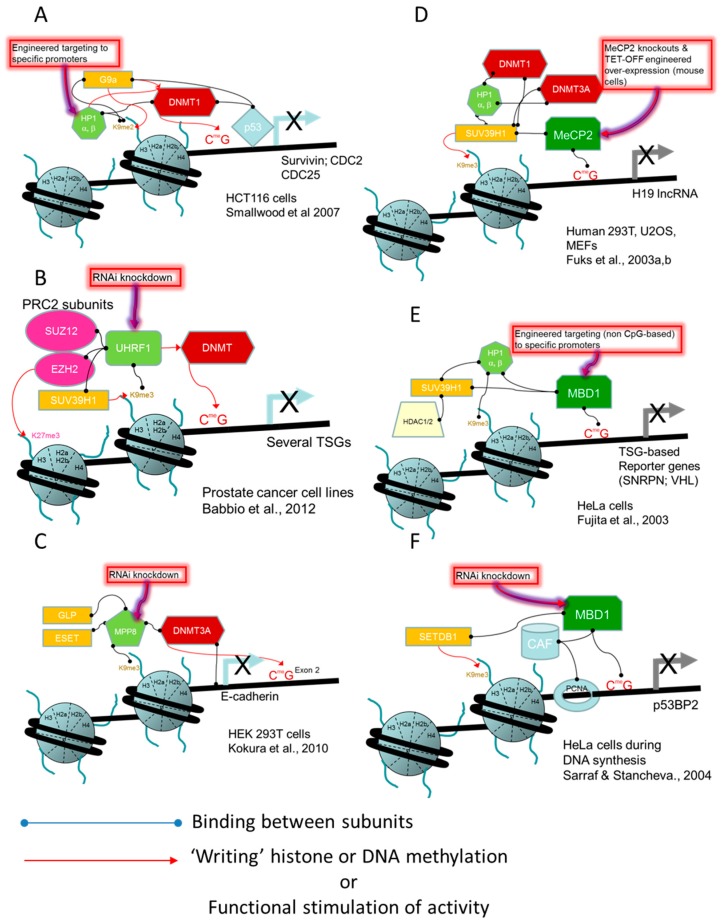
(**A**–**C**) Schematic models depicting “bridge” proteins (green boxes, HP1, UHRF1, MPP8) connecting H3K9 histone methylation to H3K9 methylation writers (orange rectangles) and to DNA methyltransferases in segments of DNA controlling tumour suppressor gene silencing (X over transcription unit, ↱, indicates inhibited transcription). Models in (**D**–**F**) depict the tethering of H3K9 histone methylation writers to methylated segments of DNA in *TSG*s via methylated DNA binding and “bridge” proteins (dark green boxes, MeCP2 and MBD1). Experimental interventions are indicated in the Red Boxes within each schematic. Several forms of evidence were presented for the interactions of components in each model, as indicated by the types of connectors at the bottom of the figure.

In addition to direct protein-protein interactions, histone methyltransferases and DNA methyltransferases may also interact through “bridge” proteins such as HP1 and UHRF1. Heterochromatin protein 1 (HP1, α, β) acts through its chromodomains as an adaptor protein for H3K9me2/3, to attract and activate DNMT1, which enhances methylation on nearby DNA segments (Figure 6A). The associated HP1 and DNMT1 at specific gene loci then may attract G9A H3K9 methyltransferase thereby creating a mutually reinforcing feedback loop to repress gene expression [115]. UHRF1 (ubiquitin-like, containing PHD and RING finger domains 1), which is a multi-functional, multi-domain, “hub” protein, has an affinity for both the H3K9me3 mark through its tandem Tudor domains and for hemi-methylated DNA through its SRA (Set and Ring associated) domain. UHRF1 is associated with PCNA during DNA replication and helps to recruit DNMT1 to the hemimethylated sites created in the replication fork. These multiple protein:protein and protein:mark associations provide mechanisms that target DNA methylation to heterochromatic DNA regions or to euchromatic regions enriched in the H3K9 mark, thereby strengthening the repressive transcriptional state of chromatin in those areas which may be particularly important in the silencing of *TSG*s and imprinted genes (reviewed in [112,113,114]).

UHRF1 expression is frequently found to be up-regulated in a variety of human tumour types and plays a central role in the hypermethylation of tumour suppressor genes [116,117]. Experimental evidence indicates that UHRF1, through its interactions with SUV39H1 and DNA methyltransferases, contributes to epigenetic gene silencing and the oncogenic phenotype in prostate tumour cell lines. *UHRF1* knockdown reduced in vitro proliferation, clonogenicity and anchorage-independent growth and caused reactivation of numerous (17) tumour suppressor genes (Figure 6B). The *TSG* reactivations were associated with impaired binding of SUV39H1 to the promoter region of the silenced genes, along with a reduction of H3K9me3 and an increase of H3K9Ac, consistent with the transcriptional reactivation events. Promoter DNA methylation in UHRF1-depleted cells was also strongly reduced as would be expected from the loss of the DNA methyltransferase binding partners. [118]. Similar effects had been previously seen, upon siRNA knockdown of *UHRF1* expression in HeLa cells, in which *p21/CIP1/WAF1 TSG* expression was reactivated [119]. In this cell line, the repressed endogenous *p21* promoter was associated with binding of UHRF1, G9A and other repressive chromatin-modifying enzymes such as DNMT1 and HDAC1, by ChIP assays. Knockdown of *UHRF1* significantly reduced the *p21* promoter occupancy by the three chromatin-modifying enzymes. These two studies revealed the role of UHRF1 in the recruitment of H3K9-modifying enzymes and also pointed to coordinated actions of UHRF1, G9A, SUV39H1 DNMT1 and HDAC1 in the repression of individual *TSG*s expression.

MPP8 (M-Phase phosphoprotein 8) is another methyl-H3K9-binding protein that appears to be important in targeting DNMT activity to a *TSG*, affecting oncogenic phenotype. The gene is up-regulated in various carcinoma cells and stable knockdown of its expression, in immortalized human mammary epithelial cells (293T), decreased migration and invasion capacity in in vitro assays. *E-CADHERIN* expression was reactivated by the knockdown experiment [120]. MPP8 was shown to associate with H3K9 and DNA methylation enzymes (GLP, ESET and DNMT3A, respectively) (Figure 6C). The MPP8-directed DNA methylation of the *E-CADHERIN* promoter was the primary epigenetic modification responsible for the gene silencing effect and this was reversible with 5-aza-2′-deoxycytidine treatment, while for this gene, histone methylation and acetylation states appeared to play only a minor silencing role.

In the reverse direction to the previous models where the H3K9 methylation mark attracts a complex to target DNA methylation at those sites, the status of DNA methylation read by methylated CpG-binding proteins, such as UHRF1, MBD1 and MeCP2, targets the H3K9me2/3 modification to DNA methylated sites. The multifunctional UHRF1 protein binds to methylated CpG dinucleotides and recruits a variety of chromatin modifying factors, including G9A, SUV39H1 H3K9 methylation writers as well as DNMT1 and HDAC1 [117]. The methyl-CpG binding protein, MeCP2, binds to the SUV39H1-HP1 H3K9 methyltransferase-containing heterochromatin complex and it also recruits DNMT3A and DNMT1 to the complex (Figure 6D). The presence of MeCP2 resulted in increased H3K9me3 levels and repressed transcription of *H19* long non-coding RNA (see details on *H19* in the sections on non-coding RNA and CTCF interaction with p16/INK4A) which is an imprinted gene known to be DNA-methylated and associated with MeCP2 [121,122]. Similarly, MBD1 directs the SUV39H1-HP1 complex and associated histone deacetylases (1 and 2) to methylated CpG sites in DNA (Figure 6E). Transcriptional repression as a result of this association was demonstrated using engineered, non-meCpG-based, targeting of MBD1 to the promoter region of reporter vectors [123]. MBD1 and its interaction with SETDB1 methyltransferase has been suggested to be essential for the correct maintenance of H3K9 methylation in heterochromatin during DNA replication [124]. This “memory” function was shown to be mediated by a protein complex consisting of MBD1, SETDB1 and the chromatin assembly factor CAF1 that forms transiently during S-phase of the cell cycle to ensure correct placement of H3K9 methylation during replication-coupled chromatin assembly in the promoter region of the tumour suppressor p53-binding protein 2 (*p53BP2*) gene (Figure 6F), which is known to augment the DNA binding and transactivation function of TP53. Consistent with the activity of the multiprotein complex during S-phase, interference with DNA replication delayed the establishment and maintenance of H3K9 tri-methylation at *p53BP2* promoter site.

#### 2.2.1. Enhanced Expressions of the H3K9 Writers Contribute to Human Cell Oncogenesis

Overexpression of *SUV39H1* H3K9 methyltransferase interferes with senescence of primary human lung fibroblast WI-38 cells [125]. In this model cell system, senescence-associated down-regulated expression of *SUV39H1* correlated with both significantly reduced repressive H3K9 trimethylation, increased H3K9 acetylation and increased expression of RNAs from various repeated DNA sequences. Transfection of an expression plasmid, generating overexpression of *SUV39H1* in cell populations entering senescence (at population doubling 54) resulted in a significant increase in the amount of cells in the S phase, with decreases in the number of cells in the G1 phase. The authors speculated that, the down-regulation of *SUV39H1* during the programmed establishment of replicative senescence might play a role in switching chromatin structure from a conformation that promotes cell division to one that promotes cell cycle arrest. The enhanced replicative phenotype created subsequent to experimental *SUV39H1* overexpression could involve reduction of the tumour suppressor gene *p21/CDKN1A* expression since it had been shown that *SUV39H1* overexpression cooperates with the CTIP2 transcriptional co-repressor to repress *p21* gene expression in other human cells [126]. Although *p21* (a p53 inducible gene) participates in differentiation, senescence, and tumour suppression, it remains unknown if a time-limited experimental over-expression of the histone methyltransferase could lead to stable silencing of this *TSG* and thereby perpetuate an extended lifespan phenotype among any fraction of the senescent cells.

Up-regulated *G9A* methyltransferase gene expression is a common molecular feature in human lung tumour tissues and predicts poor prognosis. Stable, experimental *G9A* overexpression in weakly invasive lung cancer cells caused increased motility and metastasis in vitro and in vivo [127]. The invasive phenotype in highly expressing cells was shown to result from repressed epithelial cell adhesion molecule (*Ep-CAM*) gene expression due to DNA methylation and assembly of a repressor complex containing H3K9me2, HP1, DNMT1 and HDAC1 on the proximal promoter of this particular gene, although no change in global H3K9 methylation levels was induced by the over-expressed *G9A* methyltransferase in these cells.

#### 2.2.2. H3K9 Methyltransferase Repression Reverts Oncogenic Phenotypes to More Controlled Cell Growth

In non-small cell lung cancer cell lines, most lysine methyltransferases (*EZH2*, *G9A*, *SETDB1* and *SUV39H1*) are up-regulated [128]. Similar to the overexpression in cell lines derived from tumours, these genes were over-expressed 2 to 7-fold in experimentally immortalized and ras-transformed bronchial epithelial cells. Suppression (20% to 60% of untreated controls, using siRNA) of these enzymes in the transformed epithelial cells caused reduced cell proliferation and much less anchorage-independent colony growth in vitro. The suppression of H3K9 methyltransferases, G9A and SUV39H1, induced apoptosis, while the suppression of H3K27 methyltransferase, EZH2, caused G1 arrest.

In ovarian cancer cell lines, knockdown of *G9A* gene expression suppressed pro-metastatic cellular activities including adhesion, migration, invasion and anoikis-resistance, while *G9A* over-expression promoted these cellular properties associated with the progression and metastasis of ovarian cancer [129]. Changes in cellular behaviors were shown by in vitro trans-well invasion and hydroxymethylcellulose adhesion assays. By microarray analysis, G9A depletion down-regulated 365 genes and up-regulated 234 genes, especially affecting gene categories related to cell adhesion, migration, and protein dephosphorylation. Several tumour suppressor genes were up-regulated after G9A depletion: cadherin (*CDH1*) a cell-to-cell adhesion glycoprotein with dysfunction contributing to cancer progression by increasing proliferation, invasion, and metastasis; dual specificity phosphatases (*DUSP1*, *DUSP3* and *DUSP5*), that dephosphorylate MAP kinase and are frequently repressed in various cancer cells; *Ep-CAM* which contributes to cell-to-cell adhesion in certain cell contexts; *GADD34* (*PPP1R15A*), a growth arrest and DNA damage-induced protein; Sprouty4 (*SPRY4*), an inhibitor of the Ras/MAPK signaling cascade. The findings suggest that up-regulated *G9A* is a “prometastatic” histone methyltransferase (HMT), via its regulation of a group of specific genes; however, other than confirming by chromatin immunoprecipitation (CHIP) that G9A binds to many of the specific promoter regions, and that H3K9me2 was also present, no measurements of knockdown-specific chromatin features were made in this study in order to confirm the histone-modification-based mechanism of action in the specific *TSG* suppressions.

Genetic knockout (KO) experiments targeting DNA methyltransferases in human colon cancer cells (HCT116) reduced both DNA methylation and H3K9 methylation at the *p16* locus, resulting in re-activation of the constitutively silenced *p16/INK4A TSG* and a decreased culture growth cell rate [130]. Over about 20 cell generations following the creation of the KO cells, *p16/INK4A* silencing slowly returned and the cell growth rate improved in the cultures, in concert with histone H3K9 re-methylation, but well before DNA methylation became re-established at this locus. Thus, in this experimental perturbation of the epigenetic state, the return of silencing of a *TSG* and appearance of an enhanced growth phenotype could be achieved in cells prior to re-methylation of the DNA, suggesting a degree of independence in the writing and function of the two repressive chromatin states at this gene locus.

### 2.3. Induction of “Histone Methylation Injuries” and Passage into Cellular Memory

A maturing theme in toxicological studies related to oncogenesis is that adverse environmental conditions or exposures to toxicant chemicals may create stable chromatin changes that alter epigenetic landscape (epimutation), so that the phenotype of the next cell generation is not an accurate copy of the previous one (See Text S2 for evidence on the mechanisms of normal replication of histone PTMs in chromatin). A full understanding of causal connections between dysregulations to histone modifications and heritable expressions of oncogenic phenotypes requires consideration of (1) the mechanisms that lead to the dysregulation and (2) the molecular mechanisms that allow histone modifications to be maintained across cell generations and continue to generate activated or silenced transcriptional states, even in the absence of the original molecular initiating events. Chemically- induced perturbations in histone structure and covalent modifications may originate from direct disturbances to the activity of the modification enzymes (writers/erasers/readers) or from secondary perturbations arising during oxidative stress-induced DNA damage and repair, as was the case for several histone modification enzymes, indicated earlier, in relation to PcG proteins and epigenetic switching. In addition, altered histone imprints in chromatin may arise subsequent to events precipitated by the condition termed “replicative stress”. Replication stress is generally described as disordered structure and function at DNA replication forks that generates segments of single-stranded DNA. This state of incomplete replication may arise in several ways, including: direct disturbances to the initiation and progression of the replication machinery; conflicts between DNA replication and RNA transcription; or unbalanced replication processes due to forced replication during inappropriate metabolic conditions [131,132]. Close coupling normally exists between histone dynamics and the progression of the replication fork (Text S2), but that coupling likely becomes compromised during a cell’s response to perturbed fork progression, such as in replication fork stalling and restart pathways [133] or where global replication stress, imposed by nucleotide depletion or direct inhibition of DNA polymerase, disturbs replication past naturally occurring DNA secondary structures [134] which can lead to permanent loss of pre-existing epigenetic states (active or repressive). Under replicative stress conditions, recycling of parental histones and post-translational modifications may become disrupted at stalled replication forks. An evocative example of the wide potential for change is provided by observations of increased abundance (3-fold) of monomethylated H3K9 on old histones evicted from stalled replication forks caused by an acute inhibition of DNA replication (due to nucleotide depletion by hydroxyurea treatment) in HeLa cell experiments. The presence of excess H3K9me-modified histones under these circumstances opens up the possibility that H3K9me-marked histones would be erroneously deposited in greater abundance into chromatin at genomic sites with stalled forks, once replication stress is resolved. Deposition of the monomethylated histones into chromatin would likely be followed by a preferential conversion of the mono-methylated lysines into the repressive H3K9me3 mark (SUV39H2 di- and tri-methylates a mono-methylated substrate), predisposing any affected genes to undergo unscheduled gene silencing [135,136]. It may be possible that the interaction of DNMTs with the H3K9 methylation writers and readers may then favour the DNA segments with erroneously marked histones to be targeted by DNA methylation (see previous Section 2.2), although this idea has not been tested, directly, nor have *TSG*s yet been demonstrated to be among the targets under these circumstances.

## 3. ATP-Dependent Nucleosome Remodeling

Nucleosome remodeling factors are multi-protein complexes that harness ATP-derived energy to alter the association of nucleosomes with DNA, move nucleosomes along the DNA, or remove and exchange nucleosomes. These activities affect the higher order packaging of DNA around nucleosomes and contribute to the regulation of RNA polymerase II transcriptional elongation as well as DNA repair. There are four families of ATP-dependent nucleosome remodeling complexes: SWI/SNF, INO81/SWR1, ISWI, and NURD/CHD. Several of these complexes also contain enzymes catalyzing post-translational histone modifications (INO81/SWR-associated Tip60 acetylase; SNF-related SMARCAD1 deacetylase subunits, NURD-associated histone deacetylases; and INO80-associated deubiquitylating enzyme). In a reciprocal manner, various histone post-translational modifications influence the stability of the association between nucleosomes and the remodeling factors following their initial recruitment [137,138]. MBD subunits of NURD complexes may also target this transcriptional repressor complex to regions with methylated DNA, augmenting the effect of the repressive mark [139]. Given the growing number of defects in the epigenetic players that are found in human cancers [50] one consequence of chemical carcinogenesis processes could be the creation of gene expression changes due to mis-targeting of the reading domain proteins (for example, bromodomains of SWI/SNF that bind acetylated histones, or chromodomains and PHD fingers of NURD/CHD complexes that bind methylated histones) as a result of either altered DNA methylation patterns or histone modifications. Perturbations within these interaction pathways could lead to changes in nucleosome positioning near transcription start sites or at gene enhancers, where the induced changes could alter competition or co-operation with transcription factors. For example, modulation of SWI/SNF, ISWI or NuRD complex activities levels can change the transcriptional activity of hypoxia-inducible factor1-α which is important for regulation of cellular responses to hypoxic stress, including cell cycle arrest and apoptosis [140].

Twenty chemical inhibitors of the multi-subunit esBAF (embryonic stem cell BRG1 ATPase associated factor; a TRXG SWI/SNF remodeling complex) have recently been identified in mouse embryonic stem cells [141]. Their identification was based on a screening assay for increased gene expression of a knock-in reporter gene inserted into the *BMI1* gene, a polycomb (PRC1) component, which along with other members of the polycomb repressive complex is repressed by the active esBAF remodeling complex. Although no phenotypic consequences were reported in the screening experiments, it is interesting to consider that possibility in light of the ability of the active SWI/SNF5 remodeling complex to antagonize polycomb-mediated suppression of target genes. Indeed, it has been shown that SNF5 binds to the gene locus of the PRC2 histone methyltransferase, *EZH2*, in primary mouse embryo fibroblasts (MEFs) and experimental recombination-mediated inactivation of *SNF5* in these cells caused increased expression of *EZH2*, and *BMI1* to a lesser extent [142]. The elevated expression of *EZH2* drove epigenetic silencing of *p16/INK4A* tumour suppressor gene, a model polycomb target gene. Elevated levels of the H3K27me3 mark provided evidence of increased PRC2 complex activity at the *p16/INK4A* gene locus. Re-expression of *p16/INK4A* upon re-introduction of an active *SNF5* expression construct indicated reversibility of its silencing. *SNF5* inactivation in MEFs lead to a broad, EZH2-dependent repression of lineage-specific polycomb-regulated genes and up-regulation of a stem cell associated gene signature in human and mouse primary cells. Additionally, it had previously been shown that SWI/SNF displaces PcG silencers from binding at the *INK4A/ARF* locus, and the reactivation of *SNF5* was necessary and sufficient to restore mitotic checkpoint activation and cellular senescence in human rhabdoid tumour cell cultures [143]. Thus, this extensive body of work showed that the opposing functions of the TRXG SNF5 nucleosome remodeling complex and the polycomb repressive complexes were key regulators of epigenetic gene silencing or activation and suggests that perturbations to the balance of these activities can contribute to certain oncogenic processes controlled by tumour suppressor genes.

## 4. DNA Methylation

Abnormal DNA methylation is a consequence of deregulations of the epigenetic system that can lead to diseases and cancers. Insults (mutagenic or non-mutagenic events such as metabolic anomalies in undifferentiated or differentiated tissues) can deregulate interactions among HPTM and the DNA methylation system, inducing genome instability, and cancers associated with global DNA hypomethylation (pericentromeric instability, releasing retrotransposon and oncogene expression) and DNA hypermethylation silencing tumour suppressor gene promoters (the latter mechanism being the main focus of this review). Therefore the measurement of perturbed DNA methylation can be useful to develop additional tests to screen chemicals for potential carcinogenicity. Similar to the HPTM system, the DNA methylation system includes families of enzymes and proteins acting as writers, editors, and readers of DNA methylation marks (Table 3). These protein families are usually different from those of the HPTM system, but some read HPTM and DNA methylation marks and create HPTM themselves, thus can be considered to belong to both systems (e.g., UHRF1). This section reviews briefly the roles and functions of genes that directly contribute to the DNA methylation system either as writer, editor, or reader protein/enzymes. Then, examples of experimental manipulation of DNMTs, TET and UHRF1 protein/enzyme activities are provided to demonstrate that these are epigenetic regulators that, when perturbed, can contribute on their own to cellular transformation and/or tumourigenesis, and therefore are key players of carcinogenic mechanism of action. Table 4 provides a brief summary of those studies that appear throughout Section 4.

### 4.1. DNA Methylation Enzymes

There are three families of DNA methyltransferases (the writers: DNMT1, 2, and 3) that can transfer a methyl group from the universal methyl donor, S-adenosyl-L-methionine (SAM), to cytosine located next to guanine in the dinucleotide context 5′-CpG-3′. However, other bases and dinucleotides (CN, N = A, T, G, C) can also be methylated (by DNMT3A and 3B), particularly in embryonic stem cells or in the nervous system during early postnatal development [165]. The requirement of SAM to achieve DNA or histone methylation implies that these epigenetic modifications are sensitive to the availability of SAM. Folate and its synthetic form, folic acid (FA), are essential for the regeneration of SAM through the one carbon-metabolism pathway, and deregulating this pathway was shown to affect DNA methylation. Folic acid supplementation was recently shown to modulate the expression of *DNMTs*, DNA methylation, and to aggravate colon cancer stem cell phenotype toward colonosphere formation in vitro [144]. Dietary supplementation of folate has been effective in decreasing the prevalence of neural tube defects, but concern has emerged that at higher levels folate creates epigenetic disturbances that favour cancer development in some circumstances [166].

DNA methyltransferase-1 (DNMT1) possesses a functional region required for its interaction with proliferating cell nuclear antigen (PCNA). Thus, DNMT1, assisted by the methyl-binding protein UHRF1 (a reader) that recognizes hemimethylated sites, can bind the PCNA complex at the DNA replication fork and copy the methylation pattern of the original DNA strand to the emerging strand (although binding to PCNA is not obligatory, see UHRF1 section). Thus, DNMT1 is known as the maintenance methyltransferase. DNMT2 is an RNA methyltransferase that stabilizes tRNA [167], and in contrast to other DNMTs, DNMT2 has not been clearly implicated with carcinogenesis. DNMT3A and 3B enzymes catalyse the transfer of a methyl group to previously unmethylated DNA, and thus are referred to as de novo methyltransferases. Expressions of DNMT3s are high in embryos where they set up a methylation pattern, they decrease over cell differentiation and development, and their expressions become deregulated in cancer where for example DNMT1 and 3B work in concert to establish aberrant DNA methylation [150]. DNMT3L does not have methyltransferase activity on its own. It is a regulatory factor that can bind to unmethylated H3K4 and partner with DNMT3A/3B to induce DNA methylation and can also assist DNMT1 in maintaining the DNA methylation pattern [168].

DNMT3A and 3B possess different functional domains (PHD, ADD, and PWWP domain) for protein-protein interactions, for example the PHD domain recognizes H3K4me3 whereas ADD affinity for H3K4 and H3K9 is dependent on their methylation states [169]. Thus, DNMT activities are guided by epigenetic features that can be refractory to DNA methylation (e.g., H2A.Z, CTCF, and H3K4me3), or that promote DNA methylation (e.g., unmethylated H3K4, heterochromatin protein-1 (HP-1) and H3K9me3 in pericentric heterochromatin, H3K9me3 in early embryonic gene promoters). While coordinated changes in such epigenetic features occur in the genome in primordial germ cells (PGC), embryonic stem cells (ESC), and differentiated somatic cells, genome-wide DNA methylation and chromatin modification patterns are frequently deregulated early during carcinogenesis [170,171].

In mice, deletions of *Dnmt1*, *3a*, *3b*, *3l*, induce embryonic/fetal or pubertal lethalities (reviewed in [172,173]). In human, *DNMT3B* mutation causes the ICF (immunodeficiency, centromeric region instability, and facial anomalies) syndrome. Mutations, or increased expressions of *DNMT1*, *3A*, or *3B*, are found in numerous cancers (colorectal, pancreas, liver, bladder, breast, acute myeloid leukaemia) (reviewed in Timp and Feinberg [174]). The hematopoietic system provides an example to demonstrate by gene manipulation the importance of DNMTs in carcinogenesis. The pathway of hematopoietic differentiation is highly regulated by DNA methylation. It was shown that reduction in *Dnmt1* expression in mice carrying a hypomorphic *Dnmt1* allele induced DNA hypomethylation within centromeric or pericentric regions, genome instability, and development of T cell lymphomas [175,176]. *DNMT3A* is also frequently mutated in hematopoietic malignancies. Approximately 50% of the *DNMT3A* mutations affect Arg882 located at the catalytic domain. Using retroviral transduction and bone marrow transplantation experiments, Xu et al. [177] demonstrated that this mutation alone drives the development of chronic myelomonocytic leukemia in mice by inducing minimal changes in DNA methylation of CpG islands together with hypo and hypermethylation of gene bodies of a number of key hematopoietic genes. Additional indication of direct implication of DNA methylation comes from the drug 5-aza-2′-deoxycytidine which sequesters DNMTs, prevents further DNA methylation, and induces re-expression of silenced tumour suppressor genes. This drug was approved by the US Food and Drug Administration a decade ago to treat myelodysplastic syndromes, given that it improves hematopoiesis, delays disease progression, and improves survival and quality of life for a subset of patients [178].

### 4.2. Active/Passive Demethylation Systems (Erasers/Editors)

Links between DNA methylation and cancer were initially suggested by the discovery of hypomethylated genes in 1979 (historical timeline in Baylin and Jones [179]). Demethylation of DNA can occur through “passive” mechanisms such as a reduction in recognition of 5mC or 5-hydroxymethylcytosine (5hmC) marks and/or subsequent omission of the maintenance of the DNA methylation marks during DNA replication. The spontaneous deamination of 5mC leading to C to T transition mutation also contributes to passive demethylation. It was suggested that one-third of all patho-physiological mutations occur at methylated CpG sites, examples of this type of mutation and associated diseases were found in *DNMT3A* (acute myeloid leukaemia), *DNMT1* (colorectal cancer), *DNMT3B* (ICF), *p53* (colorectal, breast and ovarian cancers), and *BRCA1* (breast and ovarian cancers) (reviewed in [173]).

Active demethylation is enzymatically driven and demethylation of 5mC occurs through a successive series of oxidation reactions conducted by the Ten Eleven Translocation (TET) family of enzymes (reviewed in [180,181]). This enzyme family was named after the translocation *t*(10;11)(q22;q23) present in acute myeloid leukemia (AML) patients. While *TET1* mutation is responsible for AML, the other TET2 and TET3 enzymes as well as TET enzyme cofactors (isocitrate dehydrogenases, IDH-1, and -2) were found mutated in several types of cancer and contribute to DNA methylation deregulation. Through the TET enzyme active demethylation pathway the methyl group on 5mC is oxidized to 5hmC which is further oxidized to 5-formylcytosine (5fC) and then to 5-carboxylcytosine (5caC). The removal of 5hmC occurs through deaminidation to 5-hydroxymethyluracil which is repaired by the base excision repair (BER) pathway, the removal of 5fC and 5caC is mediated by BER and thymine-DNA glycosylase pathways.

TET proteins belong to the family of α-ketoglutarate (α-kg) dioxygenases that require α-kg, Fe^2+^, and ascorbate as cofactors (reviewed in [182]). The extent by which TET activity can be modified by changes in metabolism, mitochondrial dysfunction, Fe^2+^ metabolism, ascorbate depletion and production of endogenous α-kg inhibitors, are being investigated (reviewed in [180]). Citrate, from the mitochondrial Kreb cycle, is transformed into isocitrate, and α-kg is produced by oxidative decarboxylation of isocitrate by isocitrate dehydrogenases (IDH). *IDH* mutations (frequently mutated in cancers) can lead to the production of 2-hydroxyglutarate (2-HG), fumarate, and succinate, as competitive inhibitors of TET enzymes. *IDH* mutations can drive cellular transformation in vitro, but are not sufficient to induce leukemogenesis, nevertheless inhibitors of 2-HG or of mutant *IDH* expression can reverse pathogenic features. Vitamin C enhances the catalytic activity of TET1, TET2, and histone demethylases and generates 5hmC marks necessary for to mesenchymal-to-epithelial transition in reprogramming of somatic cells to iPSCs (reviewed in [180]). Nickel, a divalent metal inducing epigenetic disruption, was shown to inhibit dioxygenase enzymes by displacing Fe^2+^ from the active site [183]. Collectively, these observations support the idea that metabolic events other than mutations can modulate the DNA methylation system and drive adverse oncogenic effects.

Experimental manipulations of the TET enzymes have demonstrated their importance in embryonic development, stem cell differentiation, and carcinogenesis. *Tet1* and *Tet2* KO mice are fertile and viable, double KO of *Tet1* plus *Tet2* mice is partially lethal producing some viable mice with perinatal lethality, while mice with *Tet3* KO develop to term and die at birth. *Tet1/2/3* triple-KO mouse embryonic stem cells cannot support embryonic development (reviewed in [184]). Ko et al. [180] have recently reviewed the numerous in vivo experiments in mice and in human stem cells where expression and exon and intron modifications of TET2 were tested. They concluded that TET2 deficiency skewed differentiation of hematopoietic stem/progenitor cells, that TET2 can function as a driver in the pathogenesis of myeloid malignancies, but given the disease latency (one year) and low penetrance (20–30%), as in the human disease, cooperation with additional genetic lesions is necessary to cause the full-blown disease. *Tet1* KO mice develop hematopoietic malignancies of B cell origin that carry a spectrum of mutated genes reminiscent of that found in human follicular lymphoma and diffuse large B cell lymphoma [185]. Also, transcriptional down-regulation rather than mutation of *TET* family enzymes or *IDH* result in the depletion of 5hmC observed and implicated in the oncogenic phenotype in many cancers, including melanoma, breast, lung, and prostate cancers [186,187,188]. For a summary of *TET* and *IDH* mutations see [182]. These experiments regulating expression of the *Tet* gene family in mice demonstrate some redundancy between Tet1 and Tet2, specificity for Tet3 and potential to rescue some Tet1/2 activities, and critical importance of Tet enzymes during embryonic cell differentiation for proper gene regulation (reviewed in [184]).

TET enzymes contribute to limiting areas of methylation in the genome. In differentiated human kidney cells (HEK293T), *TET1* knock-down decreased genomic 5hmC content, reduced the relative enrichment of 5hmC at the boundaries of CpG islands proximal to a number of genes and permitted spreading of 5mC into those hypomethylated islands, reducing expression of some of the genes that were studied [189]. TET1 5mC dioxygenase activity, apparently, works as a maintenance demethylase in these circumstances, preventing de novo spreading from methylated CpG island edges.

Beyond TET-induced DNA demethylation, their enzymatic products that are the oxidised cytosine variants are being investigated for specific biological roles. RNA polymerase II elongation complex is not affected by 5mC, but is retarded by the presence of 5fC and 5caC (but less by 5hmC) through hydrogen-bonding interactions in the conserved epi-DNA recognition loop, thus oxidized cytosine variants can affect transcription rate and expression of some genes [190]. Investigating three mouse cell types (embryonic stem cells, neuronal progenitor cells, and adult brain tissues), Spruijt et al. [191] found that 5mC, 5hmC, 5fC, and 5caC recruit distinct sets of proteins, and that only some proteins are the same across tissues. They report that this contrasts with proteins interacting with histone H3 which are constant between different cell types. They found that DNA repair proteins bind 5hmC, 5fC, and 5caC, but not 5mC, and additional proteins not related to DNA damage bind specifically to these C variants. For example, the winged-helix (WH)-domain-containing proteins (Rfx5, Rfxap, Rfxank) and distal-less homeobox proteins (Dlx-1, -5, and -6) bound specifically to 5mC.

The discovery of these additional cytosine modifications (5hmC, 5fC, 5caC) provided a better understanding of the sequence of events leading to the demethylation process. However, they also add complexity to epigenetics given that cytosine variants also carry biological roles mediated by reader proteins (next section) that specifically bind 5mC or 5hmC, or by proteins that are repulsed by them (e.g., CxxC-domain proteins). Some have proposed that methyl binding proteins can protect against TET activity and prevent removal of 5mC and 5hmC marks [192].

### 4.3. Methyl Binding Proteins (Readers)

Cytosine itself, the methylation marks laid down by DNMTs and further edited by the TET enzymes, offer cytosine variants as potential binding sites to methyl binding protein families [147,192], including the methyl-CpG- binding domain (MBD) proteins, zinc finger proteins such as the KAISO protein family, the SET and RING finger-associated (SRA) domain proteins including UHRF proteins [193] (see Table 3). Other families are repulsed by modified C, for example, the CxxC-domain containing proteins (e.g., CxxC5, CxxC1, KDM2A/B, MLL1, TET family) bind only to unmethylated cytosines where they carry out specific roles; TET enzymes and KDM2A maintain CpG islands free of DNA methylation and H3K36me2 marks, and CxxC1 (known as CFP1) attract the histone methyltransferases to lay down the H3K4me3 mark [180,191]. Through such mechanisms, “reader” proteins can directly, or indirectly with effector protein partners, further change DNA methylation, HPTM patterns, and/or chromatin structures to produce biological effects (e.g., DNA repair, gene expression or silencing, etc.). The number of DNA reader proteins is expanding with the increasing knowledge about protein-DNA binding mechanisms.

Eleven proteins contain the methyl-CpG-binding domain (MBD), that may or may not be functional, and these are referred to as MBD proteins or methyl binding proteins. Li et al. [147] divided MBD proteins in three groups based on their functions and sequence homology. The “MeCP2_MBD” group is the largest including 7 members (MeCP2, and MBD1 to MBD6) that possess the canonical MBD domain, but only 4 members have high specificity for 5mC (MeCP2, MBD1, MBD2, and MBD4). Given that *Mecp2*-, *Mbd1*-, and *Mbd2*-KO mice are not lethal and that all three MBD bind 5mC, they are suspected to have specific but also complementary roles. MeCP2, MBD2 and MBD3 can also bind 5hmC and MBD3 is the only one that can bind all three forms of cytosine (C, 5mC, and 5hmC) (reviewed in Du et al. [192]). Despite the fact that MBD5 and 6 have the MBD, it is not functional and these proteins bind C but do not bind 5mC.

#### 4.3.1. MeCP2

Mutation of *MeCP2* in human causes the X-linked RETT syndrome, which is lethal in boys [194] and reported as one of the most common causes of neurological impairments in girls (reviewed in [165]). This discovery contributed to the emergence of epigenetic neurological research, which for example highlighted developmental brain specific methylation nucleotide sequences involving mCpG, but also mCpA, mCpC, and mCpT, and thus potential additional roles of MeCP2 due to its affinity for 5mC and 5hmC but also for methylated CpA (reviewed in [165]). KO-experiments of *Mecp2*, or *Mbd1* in mice induces neurological disorders, and *Mbd1* KO mice showed learning deficit, and decreased hippocampal neurogenesis (reviewed in [195]). MeCP2 is a nuclear protein with multifunctions dependent on its cellular abundance and post-translational modifications; it was found to repress transcription through the recruitment of chromatin remodeling complexes including histone deacetylase, it is a genomic architectural factor, it regulates mRNA splicing, it was also suggested to act as transcriptional activator (reviewed in [196,197]). Recent data indicate that MeCP2 also localizes at the centrosome and at the mitotic spindles in dividing and postmitotic cells and was suggested to contribute to genome stability [197]. Knock-down of *MeCP2* in human prostate cell lines decreased cell proliferation, reduced abundance of nuclear envelope proteins (nuclear lamin A, C, B1, and lamin B receptor) and altered nuclear envelope integrity, likely disturbing nuclear chromatin organization including links between the nuclear membrane and peripheral nuclear heterochromatin [161].

MeCP2 has demonstrated roles in oncogenesis. Experiments using siRNA, or plasmids, were shown to reduce or increase the expression of *MeCP2* and to increase or decrease the proliferation of hepatocarcinoma and prostate cancer cell lines [145,146]. In the hepatocarcinoma cell line these phenomena were induced by MeCP2-modulation of the sonic hedgehog (Shh) pathway that regulates cell fate, morphogenesis, mesenchymal-epithelial cell interactions [145]. In contrast, the plasmid-induced *MeCP2* expression in androgen-dependent LNCaP prostate cancer cells induced androgen-independent growth through the c-MYC oncoprotein pathway [146].

#### 4.3.2. MBD1-6

The role of MBD1 in recruiting proteins and non-coding RNA to maintain the repressive mark H3K9me3, has been recently reviewed [147]. MBD1 is unique among the MBD family by having a CxxC3 domain and thus can also bind unmethylated DNA. Reading methylated or unmethylated DNA marks, it contributes to HPTM acting as transcriptional repressor in gene promoters or heterochromatin during DNA replication. It contributes to the formation of the H3K9me3 heterochromatin mark through the formation of the methyltransferase complex MCAF1/MBD1/SETDB1 (note that SETDB1 is a MBD protein that does not bind 5mC), and can recruit the HDAC3 complex to induce chromatin condensation. MBD1 expression is tumourigenic in pancreatic cancer cells but is suspected of a tumour suppressor role in other cancers. Reducing its expression in pancreatic cancer cells using small interfering RNA up-regulates the expression of many tumour suppressor genes, inhibits cell growth and invasion, and induces apoptosis. MBD1 also forms the deacetylase complex Twist-MBD1-SIRT1, which reduces *E-CADHERIN* transcription activity and increases epithelial-to mesenchymal transition. These effects following reduction of *MBD1* expression might be cancer-type specific and were not observed in prostate cancer cells. While the previous experiments suggest a tumourigenic role, *MBD1* is also found mutated in colon and lung cancer cell lines, supporting a tumour suppressor role (reviewed in [147]). Both MBD2 and MBD3 stabilize the nucleosome remodeling deacetylase complex NuRD/Mi-2, and thus mainly contribute to transcriptional repression. The relative abundance of MBD2 (low in embryonic stem cells (ESC)) and MBD3 (present in ESC) was suggested to determine which MBD will partner with the complex [191]. Others suggested that MBD2/NuRD or MBD3/NuRD is targeted to different epigenetic marks, and given the affinity of MBD3 for 5hmC that MBD3/NuRD complex regulates expression of 5hmC-marked genes in ESC [163]. *MBD2* knockdown experiments in human prostate cancer cells have shown that it is also necessary for the maintenance and spreading of hypermethylation from “seeds” of methylation at gene promoters with low expression, such as polycomb repressed genes, probably due to an ability to associate with DNMT1 and DNMT3A [198]. *Mbd3*-KO is embryo-lethal, but not Mbd2-KO mice [192]. MBD4 possess a glycosylase domain and recognize the symmetrical mismatch TG:meCG (or UG:meCG) and its role is more to participate in the DNA repair than transcriptional repression. *Mbd4*-KO is not lethal, and will increase intestinal tumour formation only in *Apc* mutated mice (reviewed in [192]). MBD5 and MBD6 localize to pericentric heterochromatin, they do not bind methylated DNA, and both interact with the human polycomb deubiquitinase complex acting on H2AK119. Both are expressed in the testes while MBD5 is also expressed in the brain and oocytes (reviewed in [192]).

#### 4.3.3. SETDB and BAZ2

The second group of MBD proteins possesses H3K9 methyltransferase activities, including SETDB1 and SETDB2 (SuVar3-9, enhancer of Zeste, Thritorax domain, bifurcated 1/2). SETDB do not bind 5mC, but locate to 5mC sites as a partner with MBD1 that binds 5mC (reviewed in [192]). *SETDB1* was recently found to be the most significantly up-regulated epigenetic regulator in human hepatocellular carcinomas, which also correlated with disease progression, cancer aggressiveness and poorer prognosis [160]. Knockdown experiments of *SETDB1* reduced hepatocellular carcinoma cell proliferation in vitro and suppressed orthotopic tumorigenicity in vivo [160]. There is less information about the involvement of SETDB2 in cancer; however [162] it was recently reported that SETDB2 overexpression in gastric cancer patients correlating with late stage and poor prognosis. The same group, using human gastric cancer cells in vitro, demonstrated that *SETDB2* knockdown and overexpression significantly decreased and increased cell proliferation, migration and invasion, respectively, accompanied with the expected global changes in H3K9me3 levels [162].

The third group of MBD proteins includes BAZ2A and BAZ2B (bromodomain adjacent to zinc finger 2A/2B) which can partner with DNMTs, HDAC1 and ATPase [199], and binds unmethylated DNA through the H3 and H4 N-terminal tail [200,201]. BAZ2A, also known as TIP5, is part of the nucleolar remodeling complex NoRC that induces nucleosome repositioning, histone modifications, DNA methylation, and thus silencing of ribosomal genes, centromeres and telomeres. Non-coding RNAs appear to guide the NoRC complex to its DNA targets. For example, BAZ2A has an extended MBD that binds non-coding RNA to drive the NoRC complex to the complementary DNA sequence of the ribosomal gene promoters [202]. *BAZ2A* is up-regulated in prostate cancer and is a useful marker for metastatic potential [164]. It directly interacts with EZH2 to maintain epigenetic silencing. Experiments inducing the up- and down-regulation of *BAZ2A* demonstrate that its up-regulation contributes to proliferation, viability, and metastatic potential of human prostate cancer cell lines [164].

#### 4.3.4. UHRF (Ubiquitin-Like with PHD and Ring Finger Domain Protein 1 and 2)

UHRF1 and UHRF2 recognize hemimethylated DNA (methylation on single strand) through their SET and RING finger associated domain (SRA), a mechanism unrelated to those of MBD proteins. The observations that (1) DNMT1 can bind both UHRF1 and the proliferating cell nuclear antigen (PCNA; a homotrimer that encircles DNA and recruits the DNA polymerase complex at the replication fork), and that, (2) UHRF1 recognizes hemimethylated DNA, were the foundation to explain how the DNA methylation marks are inherited across cell divisions. In this simple model, UHRF1 binds the hemimethylated DNA at the replication fork where it partners with DNMT1 to form a complex with PCNA, this ensures appropriate positioning of DNMT1 so that the methylation pattern from the existing DNA strand is copied to the new DNA strand [203]. UHRF1 is among the few methyl binding proteins with binding affinity to both 5hmC and 5mC [193]. Interestingly, DNMT1 has reduced catalytic activity in the presence of 5hmC-template, and it was suggested that this may reduce DNA methylation of the nascent strand leading to passive demethylation through successive DNA replication (reviewed in [204]).

The increasing knowledge about the mechanisms of interactions among UHRF1, DNMT1, and DNA reveal additional functions for UHRF1 and DNMT1 supporting mechanisms to direct DNMT1 to other sites than the replication fork. Qin et al. [205] indicate that UHRF1 acts as a reader and a writer of histone marks by having E3-ubiquitin ligase activity and ability to recognize both H3K9me3 (a heterochromatin abundant HPTM) and unmethylated H3R2 histone marks. They suggest that UHRF1 binds all three hemimethylated DNA, H3K9me3, and H3R2, and that the interaction with H3R2 is required for UHRF1 to ubiquitinate H3K18. DNMT1 has ubiquitin interacting motif (UIM) and can bind ubiquitinated (ub) H3K18 and H2AK119ub, and thus PCNA interaction is no longer essential for DNMT1 chromatin binding. Moreover, Qin et al. [205] suggest that DNMT1 UIM binding to H2AK119ub might direct DNMT1 to other sites and to DNA replication independent activities and such interactions can possibly be subjected to regulation by ubiquitin-specific proteases. Perhaps these epigenetic interactions (interactome) will assist in understanding cell cycle dependent UHRFs activities [206]. Indeed, throughout the cell cycle both UHRF1 and UHRF2 proteins bind DNA and H3K9me2/3 and localize to pericentric heterochromatin regions, while UHRF1 interacts with DNMT1 only during the S phase of the cell cycle. UHRF2 interacts with DNMT1 but not in S phase of the cycle [206].

Less is known about UHRF2 than UHRF1, while both proteins bind H3K9me2/3, hemimethylated DNA, and interact with DNMT1, DNMT3A and DNMT3B, and histone methyltransferase (HMT) G9A, they appear to have distinct roles. Uhrf2 cannot replace Uhrf1 in maintenance DNA methylation and does not interact with Dnmt1 in mouse embryonic stem cells [206]. Despite their high sequence and structural similarities, they differ in their relative expression level, in their ability to activate DNMT1 DNA methylation, and in their affinity for hemimethylated (on one DNA strand) or fully (on both DNA strands) methylated DNA. Indeed, while UHRF1 binds cytosine variants on one DNA strand (preferentially hemimethylated over hemihydroxymethylated DNA), UHRF2 can bind both hemi and fully methylated DNA, and moreover possesses an optimal 5hmC binding pocket to preferentially bind fully hydroxymethylated over hemihydroxymethylated DNA [207].

UHRF1 is particularly abundant in embryonic stem cells while its paralog, UHRF2, is expressed in differentiated cells, particularly in aortic smooth muscle cells [208]. While both UHRF1 and UHRF2 were shown to protect genomic stability by facilitating DNA repair (reviewed in [208]), UHRF1 dysfunction might have more oncogenic consequences. Knockdown of *UHRF1*, but not of *UHRF2*. resulted in reduced DNA methylation in cell lines (HCT116, HeLa and NIH3T3) [206] and is lethal in mice.

*UHRF1* is up-regulated in multiple types of cancers (breast, lung, colorectal, prostate, bladder, liver), promoting cell proliferation and *TSG* promoter methylation, and inhibiting apoptosis (reviewed in [152]). Involved in tumourigenesis and cancer progression, *UHRF1* is suspected of being a hepatocellular oncogene due to its overexpression having the ability to destabilize and delocalize DNMT1 [209]. Reviews of experimental manipulations of *UHRF1* expression with transfection of short-hairpin *UHRF1* RNA or *UHRF1* cDNA in colorectal, gastric, glioblastoma, or lung cancer cells [48,152,153], and transfer of subsequent gastric cell lines to athymic mice [152] suggest that by itself, UHRF1 can deregulate tumour suppressor gene expression, induce cancer cell proliferation and migration. Such role for UHRF1 might be specific to spontaneously transformed and/or cancer cell lines in which UHRF1 may only be a member of a larger deregulated pathway. In line with this interpretation, Boukhari et al. [153] demonstrated that experimental activation or blockage of CD47 (an integrin-associated protein) regulated *UHRF1* expression, and subsequently, expression or repression of *p16(INK4A)* and thus cell proliferation in human astrocytoma cells, but through a mechanism not operating in untransformed astrocytes.

#### 4.3.5. The KAISO Protein Family

KAISO and related proteins possess zinc-finger motifs responsible for DNA binding. The protein KAISO, initially found as a transcriptional repressor [210], binds to the unmethylated “*KAISO*-binding sites” (KBS; TCCTGCNA) and to methylated- but not hydroxymethylated-CGCG sequences [211]. Normally, KAISO induces transcriptional repression of genes involved in the WNT signaling pathway, such as WNT11 [212]. KAISO plays roles in carcinogenesis sometimes through unsuspected or unclear oncogenic or tumour suppressor mechanisms (reviewed in [213]). For example, knocking out *Kaiso* delayed intestinal tumour onset in the tumour-prone Apc^Min/+^ mouse [214], and *Kaiso* overexpression attenuates the lifespan of Apc^Min/+^ mouse, in this case it was demonstrated that Kaiso induces intestinal inflammation which then predisposes mice to intestinal tumourigenesis [215]. In numerous cancers, the loss of *E-CADHERIN* expression or epithelial adherens junction through destabilization of p120-CATENIN at the inner surface of the cellular membrane, are markers of cancer progression. These events influence the WNT signaling pathway that culminates with the β-CATENIN and TCF/LEF transcription factor complex binding to DNA and inducing transcriptional activation of target genes. KAISO and p120-CATENIN modulate this pathway. KAISO can induce transcriptional repression by occupying DNA targets of TCF/LEF transcription factor, or by sequestering TCF/LEF. p120 can be cytosolic and can translocate to the nucleus, it can inhibit KAISO-dependent transcriptional repression either by cytosolic p120 binding and sequestering KAISO in the cytosol, and/or nuclear p120 can bind KAISO and prevent DNA binding (reviewed in Schackmann et al. [213]).

Pierre et al. [215] summarised that although Kaiso was found as a negative regulator of the WNT signaling pathway in Xenopus embryos and mammalian cultured cells, it remains difficult to explain that KAISO overexpression increased WNT target gene expression (*Mmp7*, *Axin2*, *Cd44* and *Ephb2*) in intestines from *Kaiso* transgene mice. Understanding the context dependent activity of KAISO requires further studies [213,215].

*Kaiso* KO mice are viable and do not develop tumour [214], suggesting that the function of Kaiso may be redundant with that of other proteins. *KAISO* (synonym *ZBTB33*, located on chromosome X) has two paralogs, the zinc-finger and BTB domain-containing protein 4 (*ZBTB4*, chromosome 17, a transcriptional regulator), and *ZBTB38* on chromosome 3 (synonym: protein phosphatase 1 regulatory subunit). Both proteins can bind methylated or unmethylated DNA. Another zinc-finger protein, ZFP57 (chromosome 6), binds methylated DNA and assists in the maintenance of imprinted genes (reviewed in [193]).

### 4.4. Experimental Evidence for Perturbations to DNA Methylation Pathway Components as Contributors to Toxicity Pathways Leading to TSG Expression/Repression and Phenotypic Effects

#### 4.4.1. *DNMT* Overexpression (Phenotypic Effects/*TSG* Suppression)

The fact that CpG island hypermethylation is often repressive for *TSG* transcription, even at the earliest stages of cancer, and the fact that DNA methylation patterns are replicated across cell generations [216] have strengthened the suspicion that chemically-induced perturbations to DNA methylation patterns within CpG islands could plausibly lead to the stable repression of gene expression that confers selectable growth advantages. Indeed, stress-inducible transcription factors (AP-1, E2F, ERK) are responsible for up-regulation of *DNMT1* under certain circumstances [217] and stress-inducible up-regulation of *DNMT1* or *DNMT3B* have been shown to confer a cell growth advantage in the form of resistance to the cytotoxicity of H_2_O_2_ and other cytotoxic agents [218].

In immortalized human fetal lung fibroblast cultures engineered to express greater than three and up to nine times their basal DNMT levels, one consequence was a passage-dependent increase in the degree of de novo methylation of the genomic regions containing endogenous CpG island sequences within several *TSG*s that had constitutively low methylation levels prior to transfection of the full-length *DNMT* cDNA [219]. In similar experiments where two to three-fold overexpression of DNMT1 levels were created in non-malignant human bronchial epithelial cells, cell transformation was triggered, as indicated by anchorage-independent growth and increased invasiveness through basement membrane extract, although no effects on proliferative rates were detected [148]. Greater levels of engineered overexpression of DNMT3B, reaching the ranges typically found in human tumour cell lines, did not transform an immortalized human bronchial epithelial cell line directly, but did greatly accelerate and increase their in vitro oncogenic transformation efficiency by carcinogens [150]. The increased transformation was ascribed to the relative 3-fold increase in the number of genes epigenetically silenced by binding of DNMT3B to their promoters in the engineered over-expressing lines, increasing DNA methylation and to the coincidental enrichment of the repressive polycomb H3K27me3 mark at these sites. Silencing of the polycomb-regulated genes, many of which are known to be associated with cellular differentiation and tumour suppression, by increased expression of DNMT3B was suggested to be consistent with a process of cell dedifferentiation and re-acquisition of a stem cell phenotype.

Direct modifications of the structure of DNMT1 or interference with its interactions in the DNMT1/PCNA/UHRF complex, in several human cell lines, resulted in global DNA hypomethylation, a characteristic of almost all human tumour cells [148,149], hypomethylation of a variety of *TSG*s and oncogenes, as well as hypermethylation of several *TSG*s [148]. Disruptions to the relative expression of the UHRF1 component may be important, since engineered increases or siRNA-mediated reductions in the expression of *UHRF* increased or decreased, respectively, the invasive capacity of bladder cancer cells, via the epigenetic silencing of *KISS1* (KiSS peptin 1) [154].

#### 4.4.2. Experiments Creating Epigenetically Modified Promoters to Silence *TSG*s

Probable causal connections between (1) phenotypic changes considered to be cell transformation and (2) DNA methylation-based silencing at particular *TSG*s have begun to be addressed by direct experimentation that silences those genes. A method for targeted methylation of *TSG* promoter regions was applied to human bone marrow mesenchymal stem cells in order to suppress their transcription and gauge whether the DNA hypermethylation within a specific *TSG* would be sufficient to transform these cells [151]. The method for targeted repression was based upon transfection of methylated single-stranded oligonucleotide sequences into the cells which resulted in an efficient and specific methylation of the selected *TSG*s in the populations of transfected cells. The authors suggest that it is the transfected ssDNA that “in theory… causes formation of a hemi-methylated template during DNA replication that serves as a docking site for DNA methyltransferases (DNMTs); once recruited, DNMTs add methyl groups to the cytosines of CpG dinucleotides in the unmethylated strand” [220], which is a mechanism shared by an earlier gene targeting technology [221]. Simultaneous targeting of *HIC1* (hypermethylated in cancer 1) and *RASSF1A* (Ras association domain family 1 isoform A) resulted in down-regulated expression of both of these *TSG*s, resulting in enhanced proliferative ability in soft agar and increased cell migratory capability in a transwell invasion assay. These induced phenotypic properties were reversed upon treatment with 5-aza-dC. Drug resistance in vitro was also increased and the cells formed soft tissue sarcomas when inoculated into immunodeficient nude mice. The latter result indicated a persistent epigenetic state had been created by this method. In a separate experiment, nine other genes were simultaneously targeted that belong to the cell adhesion and contact inhibition-related Salvador-Warts-Hippo signaling pathway and with gene expressions often lost among various human tumours. In vitro phenotypic changes (increased growth in sloppy agar and cell invasion) related to the oncogeneic phenotype were produced by the targeted methylations, but tumour formation in nude mice was not observed. Altogether, the data supported the line of reasoning that positions DNA hypermethylation of *TSG*s as a causal mechanism in the multi-step development of malignancy rather than as a consequence of the malignant phenotype. Presumably, this promising approach to the manipulation of DNA methylation at specific CpG isands could be employed to test the contributions of any selected *TSG*, or combinations of *TSG*s, to transformed cell phenotypes in human cell transformation systems. However, DNA methylation is not sufficient to silence all *TSG*s. The combination of both histone modification and DNA methylation can be required as demonstrated in experiments where 5-aza-dC alone had no effect on *TSG* expression [222]. Nevertheless, methylation targets with causal transforming effects could then be considered as highly biologically relevant biomarkers for monitoring in high-throughput chemical screens.

A somewhat different approach to manipulation of targeted *TSG* promoter methylation was undertaken in murine and human cells, with the *p16/INK4A* gene promoter as a target, by inserting an engineered cis-element, designed to attract DNA methyltransferases, in the upstream region of the gene. The introduced sequence (160 bp) induced progressive de novo methylation of the promoter during subsequent in vitro culture [223]. When extended to mice, in vivo, this epigenetic engineering strategy provided direct evidence that developmental methylation of the *p16/Ink4a TSG* could cause loss of its protein expression and that the silencing was sufficient to initiate tumourigenesis at a high rate in mice. Sequence-specific targeting of epigenetic enzymes is an additional strategy that has begun to be used to assess the roles of epigenetic states in controlling transformed cell phenotypes. In one particular example of interest here, inducible expression from a stably transfected plasmid-based expression of a zinc-finger/*DNMT3* construct was designed to direct DNA methylation to the *p16* promoter region, which resulted in enhanced migration and invasion of two human gastric epithelial cell lines [224]. In addition to targeting the *p16/INK4A* gene, it should now be possible to test, in vitro, the presumptive roles of a range of *TSG* silencing events influencing animal carcinogenesis, using combinations of the above-mentioned directed silencing techniques. The result would provide direct experimental support to maps of the emerging epigenetic pathways leading toward oncogenic transformation and provide potential for the discovery of molecular biomarkers that result from specific silenced targets.

#### 4.4.3. TET1 as a Regulator of Gene Expression Networks and Oncogenic Cell Transformation

In addition to the experiments with DNMTs and their interacting factors, discussed above, several studies have been conducted in which experimental manipulations made with other DNA methylation pathway components were shown to silence *TSG*s, activate oncogene expression and/or result in phenotypic alterations that contribute to oncogenesis (in vitro/in vivo).

Poly-ADP ribose polymerase (PARP1 and 2) activity positively controls *TET1* gene expression by epigenetic mechanisms. TET1 enzyme, along with TET2 and TET3, contributes to DNA demethylation through its conversion of 5mC to 5hmC and is considered to be a tumour suppressor, acting to prevent cell proliferation and tumour metastasis in human solid tumours [225]. Its expression is frequently down-regulated during oncogenic transformation. In T cell leukemia cell lines, engineered overexpression of the poly-ADP ribose-degrading enzyme PARG, or siRNA-mediated knockdown of *PARP1* and/or *PARP2* expression and specific PARP inhibitors were used to show that PARP activity and/or sufficient poly-ADP ribose levels are necessary to maintain a transcriptionally permissive state at a *TET1* CpG island, surrounding its transcription start site [226]. The experimentally-induced loss of PARP activity increased DNA methylation and reduced the activating H3K4me3 mark resulting in a reduced TET1 protein level. A molecular consequence of these changes was shown to be reduced TET1 binding to one of its known target genes, the *HOXA9* tumour suppressor gene which codes for a transcription factor that is a positive regulator of cell fate determination and terminal differentiation, suggesting the possibility that poly(ADP-ribosyl)ation, through TET1 activity could control a network of genes due to its ability to affect both DNA and H3K4 methylation on specific genes.

The idea that *TET1* is itself epigenetically regulated and that its activation regulates a network of genes has been supported in a breast cancer cell line by experiments manipulating expression of *HMGA2* (high mobility group at-hook 2), which is an upstream repressor of *TET1* expression. When HMGA2 was depleted by siRNA in breast cancer cells, an up-regulation of *TET1* was noted [156]. That up-regulation was associated with TET1 binding to its own promoter region and demethylation of the promoter proximal to the transcription start site. TET1 induced in this way also induced *HOXA* gene cluster expression (including *HOXA9*). Similar to the *TET1* autoregulatory activation, *HOXA* expression was induced by TET1 binding to its promoter regions. Induced levels of either TET1 or HOXA9 suppressed cell invasion in vitro and xenograph tumour growth and invasion when injected into nude mice mammary fat pads. Moreover, more than 7000 genes were found to be differentially expressed by either *TET1* induction or *HOXA9* expression, with about 60% shared between the two, indicating that HOX9A is a major downstream effector of TET1 activity in these cells. Alternatively, *TET1* expression in immortalized human bronchial epithelial cells is reduced subsequent to activation of the RAS/RAF/MEK/ERK signal transduction pathway, via transfection of the cells with a constitutively active *RAS* oncogene [157], but unlike the breast cancer cells above, no effect on *HMGA2* or *HOXA9* expression was detected. Through *TET1* ectopic overexpression and inhibition of the ERK pathway, TET1 was identified as a key component in the RAS pathway driving suppressed *TSG* and *H19* RNA expression as well as oncogenic cell transformation. It is tempting to speculate that in bronchial epithelial and perhaps other cells, diverse activators of the ERK pathway, such as activated epidermal growth factor receptor (EGFR) or arsenite, can also contribute to hypermethylation of *TSG* DNA through reduced *TET1* expression.

Further support for TET proteins as regulators of oncogenic behavior was provided by engineered expression of microRNA (*miR-22*) which down-regulated expression of *TET1,2,3*, triggered epithelial-mesenchymal transition in the human MCF10 breast epithelial cell line and caused non-metastatic human MCF-7 breast cancer cell line to express metastatic properties: cell proliferation, invasion, and angiogenesis [158]. Using a variety of direct molecular biological tools, the authors showed that phenotypic changes were under the control of *miR-200* that normally inhibits epithelial-mesenchymal transition, but upon loss of *TET* expression the *miR-200* genes were down-regulated by hypermethylation. Direct knockdown of *TET* expression in fetal human colon epithelial cells with shRNA or re-expression in colon cancer cell lines were employed to determine that downmodulation of *TET1* expression is required for cancer cell growth both in vitro and in vivo. Much of the loss of growth control in these cells was attributed down-regulation of the inhibitors of the WNT-signalling pathway (*DKK3* and *DKK4*), due to hypermethylation of their promoters in the absence of TET1 expression [159].

The TET demethylase enzyme requires 2-oxoglutarate as a co-factor as well as Vitamin C for optimum activity. Adaptation of primary mouse embryo cells to growth in culture medium without Vitamin C occurs with no discernable effects on global DNA methylation, but adaptation is accompanied by reduced *TET1* expression and more than 60% global loss of 5hmC. These deficiencies could be partially restored when Vitamin C was added to the medium during adaptation [227]. During culture adaptation in the absence of Vitamin C, the 5hmC levels in several thousands of individual genes were affected, with most showing losses across gene bodies, while in contrast, only several hundred genes showed gains in 5mC at promoter regions. These results, along with concomitant changes in the expression of many of the affected genes suggested that further studies on culture media composition will be required in order to optimally reduce the scale of changes in epigenetic pathways that can occur during the culture adaptation period. Beneficial effects of Vitamin C also occur on human TET enzyme and histone demethylase activities [228], and on human cancer cell malignancy (hepatocelloular carcinoma [229]; melanoma [230]). Therefore, it is likely that further optimization of human cell culture media will be required in order to more faithfully represent in vivo cellular conditions, and for chemicals to be tested for epigenetic disturbances under the Tox21 paradigm.

## 5. CTCF and Control of Epigenetic Modifications in *TSG*s

CTCF (CCCTC-binding factor) is a master organizer of the three-dimensional chromatin architecture. Its affinity to DNA can be influenced by epigenetic marks, and with protein partners, CTCF impacts on epigenetic marks regulating gene expression of tumour suppressor genes and oncogenes in various ways. Therefore, CTCF deserves consideration in pathways to epigenetic dysfunction and cancer.

CTCF is a ubiquitously expressed protein; it includes 11 zinc-fingers [231] that are used in various combinations conferring the ability to bind at a plurality of DNA motifs. Subsets of these DNA binding sites confer to CTCF roles in nuclear spatial organization, chromosome segregation and establishment of topologically associating domains (~2000 genomic regions) [232] along the length of chromosomes through chromatin looping and chromatin insulation [233,234,235]. The interactions of two CTCF binding sites reinforced by cohesin molecules form the building blocks of thousands of chromatin loops per chromosome. Each loop insulates gene sets and brings closer distal enhancers to their promoters to facilitate gene expression [236].

Other subsets of CTCF binding sites include CpG dinucleotides that can be methylated and thus reducing CTCF binding affinity and altering the underlying chromatin function. The imprinted *IGF2/H19* locus, described in Section 5.1 and Section 6.1, provides an example of a mechanism by which DNA methylation prevents CTCF DNA binding and thus relieves a CTCF-insulating effect preventing access of an enhancer to its promoter. A similar mechanism with hypermethylation reducing CTCF affinity for its binding site explained the up-regulation of the receptor tyrosine kinase (*PDGFRA*) in gliaomas (with mutant *IDH-2-*hydroxyglutarate inhibited TET enzymes resulting in hypermethylation) [237].

CTCF by itself does not modify epigenetic marks, but it interacts with epigenetic writers to alter the expression of oncogene and tumour suppressor genes through variants of this mechanism. For example glutamic acid decarboxylase 1 (*GAD1*) acts as an oncogene in colon and liver cancer cells. Under normal condition DNA binding site is unmethylated and CTCF binds and interacts with SUZ12, which is a domain of PRC2, promoting the methylation of H3K27 and the silencing of *GAD1* expression. DNA hypermethylation, usually perceived as an indication of gene silencing, in these cases reduced the affinity of CTCF for its binding sites and prevents *GAD1* silencing [238]. CTCF is recognized to be a positive transcriptional modulator of tumour suppressor genes that are commonly silenced in cancer cells, including *p16/INK4A*, *p14/ARF*, *p53*, *pRB* and *BRCA1*, while it may have either an activating or repressive action on *c-MYC*, depending upon cell type [239].

The following three sections describe engineered experiments demonstrating the positive effects of CTCF on the expression of important tumour suppressor genes, *p16/INK4A*, *pRB*, and *p53*, and highlight other mechanisms by which CTCF create boundaries preventing spreading of epigenetics silencing marks from chromatin adjacent to promoter regions. The following section also summarize experiments about the PARylation of CTCF which is another mechanism altering its function. CTCF proteins are regulated by poly(ADP-ribosyl)ation (or PARylation) which is a post-translational modification catalysed by poly(ADP-ribose) polymerase (PARP) family [240]. PARylation of CTCF permits its sub-nuclear localization, where CTCF also stimulates PARylation by PARP1 and prevent DNMT1 activity. PARylation has multiple other targets including lysine demethylases (*KDM5B*, *KDM4D*), *H3*, *UHRF1*, *MeCP2*, *TET1*, and *EZH2* transcription [240]. Overall, this section demonstrates a number of CTCF mechanisms, by which its abundance, diversity in binding sites, and impact on epigenetic changes at the level of both histone modifications and DNA methylation, deserve consideration in pathways to epigenetic dysfunction and cancer.

### 5.1. P16/INK4A

CTCF binds the promoters of tumour suppressor genes (*RASSF1A; CDH1*, *p16/INK4A*) and in the case of *p16/INK4A*, was highly correlated with its expression in a panel of human breast cancer cell lines [241]. Proof of a mechanistic link between binding and gene expression was pursued with siRNA knockdown experiments targeting *CTCF* in several expressing cell lines. Knockdown resulted in highly reduced *p16/INK4A* mRNA abundance in addition to loss of *H19* mRNA expression from the imprinted *H19/IGF2* locus. Loss of expression of *H19* served as a positive control in these experiments and was consistent with it being frequently the target of loss-of-imprinting events in human cancers. The differentially methylated region (DMR) in the imprinted *H19* locus contains two CTCF target sites that, in many cancer cells, are both hypermethylated and incapable of binding CTCF. The absence of CTCF binding allows chromosomal looping that re-positions distal enhancers to stimulate bi-allelic expression of both maternal and paternal *IGF2* genes, simultaneously preventing enhancer access to both *H19* loci resulting in bi-allelic silencing of *H19* [242]. Loss of expression of the *p16/INK4A* mRNA in these CTCF knockdown experiments was shown to coincide with an increase in H4K20me, H3K9me3 and H3K79me1 repressive marks in the domain extending ~2kb upstream of the *H19* transcriptional start site. The gain of repressive marks in this region was coupled with a loss of H2A.Z histone variant and the H3K4me3 mark that are normally associated with active gene promoters. These siRNA-induced changes were similar to the changes measured in the cell lines with constitutively silent *p16/INK4A* expression. The authors interpreted the increase in repressive histone marks to have resulted from their spreading from the adjacent H4K20me-rich chromatin domains normally present upstream and downstream of the *p16* gene promoter domain. 5-aza-2′-deoxycytidine treatment of the *p16*-silent cell lines could restore *p16/INK4A* transcription, indicating that the CTCF DNA binding site might be methylated. The DNA methylation inhibitor also reversed the enhanced H4K20me and the diminished H3K4me3 marks, but could not restore CTCF binding to the promoter region in the silenced cell lines, which may have been related to a failure to re-establish the original chromatin boundary. This result implies that gene loci with lost or altered CTCF-bounded domain structures may be persistent changes that continue to affect histone and DNA methylation status in progeny cells. Importantly, it was also noted that under conditions in which poly(ADP-ribosyl) modification was experimentally suppressed, and in the *p16/INK4A* non-expressing cell lines, CTCF failed to form the multiprotein complex necessary to up-regulate *p16* and *RASSF1A* tumour suppressor gene expression. These experiments were interpreted to mean that, in the region upstream of the *p16/INK4A* proximal promoter, a chromatin boundary exists which normally acts to exclude or inhibit repressive chromatin modifiers. That boundary can be destabilized by loss of CTCF binding, leading to aberrant transcriptional inactivation through the loss of an active chromatin signature and gain of repressive chromatin marks. A “spreading” phenomenon of H3K9me3 and DNA hypermethylation repressive marks has also been documented for a large number of zinc-finger transcriptional proteins that are down-regulated during arsenite-induced oncogenic transformation of a prostate epithelial cell line [243]. The authors of this study invoke the possibility that an arsenite-induced disruption of CTCF function could have contributed to the spreading of repressive marks.

Given the demonstrated influences that CTCF exerts over epigenetic modifications at the *p16/INK4A TSG*, it seems likely that carcinogen-induced perturbations of CTCF abundance, as for example by H_2_O_2_-induced oxidative stress [244] or interference with CTCF activation by its poly(ADP–ribosyl)ation (see below), could be at the origins of an epigenetic toxicity pathway leading to tumour suppressor gene silencing. A pathway beginning with poly(ADP-ribose) polymerase (PARP1) perturbation is supported, but not yet directly proven, by the actions of divergent carcinogens such as benzene and silica. These carcinogens have been shown to down-regulate *PARP1* gene expression through methylation of its promoter [245,246] resulting in oncogenic transformation of human bronchial epithelial cells (16HBE) as demonstrated by loss of contact inhibition, invasiveness, disoriented organization, and overlapping growth. Asbestos and low nanomolar concentrations of monomethylarsonous acid reduce PARP1 enzyme activity and transform immortal human mesothelial cells (Met-5a) and human bladder urothelial cells (UROtsa) to anchorage independence [247,248]. In murine cells, interactions between Ctcf, Parp1 and Dnmt1 proteins are maintained in the nucleus when poly(ADP-ribosyl)ation of proteins were inhibited by experimental overexpression of poly(ADP-ribose) glycohydrolase (*Parg*); however, the resulting un-poly(ADP–ribosyl)ated complex could not localize at *Ctcf* target sites, reinforcing the idea that a balance which favours a certain level of Parp activity is essential for Ctcf to efficiently bind its DNA targets [249]. Further, it was suggested that Parp1 activity inhibits Dnmt1, within the complex, permitting the specific DNA binding sites to be maintained in an unmethylated state as long as the full complex is bound. Any chemically-induced deficiency of PARP activity would be expected to result in de-repressed DNMT1 and hypermethylation as well as compromised CTCF binding to DNA targets. These predictions were realized in a subsequent study in which depletion of poly(ADP–ribose) caused CTCF to lose it diffuse nuclear localization and accumulate at the nuclear periphery due to being blocked from nuclear entry [250]. Also a generalized genomic hypermethylation and an increased H3K9me3 repressive chromatin content were measured as a consequence of poly(ADP-ribose) depletion, reinforcing the idea that the high degree of interaction between CTCF and poly(ADP–ribosyl)ation is important for chromatin organization and DNA methylation patterns.

### 5.2. pRB

The retinoblastoma protein (*pRB*) promoter region is rich in CpG sites within its CpG islands and the promoter sequence is frequently methylated and silenced in certain human tumours [251,252,253]. In order to gauge the contribution of CTCF to the silencing events, an siRNA experiment against the *CTCF* gene was conducted in HeLa cells [254,255]. Knockdown of *CTCF* mRNA slightly reduced *pRB* gene promoter activity in a transiently transfected luciferase reporter construct. A transgene containing the entire *pRB* gene promoter in stably transfected HeLa cells produced both expressing and non-expressing populations after 100 days of culture, without continuous selection for the transgene. H3 hypoacetylation, loss of H3K4me2 and a slight increase of DNA methylation (but no increase of H3K9me1-3 or H3K27me3 repressive marks) accompanied the loss of *pRB* transgene expression in the non-expressing lines. The DNA methylation inhibitor 5-aza-2′ deoxycytidine could reverse the repression, while a histone deacetylase inhibitor could not. Using stable transfection of a *GFP* reporter gene driven by the core *pRB* promoter region it was also shown in human erythroleukemic cells that the loss of an effective recognition sequence for CTCF within the promoter caused a rapid gain of a repressive chromatin configuration. In these cells, the normal transgene was not affected over a period of 100 days, but if the *CTCF* DNA binding site sequence was altered , silencing was accelerated in time (appearing at 15 days) and increased about two-fold in frequency among transfectants [254,255]. At the 100 day time point, cells harbouring the mutated reporter gene were shown to be hypermethylated within the core promoter, relative to cells with the wild-type promoter. Thus, the loss of CTCF binding at its recognition sequence causes an increase in DNA methylation and rapid gain of a repressive chromatin configuration at the *pRB* promoter.

### 5.3. p53

Upon *CTCF* knockdown by shRNA from a stable expression vector in HeLa cells, *p53* expression was greatly reduced and its promoter was silenced, but in contrast to *p16* and *pRB* promoters, silencing was not accompanied by changes in DNA methylation [256]. Increases in H3K9me3, H4K20me3 and H3K27me3 repressive histone marks were found at the core *p53* promoter in the knockdown cells. PARP inhibitor 3-aminobenzamide also decreased *p53* expression, consistent with its known effects in reducing CTCF protein function. Although the epigenetic changes were restricted to histone modifications, the results were consistent with CTCF normally providing a shield from the accumulation of repressive chromatin marks at the *p53* promoter region, while perturbations to CTCF function permit their accumulation.

### 5.4. Genome-Scale Epigenomic Changes

Global scale relationships between CTCF binding and the prevalence of DNA methylation at specific sites have also been examined [257]. It was observed in this work that primary cells with limited proliferative potential when compared with malignancy-derived or virus immortalized cells were distinguishable solely by their CTCF binding patterns (landscapes). As one example, three out of five malignancy-derived cell lines showed decreased binding at the differentially methylated locus of the *H19* gene. Across the genomes of a total of 13 mortal and 6 immortal human cell types, at all CTCF DNA binding sites (6707) where differential DNA methylation was associated with CTCF occupancy, 69% of those variably occupied *CTCF* binding sites were characterized by immortalized cell-specific hypermethylation. At those *CTCF* binding sites that were located within gene promoters and where DNA methylation was significantly associated with CTCF occupancy, 98% (281 of 288) of the sites were hypermethylated in the immortal lines, rising over 10-fold to about 30% methylation on average in the immortal cell lines, compared to about 2% methylation in the mortal cell types. These results indicated a broad methylation-associated remodeling of the CTCF binding landscape and pointed toward a wide potential for changes to genome organization and function that could affect cellular phenotypes.

The question of how *CTCF* expression changes might affect cell phenotype has been more directly addressed in human embryonic kidney 293 cells, Hela cells and several other growth-transformed and cancer cell lines [258]. Transient or stable overexpression of *CTCF* caused a state of severe growth inhibition involving repression of both DNA replication and cell division. Growth inhibition might have been expected from the likely increase in CTCF binding and re-activated tumour-suppressor functions such as p16, pRB or p53, although molecular alterations in the chromatin associated with these genes were not investigated in this study.

A recent genetic experiment in mice has directly demonstrated a tumour suppressor role for Ctcf, by engineering reductions in Ctcf abundance as a test of its causal role in carcinogenesis [259]. The resulting *Ctcf* hemizygous (*Ctcf*^+/−^) KO mice were more likely to develop cancer in a broad range of tissues and were predisposed to develop chemically-induced cancers at a high rate. Aspects of this cancer-prone phenotype were reproduced in cultured mouse embryonic fibroblasts from these animals. The hemizygous primary cells in culture expressed half the *Ctcf* mRNA found in wild-type cells and continued to proliferate at confluence, forming foci of piled up cells at about 7 times the frequency of homozygous (*Ctcf*^+/+^) MEFs. Hypermethylation of the well-studied *Igf2/H19 Ctcf* binding site within the imprinting control region was shown in both the cultured MEFs and lung tissue from *Ctcf*^+/−^ animals. In a genome-wide scan of hemizygous lung tissue CpGs, shifts toward DNA hypermethylation were shown to occur at specific loci that are naturally divergent for methylation levels among individual mice, especially in gene exons, introns and intergenic regions, but infrequently within promoter regions, indicating a wide-spread destabilization of the regulation of DNA methylation in the hemizygous state. The authors also showed that *CTCF* is frequently hemizygous in human tumours, and interestingly, among the human tumours investigated, the *H19* locus was frequently found to be hypermethylated, as was the case in the hemizygous mice.

Together, these studies suggest that reductions in the function or amount of CTCF leads strongly to epigenetic instability (at the level of both histone modifications and DNA methylation) in human cells and that the instabilities can precede and accelerate cancer progression in rodent cells. Gene knockdown experiments in primary human cell models should be conducted in order to ascertain the extent to which phenotypic manifestations of *TSG* silencing and oncogenic transformation can be induced by CTFC perturbations.

## 6. Non-Coding RNA: Roles in Both Oncogenic and Tumour Suppressive Pathways

Three quarters of the sequences in the human genome give rise to RNA transcripts [260], although only a small fraction of the genome is protein-encoding sequence, with over 21,000 long intergenic non coding (lincRNAs) and transcripts of uncertain coding potential (TUCP) listed on the ENCODE genome browser site (Available online: http://genome.ucsc.edu/ENCODE). Although the biological functions of the vast majority remain unknown, a growing number of the many thousands of long non-coding RNAs (lncRNAs; >200 bp) are recognized to be participants in structural and regulatory mechanisms affecting gene transcription by RNA polymerase II. In addition they are also known to act in diverse cellular contexts such as post-transcriptional regulation, multiprotein complex functions, small RNA processing and DNA recombination [261]. In their roles associated with RNA transcription, lncRNAs may directly contact the DNA template in order to fine-tune gene expression regulation at the level of transcriptional control. These RNAs play structural roles in *cis*- (near their site of transcription) or *trans*- (away from their site of transcription) where they act as guides or scaffolds that direct, localize and help assemble chromatin modifying complexes, such as the polycomb repressive complexes, at specific loci. Alternatively, they may act to sequester transcription and chromatin factors away from their genomic targets [262,263,264].

Examples of lncRNAs (*H19*, *HOTAIR*, *ANRIL*, *MIR31HG*), that recruit chromatin modifying activities and have been shown to have roles in cellular growth control and carcinogenesis, or are known to regulate the expression of tumour-suppressor genes, are presented below.

### 6.1. H19

The *H19*, paternally-imprinted gene is transcribed and processed to produce a 2.3 kb RNA species from a site within the *IGF2/H19/CDKN1C* imprinting cluster. Several CpG islands form two imprinting control regions (ICRs) and a proximal promoter upstream of the *H19* transcriptional start site. Transcription is controlled through an enhancer competition mechanism (methylated during male gametogenesis and silenced/untranscribed on the paternal chromosome; unmethylated and actively transcribed on the maternal chromosome), making this gene functionally haploid. In their methylated state, the paternal differentially methylated regions (DMRs/ICRs) cannot bind the chromatin insulator CTCF (CCCTC-binding factor), which permits a long-range DNA loop to form, whereby the upstream *IGF2* locus successfully competes for the factors at the shared enhancer segment downstream from *H19*, suppressing paternal *H19* transcription [265]. The DMRs within the locus are sensitive to the environmental conditions experienced by the egg during ovulation and developing embryo through to blastocyst implantation [266].

*H19* functions in embryonic growth control since targeted deletion of the *H19* transcriptional unit creates an overgrowth phenotype during mouse development. *H19* non-coding RNA acts as a transcriptional suppressor not only for the *Igf2* gene (in *cis*) but also in *trans* on 9 other imprinted genes belonging to a 17-member network controlling growth and survival of mouse embryos [267]. Further work has demonstrated how *H19* RNA depresses expression, in *trans*, of 8 genes belonging to that imprinted gene network. For five members of the network, *H19* forms a complex with methyl-CpG-binding domain protein 1 (MBD1)at the gene promoters, where the complex recruits H3 lysine methyl transferases in order to add the repressive H3K9me3 mark [268]. Such RNA-based gene targeting may permit histone modifying enzymes that have putative RNA-binding domains but no sequence-specific DNA-binding activity, to target specific gene loci. While several in vivo *H19* gene deletion experiments have indicated a tumour suppressor function, possibly during the early formation of tumours (reviewed in [267]), increases of *H19* RNA are thought to contribute to metastatic growth in the later stages of tumourigenesis. The complexity of the effects on cellular growth control by RNA products that originate from the *H19* locus is probably not yet fully understood, due to alternate transcriptional start sites and alternate *H19* splice isoforms that generate many RNA species. In addition, there are probably cell context-dependent roles of those RNA products [269].

### 6.2. HOTAIR

The 2.2 kb processed *HOX* antisense intergenic RNA (*HOTAIR*) is expressed from within the developmentally important *HOXC* locus on chromosome 12. *HOTAIR* was originally understood to function, in *trans*, to silence the *HOXD* locus genes present on chromosome 2 in humans via recruitment of several gene silencing complexes. *HOX* genes encode a family of transcription factors defined by the presence of a homeodomain and act to establish the anterior–posterior body axis during development. The PRC2 (polycomb repressive complex 2) and H3K4 demethylase LSD1 in the CoREST/REST repressor complex interact with *HOTAIR*. siRNA-mediated knockdown of this non coding RNA leads to dramatic activation of expression of the *HOXD* developmental genes in human fibroblasts, spanning a 40 kb region [270]. At specific *HOXD* gene promoters, *HOTAIR* maintains the K3K27 methyltransferase functionality of PRC2 by interacting with the RNA binding domain of SUZ12, a core PRC2 component. Thus the depletion of *HOTAIR* by siRNA reduced the occupancy of SUZ12, the abundance of the repressive H3K27me3 mark, and induced the expression of *HOXD* genes.

In a subsequent study, LSD1 a histone lysine demethylase specific for removing the activating H3K4me3 mark, was shown to interact with the 3′ end of *HOTAIR*, while the PRC2 EZH2 methyltransferase bound to the 5′ region. Knockdown of this non coding RNA produced decreases in H2K27me3 marks and increases in H3K4me2 marks at *HOXD* loci that correlated with the amount of loss of EZH2 methyltransferase and LSD1 demethylase, respectively [271]. These studies support the idea that *HOTAIR* RNA acts as a bridge to coordinate and guide the machinery required for gene silencing (PRC2 and LSD1- chromatin modification complexes) to the promoters of its target genes.

Experimental over-expression of *HOTAIR* causes aberrant silencing of tumour suppressor genes, resulting in oncogenically transformed phenotypes. For example, overexpression and knockdown experiments showed that *HOTAIR* RNA expression mediates the invasive phenotype of human breast epithelial cells. Upon *HOTAIR* overexpression, an induced localization of the H3K27me3 repressive mark and the PRC2 subunits SUZ12 and EZH2 on over 800 genes was demonstrated, considerably extending the likely functionality of *HOTAIR* beyond the *HOXD* locus. The majority of these genes were involved in pathways related to cell–cell signaling and development [272]. *HOTAIR* expression in breast cancer cells seems to re-impose the polycomb binding profile of an embryonic fibroblast thereby silencing metastasis suppressor genes and favouring a gene expression program that is conducive to cell motility and matrix invasion. In addition to experiments in breast cancer cells, gene expression knockdown experiments show that *HOTAIR* is instrumental in promoting metastasis in a number of types of cancer, including, hepatocellular carcinomas [273], gastrointestinal stromal tumours [274], pancreatic carcinomas [275], and oesophageal squamous cell carcinoma [276]. It therefore should be of concern that *HOTAIR* expression is induced by nanomolar concentrations of xenoestrogens/xenobiotics (bisphenol-A, BPA or diethylstibestrol, DES) in human breast cancer cells in vitro and in rat mammary glands in vivo [277]. Estrogen receptor and estrogen receptor co-regulators such as MLL1 and MLL3 (TRXG H3K4 methyl transferases), CBP and p300 (histone acetyl transferases) associated with the *HOTAIR* promoter in presence of BPA and DES. This association increased the activating H3K4me3 and H3K4ac marks, leading to *HOTAIR* RNA expression. It remains to be demonstrated how many of the known and probable tumour suppressor genes that are silenced by experimental *HOTAIR* overexpression can be repressed by *HOTAIR*-inducing levels of estrogenic compounds in primary or immortalized human breast epithelial cells.

### 6.3. ANRIL

*ANRIL* (antisense non-coding RNA in the *INK4* locus) is a 3.8 kb processed, antisense lncRNA, transcribed from the 42 kb *INK4b*–*ARF*–*INK4A* tumour suppressor locus. *ANRIL* RNA functions, in *cis*, to silence the *p15/INK4B* tumour suppressor gene by binding to SUZ12 (suppressor of zeste 12 homologue), which is the PRC2 nucleosome-binding component [278]. *ANRIL* expression was shown to be necessary for targeting the PRC2 complex to the p15/INK4B locus and deposition of the H3K27me3 repressive mark. Experimental down-regulation of either *SUZ12* or *ANRIL* expression, by RNA interference in human WI-38 diploid human primary cells, resulted in up-regulation of *p15/INK4B* mRNA and to some extent *p16/INK4A* mRNA. The experimentally-increased expressions were associated with the premature senescence of the treated cells. In a previous study, *ANRIL* overexpression in human prostate tumour cells was shown to repress *INK4b/INK4A* mRNAs expression, and this repression was mediated through the direct binding of *ANRIL* to the PRC1 component, chromobox homologue 7 (CBX7). This binding complex, in turn, binds to the PRC2 H3K27me3 repressive mark leading to attachment of the repressive histone ubiquitination mark by the active PRC1 complex [279]. It was also shown that anti-sense targeting of *ANRIL* expression by retroviral vectors in human IMR90 diploid cells caused increases in the expression of p16/INK4A and p15/INK4B mRNA and protein, which correlated with reduction of H3K27me3, and EZH2 at the transcriptional start site of the *p16/INK4A* gene. These molecular alterations in PRC2/1 occupancy and activity were associated with a culture passage-related reduction in cell population doublings (earlier senescence) in the transfected, *ANRIL*-knockdown cultures.

Taken together, these studies revealed a mechanism in which the lncRNA *ANRIL* is involved in chromatin reorganization via the recruitment of the two PRC complexes resulting in the epigenetic repression of genes in the *INK4B-ARF-INK4A* locus. The disruptions to *p16* and *p15* expression by this mechanism were shown to be drivers of the extended lifespan phenotype associated with human oncogenesis.

### 6.4. MIR31HG

Oncogene induced senescence (OIS) by conditional expression of constitutively activated *B-RAF* oncogene, in diploid foreskin fibroblasts and in immortalized human diploid fibroblasts, reduced the nuclear abundance of this 2.2 kb polyadenylated RNA species by its export to the cytoplasm [280]. Knockdown *MIR31HG* expression, without *B-RAF* induction, reduced cell growth in a manner similar to *B-RAF* induction, with significant increases in p16/INK4A gene expression (mRNA and protein), resulting in repression of the E2F transcription factors and their pro-proliferative target genes. The senescent phenotype induced by *MIR31HG* RNA knockdown could be prevented by depletion of p16/INK4A and could be reverted by *MIR31HG* overexpression, indicating that senescence was mediated by p16/INK4A in this experimental situation. In contrast, up-regulation of *p16/INK4A* by *B-RAF* induction was diminished by *MIR31HG* overexpression, but there was no effect on cell growth, suggesting that the molecular mechanisms controlling B-RAF induced senescence are probably more complex than just *p16/INK4A* up-regulation. The up-regulation of *p16/INK4A* by *MIR31HG* knockdown was shown to be a consequence of reduced presence of polycomb repressive complexes and the H3K27me3 mark at the *p15/INK4A* locus. Importantly, it was shown that *MIR31HG* RNA could interact with polycomb complexes and that *MIR31HG* interaction with the *p16/INK4A* promoter region was diminished when polycomb components were depleted by RNAi knockdown. Although it is clear that this lncRNA species is likely to be an important component of *p16/INK4A* transcriptional regulation, it will be important to ascertain whether or not persistent polycomb-mediated repression of *p16/INK4A* expression and consequent reversion of the senescent phenotype can occur as a result of temporary up-regulations of *MIR31HG* RNA.

## 7. Experimental Evidence from In Vitro Cell Transformation Models Supporting the Roles of Chemically-Induced Epigenetic Perturbations as key Steps in Developing Cancer-Related Adverse Phenotypes

Cancer is a well-studied apical toxicological endpoint, associated with ubiquitous epigenetic alterations in tumour cells. There are many examples of dysregulated pathways of epigenetic control that are thought to drive phenotypic changes in tumour cells (reviewed extensively by [281,282,283]). A central challenge for the in vitro TT21C approach to epigenotoxic carcinogenesis testing in human cells is to identify and functionally validate steps in epigenetic pathways that would lead to tumour-related phenotypic changes. The earlier sections of this review have highlighted much of the available evidence for causal participation of epigenetic events in several human cell transformation models.

Cell transformation systems are likely to provide the most relevant in vitro systems in which to begin to characterize functional impacts of chemically-induced perturbations within the epigenome and their down-stream consequences for gene expression and oncogenic cellular behavior. Cellular transformation has been studied mostly in rodent in vivo and in vitro models, but a wealth of knowledge comes from experiments conducted in human cultured cell systems. Some of the underlying species differences in the genetic and epigenetic alterations that drive oncogenic transformation are known but others remain to be defined [284]. Emphasis should be given to transformation of human cells in order to provide the most species-relevant evaluation of causal pathway perturbations.

Cell transformation entails the acquisition of observable phenotypic alterations that are hallmarks of tumourigenic cells (e.g., immortality, differentiation blockade, tissue invasiveness). In vitro, the phenomenon of morphological cell transformation of primary diploid cells involves changes in behavior and growth control of cultured cells, such as alterations to individual cell morphology or disorganized patterns of colony growth. Upon further growth in culture, and often accelerated by further chemical treatments, transformed cells gain an ability to grow in semi-solid agar (anchorage-independent growth), produce autocrine growth factors and can evolve to tumourigenicity when injected into appropriate hosts. To provide sufficient replicative life-span in culture in order to achieve all of these aspects of transformation, the cells may also need to acquire the ability to divide indefinitely (immortalization). The immortal state is often associated with other alterations including aneuploid karyotypes and altered genetic stability. The general assumption has been that cellular and molecular processes involved in cell transformation in vitro at least partially recapitulate processes occurring during carcinogenesis in vivo [285,286]. The following sections provide some of the accumulated molecular evidence in human cells and some similar, convincing experimentation in animal cells that strongly supports the idea that chemical perturbations to epigenetic controls on gene expression may be causal steps in cell transformation, keeping in mind the criteria for experimental establishment of causality set out in Table S1.

### 7.1. The “Epigenetic Progenitor” Hypothesis and Multi-Step Tumourigenesis

Karpinets and Foy hypothesized that a sustained stress environment, created by chemical or physical stressors, causes epigenetic reprogramming that is dependent on activation of stress-related survival signaling in cells [287]. They surmised that stressful conditions play a leading role in tumourigenic transformation from their review of the many independent studies that showed DNA hypermethylation and hypomethylation in precancerous and early cancer stages, together with evidence showing that similar epigenetic alterations were created in rodent and human cells. They extended this idea to suggest that the early epigenetic alterations set up conditions for continued epigenetic and genetic alterations that drive cancer development and progression. Also, building on the many observations of early epigenetic alterations in human cancers, Feinberg et al, [288] highlighted the body of associative evidence pointing to a crucial role for polyclonal epigenetic disruption of stem or progenitor cell populations and, from those observations, derived the “epigenetic progenitor” hypothesis which proposed that epigenetic perturbation of cellular developmental processes is a fundamental driver of cancer initiation and progression.

According to the combination of these carcinogenesis models, a tumour would frequently arise in a three step process:

- Epigenetic alterations of stem or progenitor cells create disturbances to the regulated expression of tumour-progenitor genes that normally function to promote “stemness,” as characterized by pluripotency and replication capacity while repressing differentiation (examples include *Igf2* up-regulation by hypomethylation/loss of imprinting [289]; *Latexin* down-regulation by hypermethylation [290]). The alterations in expression at this early stage probably frequently result from environmental perturbations in epigenetic processes (writers, erasers, editors or readers). Consequently, increases or persistence of the more primitive precursor cells within a tissue, lead to the disruption of the balance between undifferentiated progenitor cells and differentiated cells, as well as disrupting the capacity to differentiate along specific differentiation program paths. It should be noted that the epigenetic progenitor hypothesis includes the idea that, in the relatively undifferentiated progenitor or tissue stem cells, some properties characteristic of advanced tumours may already exist (e.g., self-renewal, invasiveness, or drug resistance).
- Tumour-suppressor gene inactivations and/or oncogene activations occur within the expanded or altered progenitor compartment, removing further constraints on cell replication and survival.
- Gains of constitutive genetic, chromosomal and epigenetic instability lead onward to an increased pace of tumour evolution.

### 7.2. The Syrian Hamster Embryo (SHE) Cell Transformation Assay

One of the first experiments to experimentally link cell transformation-related phenotypic changes to an epigenetic perturbation, resulting from chemical treatment, was conducted in primary Syrian hamster embryo (SHE) cells [291]. The SHE cell transformation assay has a long history of use as a short-term assay (7 days for colony formation) to detect chemical agents that have potential to be animal or human carcinogens [292]. It therefore was of interest to investigators to provide some experimental insight into the molecular underpinnings of the transformation event. At that time, the *H19* non-coding RNA was known to be unexpressed in human embryonic tumours or in undifferentiated cells. Conversely this RNA species was dramatically up-regulated during several cell differentiation programs. On this basis, it was hypothesized that the cell transformation event in culture might represent a phenotypic consequence derived from an underlying block in the cellular differentiation program. In keeping with this idea, the authors showed that primary SHE cell cultures expressed *H19* RNA, while 75% of the benzo[*a*]pyrene- or 3-methycholanthrene-transformed colonies (identified as transformed upon reaching approximately 200 to 500 cells) had fully lost its expression. The loss of expression continued in animal tumours derived from the transformed cells, many generations after the chemical exposures, therefore demonstrating a mitotically stable change had occurred. Loss of *H19* expression was correlated with hypermethylation within the gene as revealed by a loss of methylation-sensitive restriction enzyme cutting sites within the *H19* locus in DNA obtained from both the transformed cells or subsequently from animal- derived tumours. Re-introduction of a functional *H19* RNA into the transformed cells was accomplished by transfection of a plasmid-based expression vector which resulted in a high level of *H19* RNA in those cells. Re-expression of *H19* RNA in this way caused reduced tumour size and increased tumour latency relative to *H19* non-expressing controls when transfected cells were injected subcutaneously into athymic nude mice. It is important to note, in support of the epigenetic reprogramming theory of carcinogenesis, that expression of *H19* and expression of other differentiation-associated genes, is consistently lost when mouse embryonic fibroblasts are experimentally reprogrammed with transcription factors to become pluripotent stem cells (iPSC) or when the fibroblasts are oncogenically transformed by transfection with oncogenes [293]. Furthermore, oncogenic foci and iPSCs were quite similar in their transcriptional profiles, based on a genome-wide transcriptomics analyses. Taken together, the data support the suggestion that the loss of *H19* expression in SHE cell morphological transformants is part of a chemically-induced epigenetic reprogramming of immature tissue stem or progenitor cells. The reprogramming acts as an impediment to developmental gene expression programs that can impact colony morphology in the tissue culture environment. If this viewpoint can be substantiated with further molecular studies on the mechanisms controlling *H19* during transformation, it is likely that additional biomarkers, based upon up-regulated or down-regulated genes affected by shared epigenetic derangements, could be applied to earlier detection of the cells forming the transformed colonies. The most accurate biomarkers would likely be those aberrantly-regulated expressions, like *H19*, shown to be causally connected to the SHE cell transformed colony phenotype.

### 7.3. p16 in Syrian Hamster Dermal Fibroblast and Embryo Cell Immortalization

The multi-step tumourigenesis model starting with a dysregulated epigenetic progenitor cell is in keeping with observations made in cultured primary Syrian hamster dermal fibroblasts transiently exposed to benzo[*a*]pyrene or nickel chloride (a non-mutagenic carcinogen) [294]. The in vitro exposures generated immortal variants that could be selected for continued growth after senescence of the bulk culture of primary cells (at around 50 passages). Over this extended period in culture, the variant cells were expected to accumulate sufficient epigenetic and genetic variations, enabling bypass of the stress-induced senescence barrier that was induced by continuous passages in culture. In keeping with this expectation, reduced or absent expression of *p16/Ink4a/Cdkn2a* tumour suppressor gene mRNA was observed in the recovered immortal clones. Expression of *p16* mRNA was re-activated by treating the cells with the DNA demethylating agent, 5-aza-2′-deoxycytidine, plausibly indicating that repression of *p16* gene expression had been mediated by a hypermethylation event. Hypermethylation was confirmed by sodium bisulfite-converted DNA sequencing of the *p16* gene promoter region. Epigenetic restoration of *p16* expression by 5-aza-2′-deoxycytidine resulted in a rapid return to the senescent phenotype in the treated cells. In the case of nickel chloride, hypermethylation of one allele in immortal cells was often accompanied by loss of the other allele of the *p16* gene. These experiments have been repeated, very recently, in the SHE cell transformation assay. Benzo[*a*]pyrene induced morphological transformants were picked and serially cultured up to the point of culture senescence at 100 population doublings [295]. At various points in the culture passages (immediately to later), immortal populations emerged from about 20% of the transformed clonal cultures. Extensive promoter methylation of the *p16* gene promoter occurred in 45% of the immortal clones, along with lower levels of methylation in another 27% of clones. As was the case with the dermal fibroblasts, monoallelic deletion of the *p16* locus was observed in some cases. Again, treatment of immortal SHE cells with 5-aza-2′-deoxycytidine lead to loss of methylation at the *p16* promoter, along with a decrease in cell division rates and an increase in cells with a senescent phenotype, implicating the loss of expression of *p16* as a driver of immortalization.

Although these experiments were conducted in primary rodent cells, they point to the likely possibility that measurable genetic or epigenetic events in critical human cell cycle regulatory genes, such as loss or suppression of the cyclin kinase inhibitor p16 in the pRB tumour suppressor pathway, could be employed as suitable biomarkers in a causal pathway of events leading to an adverse biological change in growth behavior. Replicative immortality is considered a hallmark capability acquired by human tumour cells during multi-stage development of cancer [296]. In this case however, the epigenetic restoration of *p16* might not be the only event associated with the 5-aza-2′-deoxycytidine induced senescent phenotype, and other epigenetic alterations as well as 5-aza-2′-deoxycytidine induced stress [297,298] cannot be discounted as contributors to the restored senescent phenotype. In the following section we discuss the importance of *p16*, as revealed by direct experimentation in human cells.

### 7.4. p16 in Human Mammary Epithelial Cell Escape from Culture “Stasis”

Human primary mammary epithelial cells provide an in vitro model that is amenable to detection of early epigenetic alterations caused by environmental stressors [299]. Exposure of the mammary epithelial cultures to benzo[*a*]pyrene, or the stress of serum-free medium, causes some cells within to overcome the culture-induced barrier termed “stasis” (also called stress-induced senescence, SIS) and the cells often become visible as a subpopulation of morphological variants (vHMEC) within cell monolayers. Stasis is defined as a proliferative halt mediated by p16/INK4A inhibition of CYCLIN D-mediated phosphorylation of retinoblastoma protein thereby allowing unphosphorylated pRB to sequester E2F transcription factors and down-regulate the proliferation-related target genes (Figure 1). Epithelial cell clones that escaped the stasis barrier were shown to contain many tens to several hundreds of differentially methylated regions (DMRs) as determined by methylated DNA immunoprecipitation (meDIP) followed by microarray analysis. In addition to hypermethylation of many genes involved in cell adhesion, extracellular matrix and cell membrane, the post-stasis variants from these cultures displayed *p16/INK4A* gene inactivation due to promoter hypermethylation. Continued growth of vHMEC causes cells to accumulate a range of chromosomal abnormalities. It has been shown experimentally that in HMEC and several other primary cell types, short hairpin silencing of *p16/INK4A* (giving approximately 50% suppression) causes centrosome dysfunction and related aneuploidy [300]. Genomic instability resulting in gains and losses of genomic material can provide the extended lifespan cells with powerful mechanisms to progress toward gains in proliferative potential and losses of apoptotic responses.

These early epigenetic alterations associated with proliferative transformations in primary human cells thus parallel some of the alterations measured in the Syrian hamster dermal cell transformation system. To show the importance of human p16/INK4A function in maintaining tissue culture growth stasis, stable, targeted knockdown of *p16/INK4A* gene expression by retroviral transduction of a shRNA expression construct permitted bypass of stasis, interestingly with few DMRs detectable in the genome of the recovered knockdown clones [299]. Thus, these results were interpreted as showing that stress- or chemically-induced epigenetic disturbances to the DNA methylome generated a population of phenotypically variant cells, some of which attained stable and sufficiently repressed *p16/INK4A* expression that allowed continued replication beyond the stasis phase, even in the absence of the original transforming treatments (serum starvation, or benzo[*a*]pyrene treatment).

### 7.5. Roles for Epigenetic Changes during Chemical Transformation of Human Cell Lines

A collection of (twenty) related studies examining causal connections between chemically-induced epigenetic perturbations and aspects of more progressed transformed cellular phenotypes in human cells are listed in Table 5. These types of studies differ from the primary SHE cells and primary human mammary epithelial cell models by employing various immortal human cell lines established from lung, breast, prostate, and bladder epithelia. The immortal cells were treated with several different organic and inorganic carcinogens, possessing either genotoxic (benzo[*a*]pyrene, benzo[*a*]pyrene diol epoxide, methylnitrosourea, cigarette smoke condensate, cadmium chloride, nickel chloride) or non-genotoxic (arsenic, 2,3,7,8-tetrachlorodibenzodioxin) properties. The phenotypic endpoint most often measured in these studies centered on selectable malignant transformations producing variant cells that have the capacity to grow without anchorage within a soft agar culture medium. The transformed cells arising from these experiments are thought to correspond to cells that have undergone steps 1 and 2 in the outline multi-step tumourigenesis model. The transformation experiments, in this case, would therefore most likely mimic the clonal outgrowth of preneoplastic cells in a tissue setting (preneoplastic tissue field), wherein epigenetic alterations contribute to a more advanced tumour pathogenesis. Examples of changes in gene expression caused by chemically-induced hypermethylated and hypomethylated DNA at gene promoters, as well as increases in binding of polycomb repressive complex are given. Reversal of the epigenetic effects produced by chemical inhibitors of DNA methylation and chromatin remodeling enzymes or engineered re-establishment of gene expression states affected by the epigenetic perturbations had significant effects on reducing the malignant properties of the transformed cells in those studies.

A developing idea, not exemplified in Table 5, is the possibility that chemical treatments may set in motion concerted epigenetic changes culminating in cell transformation. In support of this notion, experiments have implicated loss of retinoic acid signal transduction pathway activity in an ordered, non-random, re-programming of chromatin and DNA methylation of a functional gene network thought to be involved in the control of cell growth, differentiation, and organogenesis. In this work, impairments in retinoic acid signaling through retinoic acid receptor α (RARα) or the cellular retinoic acid binding protein 2 (CRABP2, transporter of retinoic acid from cytoplasm to the nucleus and RAR) were created by stable gene expression knockdown in immortalized human breast epithelial cells [301,302]. These engineered changes lead to successive epigenetic silencing of the *RARβ2* gene (a direct transcriptional target of RARα) and the genes for cellular retinol binding protein 1 (*CRBP1*) and *CYP26A1* (a P450 retinoic acid-specific hydrolase), that are direct transcriptional targets of RARβ2. Silencing at these loci involved both hypoacetylation of histone 4 and DNA hypermethylation. Highly diminished *RARβ2* transcriptional activity due to establishment of an homozygous repressive epigenotype at both alleles of this gene and throughout the network of its target genes, particularly *CRBP1*, lead to several phenotypic changes involved in breast epithelial cell transformation, such as loss of epithelial polarization, inability to form a hollow lumen in 3D basement membrane cultures, and anchorage-independent growth. The specific mechanism(s) involved in targeting epigenetic modifiers to these gene loci remain unknown. It is interesting to note, with respect the epigenetic effects cascade controlled by retinoic acid, that others have more recently found that 24 h exposure of human oesophageal cancer cells to benzo[*a*]pyrene diol epoxide (BPDE) suppressed expression of *RARβ2* gene expression by an induced methylation of its promoter [303]. The promoter methylation and gene expression was apparently due to BPDE-induced recruitment of DNMT3A to the promoter region and was reversible upon 5-aza-dC treatment. It will be important to determine in future work whether BPDE would be effective in heritably methylating *RARβ2* and consequently its target gene network, in the immortalized breast epithelial cell line discussed above, as a model for a chemically-perturbed toxicity pathway leading to heritable oncogenic phenotypic changes.

## 8. Current Developments in Phenotypic Screening Assays for Chemicals Causing Epigenetic Perturbations in Human Cells

During the last decade, there has been growing effort directed toward the design and implementation of human in vitro phenotypic assays for screening chemical libraries, in intact, viable cells, in order to identify and predict potential safety risks as well as to identify candidate chemicals with potential drug activities [320,321,322,323]. Phenotypic screens have the potential to assess the activity of chemicals on various physiological, structural and genetic/epigenetic endpoints. In vivo and to some extent in vitro, these endpoints are operating in a complex, native intracellular environment where an extended range of dynamic molecular interactions may play vital but undefined roles. Molecular target-based screening, on the other hand, relies on highly purified, known molecular targets and measures the outcomes of their direct interactions with the screened chemicals in biochemical assays. Phenotypic assay designs can report on changes to cellular behaviors (cell cycle, apoptosis, motility, secretion, transformation, differentiation), sub-cellular morphologies (nuclear, mitochondrial, cytoskeletal), signal transduction or stress-response pathways (reporter genes), cell viability or metabolic activity. Such assays may also report on mechanistic aspects of cellular phenotypic change through measurements of specific endogenous biomarkers/reporters or engineered reporter genes. With regard to epigenetic pathways, one interesting study systematically and comprehensively targeted epigenetic enzymes in human prostate cancer cells by using an arrayed siRNA methodology, rather than chemicals, to determine functional consequences related to cell proliferation, survival, androgen receptor expression, histone methylation and acetylation [324]. This approach, in theory, could be applied in a variety of cell lines to gauge the phenotypic consequences of changes in epigenetic systems, prior to chemical screens. The cell type chosen for phenotypic assays (differentiated, stem-like, or disease-affected) can provide physiologically and toxicologically relevant screening platforms in which to conduct phenotypic assays [325]. In pursuit of strengthening the mechanistic connections between epigenetic pathway perturbations, *TSG* silencing, and chemically-induced human cell transformation, it would be most helpful to conduct screening projects in cell systems, like those discussed throughout the earlier sections of this review, that could subsequently be used to confirm the abilities of chemicals or their metabolized derivatives to transform cells into oncogenic variants.

Several published studies have presented results from practicable phenotypic, cell-based, high- or medium-throughput chemical screening assays for epigenetic effects targeting epigenetic pathway components (marks, writers, readers, or editor/erasers), or their effects on target genes, in a variety of human and rodent cells (Table 6 and Table 7). Also listed in these Tables are several assays that have near-term potential to be applied to chemical screenings, but have only been tested with limited numbers of chemicals. Essential criteria for high throughput phenotypic screens have been presented [326]. Among the more important issues are that these assays have:

- Capability to be performed in 96- or 1536-well plate formats
- 10 steps or less
- Fast assay times (24–48 h)
- Minimal assay steps, 4 or less
- Robust signals, greater than 3-fold

In Table 6 and Table 7, two broad categories of assays are recognized. The first category includes assays in which readout is an average result from the whole cell population. Suitable measures may be obtained if a significant fraction of the population were affected by a chemical treatment (Table 6). The second categorizes assays with readouts that are made on a cell-by-cell basis, providing increased resolution to changes within the cell population and sensitivity to changes in small portions within the cell population (Table 7). Individual cell measures in the cell-by-cell studies were made by various high content imaging systems with automated image acquisition.

One possible 3-step approach to epigenetic toxicity testing could begin with high-throughput measures of global effects on epigenetic marks (such as global modified histone or DNA methylation levels), confirmed by follow-up measures using medium-throughput assays of specific epigenetic pathway components (writers; erasers/editors, readers) or effects at epigenetic reporter genes or at individual endogenous *TSG* loci. Finally, lower-throughput phenotypic measures related to oncogenic transformation (Tier 2 assays [19]) could be employed to confirm that the changes in key pathway steps do, indeed, have consequences for in vitro cellular behaviors relevant to oncogenesis. It is notable that some high-to-medium throughput chemical screenings have already been carried out for effects on several of the global epigenetic marks and the epigenetic readers/writers/erasers that were covered in this review, including BMI1, a polycomb repressive complex 2 (PRC2) subunit; HDAC (classes I and II) activity; JMJD3 activity, an H3K27me3 demethylase; H3K27ac and H3K27me3, the PRC2 epigenetic mark. However, the cell lines employed in these studies were generally tumour-derived lines.

It will be important, from a toxicological perspective, to begin to develop protocols that would allow such assays to be performed with untransformed cell lines and perhaps primary human cell cultures in which causal connections to increases in oncogenic phenotypes can be made in the higher tier assays. It has also been suggested that existing, validated toxicological tests could be adapted to include epigenomic studies [338]. In this regard, the in vitro mammalian chromosomal aberration test (O.E.C.C. Test Guideline 473; TG473), specifies that primary human cells may be employed, making this assay a likely candidate for exploration of the idea of augmenting existing tests.

## 9. Conclusions

Testing for chemical hazards in vitro, as envisioned by the Tox21 strategy, requires cellular- and molecular-level insights into the key, causal steps along pathways of toxicity in human cells. A principal goal for this review was, therefore, to survey published studies containing direct experimental evidence demonstrating that epigenetic disruptions are a carcinogenic mode of action, producing altered gene expression patterns that influence human oncogenic transformation in vitro with relevance to in vivo tumours. A current overview of epigenetic pathway components, marks, writers, editors/erasers and readers, of DNA methylation and HPTM was provided, with an emphasis on those players capable of creating stable epigenetic changes in gene expression known to participate in carcinogenesis, in particular, among known tumour suppressor genes. Examples of engineered biological systems or specific molecular tools that were used to generate perturbations of twenty five epigenetic toxicity pathway components showed that conditions or activities outside of the ranges measured in model human cell cultures were “drivers” of cellular transformation events in those cultures (underlined in Table 1 and Table 3 and included in Figure 4). The accumulating evidence for the oncogenic consequences of these perturbed functions raises the expectation that, when similar in direction and magnitude, those caused by exposure to chemicals would also function as toxicity pathways leading to transformed cell variants. Several studies were summarized, in which xenobiotics were shown to disrupt the regulation of epigenetic enzymes and proteins that form components of putative epigenetic pathways of toxicity and that the disruptions lead to the suppression of known tumour suppressor genes (*TSG*s) playing roles in transformed phenotypes of primary or immortalized human cells.

**Figure 7:**
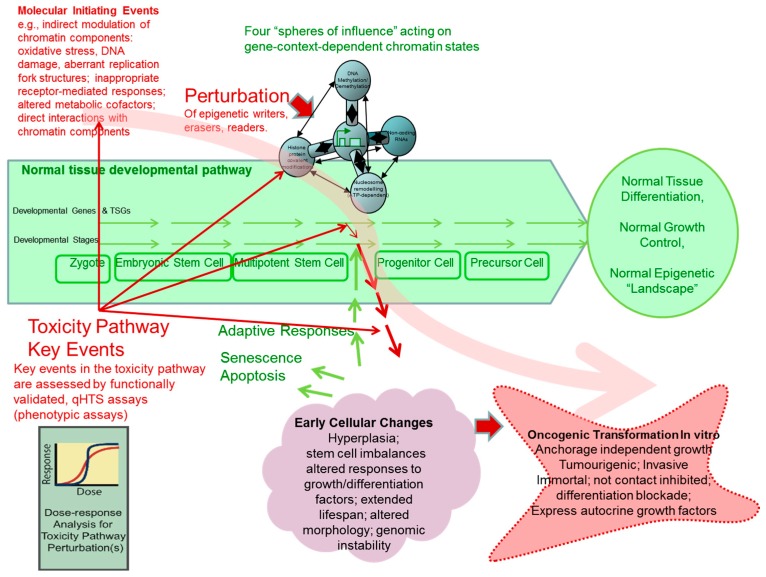
An adaptation of the Pathway of Toxicity (PoT) and related Adverse Outcome Pathway (AOP) concepts for hazard identification and dose-response assessment, according to the Tox21 vision for toxicity testing (TT21C): a high-level, hypothetical scheme depicting epigenetic toxicity pathways to oncogenic cell transformation within a cell development context. The normal developmental pathway is depicted, in green, showing cellular developmental stages with growth and differentiation controlled by sets of developmental and *TSG*s and leading to fully differentiated tissue cells. The toxicity pathway (crossing the normal pathway, in red) is highlighted by red arrows indicating aberrant, but stable, gene expression states that function in transformed phenotypes. In this context, mode of action for an epigenetic carcinogen is considered as a toxicity pathway of key events, leading through the four “spheres of influence” affecting chromatin alterations (see Figure 4), to epigenetically silenced/repressed cell cycle checkpoint genes; *TSG*s and differentiation genes controlling oncogenic transformation. Multiple key events that are functionally validated steps within the toxicity pathway are chosen as targets of quantitative high throughput screening assays. Dose-response results are subsequently employed in dose-response analyses and modelling for quantitative risk assessment purposes within the NexGen framework for risk science [19].

Human lung and bronchial, breast, prostate and bladder epithelial cells (primary or immortalized), representing the four tissues characterized by the highest cancer incidences, were the most frequently applied models in the functional characterizations of molecular- or xenobiotic-induced perturbations of epigenetic pathways leading to oncogenic transformation in vitro. Human cellular models of oncogenic transformation provide highly relevant contexts for further development of in vitro chemical testing of epigenetic toxicity pathways and, under Tox21 assessment strategies, should provide useful information for human health risk assessment and decision-making processes by chemical regulatory agencies. Figure 7 presents a high-level, hypothetical schematic depicting the application of the epigenetic endpoints, discussed in this review, to in vitro human carcinogenic hazard identification and dose-response assessment.

Causal epigenetic events have been identified among the human cell transformation models presented in this review, it should be a near-term priority to develop and employ aspects of these particular events as functional biomarker targets for quantitative high through-put screens of chemical libraries. The review has emphasized the epigenetic repression of *TSG* expression as a mode of carcinogenic action with potential for application within the TT21C testing and assessment paradigm and it will be particularly important to develop existing knowledge around *TSG* epigenetic silencing into informative screening assays. In this regard, there is need for information in several areas: (1) knowledge of the contributions to cell transformation resulting from silencing events among the wide range of known *TSG*s in the cell models chosen for study; (2) calibrations of changes in the toxicity pathways that belong to differentiation or physiological processes considered as normal in those cell models; (3) determinations of the magnitudes of changes forming “tipping points” that begin to contribute to disease and carcinogenic processes; (4) measures of persistence of the epigenetic changes; and (5) reproducible concentration-response and exposure-duration curves that would allow extrapolation of the adverse concentrations to tissue concentrations expected to occur in exposed humans. One specific obstacle that may need to be overcome in this endeavor is that cell transformation as a phenotypic endpoint and its underlying epigenetic changes that act to effectively silence *TSG*s may be longer term events resulting from chronic chemical exposures. The relatively short time-frames currently employed in high throughput Tier 1 TT21C testing strategies will necessitate validations of reporter-gene constructs based upon *TSG* targets, or any other epigenetic assays based on cell-by-cell measures, to determine if they will be sufficiently sensitive in short term assays. Application of multiple, confirmatory approaches to identifying further targets of epigenetic toxicity and to querying how perturbations of these targets lead to the phenotypic endpoint of cell transformation will benefit the uptake of this mode of action into cancer risk assessments. Some general considerations on applicable technologies and biological issues in the traditional weight-of-evidence-based assessment of epigenetic carcinogens have previously been offered [6,339]. Going forward, case studies on chemicals for which there is substantial prior information in regards to their epigenetic activity (such as the human carcinogen arsenic) will be useful in developing quantitative high throughput assay readouts for key toxicity pathway events, and will facilitate proposals for interpretive tools intended to present those readouts as effective information in human safety assessments according to the TT21C vision.

## Figures and Tables

**Table 1:** Classification of functions among the well-studied, cancer-associated histone modification systems *.

Histone Modification/Marks (Associations with Activated or Repressed Transcription)	“Writer” Enzymes (Transferases)	“Eraser” Enzymes	“Reader” Proteins and Protein Domains that Transduce Epigenetic Information
1. Lysine Acetylation (active transcriptional start sites, enhancers)	Lysine acetyl transferases (KAT)	Histone deacetylases (HDACs)	Tandem bromodomains; (e.g., BRG1 ATPase of SWI/SNF remodelling complex)
H3K9ac	KAT2A	SIRTUIN1,6 (class III HDAC; NAD+ dependent)	Tandem PHD fingers
H3K27ac	p300, CBP		
H4K16ac	MOZ/MYST3/KAT6A	SIRTUIN 1,2	
2. Lysine Methylation	lysine methyltransferases (KMT)	Lysine demethylases (KDM)	Bromodomains
H3K4me3 (active transcriptional start sites/TSS)	SETD1A,B; MLL1/KMT2A; MLL2; MLL3; MLL4	KDM4/JMJD2; KDM5/JARID1A,B	PHD fingers (inhibitor of growth/ING); Tudor domains (53BP1);
H3K4me1 (activated enhancers)	SETD7	KDM1/LSD1 (H3K4me2, H3K4me1)	ZF-CW proteins; WD40
H3K36me3 (activated gene bodies)	SET2 (H3K36me3)	JHDM1B/KDM2B (H3K36me3)	PWWP (e.g., DNMT3 A/B, bind H3K36me3)
H3K9me3 (repressed; in constitutive heterochromatin, e.g., pericentomeric, inactive X)	EHMT1/GLP, EHMT2/G9A (H3K9me1,2); SUV39H1, SUV39H2, SETDB1 and SETDB2 (H3K9me2, me3)	KDM3/JMJD1 (H3K9me2, H3K9me1); KDM4A/JUMD2A (H3K9me3)	UHRF1, HP1 (an heterochomatin adaptor protein); MPP8
H3K27me3 (Polycomb- repressed TSS)	EZH1,2 (H3K27me1–me3)	KDM6/UTX, JMJD3 (H3K27me3)	Chromodomains (PRC1/CBX7,CBX8, HP1 bind H3K27me3);
3. Serine/Threonine and Tyrosine Phosphorylation; H3S10, H3S28	Protein kinases (ATM/ATR, PKC, AURORA B, JAK2, etc.)	Protein tyrosine and serine/threonine phosphatases DUSP1	Chromoshadow domains (phosphotyrosine); 14-3-3, BRCA1 C Terminus (BRCT) domain (phosphoserine or phosphothreonine)
4. Lysine Ubiquitination H2AK119ub1 (repressed gene transcription)	Ubiquitin E2 conjugases; E3 ligases (e.g., polycomb repressive complex 1 RING1A/B protein; UHRF1)	Ubiquitin-specific proteases MYSM1	JARID2 from the PRC2 complex [48].
5. Arginine Methylation	Protein Arginine methyltransferases (PRMTs)	Histone demethylases	Tudor domains (for asymmetrically dimethylated arginine); PHD domain (for symmetrically dimethylated arginine)

* see Khare et al. [49] for a more complete catalogue of histone modifications, writers and erasers: the HIstome Infobase includes information for 5 types of histones, 8 types of post-translational modifications and 13 classes of modifying enzymes. (total: approximately 50 individual histone protein variants and 150 histone modifying enzymes, and summaries of their associations with cancer and other diseases). See Chi et al. [50], for a review of known changes in histone writers, erasers and readers found in human cancer. Underlined proteins possess evidence for causal connections to perturbed *TSG* expression or oncogenic transformation of human cells, which is provided in relevant sections of this review.

**Table 2:** Experimental studies employing molecular tools to modify polycomb group (PcG) components and thereby affect human cell growth regulation.

TSG Target	“Reader/Writer/Erasers” Experimentally Perturbed	Experimental Perturbation	PRC or Histone Mark Verification	Cell Model	Adverse Phenotype Modified by Perturbation: ← Reversal of Oncogenic Phenotype; → Enhancement of Oncogenic Phenotype	Reference
*p16/INK4A*	SUZ12, CBX8, BMI1; mRNA down-regulation; loss of binding to *p16* locus	Knockdown (stable shRNA expression from retroviral vector)	Knockdowns of CBX8 or BMI1 reduced the recruitment of both proteins and the *p16*/*INK4A* locus	TIG3-T telomerase-immortalized human fibroblasts	← *p16* up-regulation with decrease growth of TIG3-T cells and reduction of colony formation in U2OS cells	[91]
*p16/INK4A*	JMJD3 (H3K27me3 demethylase) down-regulation or up-regulation	Knockdown (stable shRNA expression from retroviral vector); up-regulation by ectopic expression	Exogenous JMJD3 was recruited to the *p16*/*INK4A* locus, caused reduction in H3K27me3	1° human fibroblasts	→ *p16* down-regulation and partial bypass of RAS oncogene-induced senescence response ← Induced senescence	[92]
*p16/INK4A* and *ARF*	CBX7 up-regulation/down-regulation	Over expression and knockdown (stable shRNA expression from retroviral vector)	No measures of changes to CBX7 abundance at *p16* or *ARF* loci	Variety of human 1° cells	→ *CBX7* overexpression causes *p16* down-regulation and extended replicative capacity *CBX7* repression caused severely impaired growth	[93]
*p16/INK4A*	BMI1 up-regulation	Overexpression by expression vector transfection	No measures of histone ubiquitination on *p16*/*INK4A* chromatin	1° human mammary epithelial cells	→ *BMI1* overexpression suppressed *p16*/*INK4A* expression and weakly induced hTERT activity; p16/INK4A was shown to be required replicative lifetime extension	[94]

**Table 3:** Brief annotated list of “writers”, “editors”, “readers” of the DNA methylation system.

Writers *	Editors/Erasers **	Readers		
		Share methyl-CpG- binding domain (MBD):	Bind hemimethylated DNA through their SET and RING finger associated domain	Share zinc finger DNA binding domain
DNMT1 (Maintenance)	TEt1 (acute myeloid leukemia)	MeCP2 (Rett syndrome; prostate cancer)	UHRF1 (Binds hemimethylated DNA, H3K9me3, and H3R2. E3 ubiquitin ligase. Essential to maintenance DNA methylation)	KAISO (interfere with WNT signaling pathway)
DNMT2 (no catalytic site)	TEt2	MBD1	UHRF2 (prefers fully hydroxymethylated over hemihydroxymethylated DNA)	ZBTB4 (KAISO paralogs)
DNMT3A (de novo)	TEt3	MBD2		ZBTB38 (paralogs)
DNMT3B (de novo)		MBD3		ZFP57 (imprints)
DNMT3L (cooperates with 3A, 3B)		MBD4 (glycosylase)		
		MBD5		
		MBD6		
		SETDB1 (H3K9MT)		
		SETDB2 (H3K9MT)		
		BAZ2A (NoRC complex; rDNA, centromeres, telomeres)		
		BAZ2B		

*: Depend on availability of S-adenosyl-L-methionine as a universal methyl donor produced through functional one-carbon metabolism pathway; **: Depend on normal mitochondrial function, Kreb cycle, isocitrate dehydrogenases (IDH1, 2, and 3), Fe^2+^, and ascorbate. Underlined genes when manipulated were shown to induce human cell transformation (references in text).

**Table 4:** Study examples demonstrating that modulating the expression of DNA methylation “readers”, “writers”, or “editors/erasers” leads to adverse oncogenic events in human cells.

Human Cells	Epigenetic Target	Transforming Event	Outcomes and Evidence of Cell Transformation	Reference
Colorectal cancer cell lines (p53 wild-type HCT116 and LS174T, p53 mutant SW480)	DNMTs	Folic acid supplementation.	Inverse dose-response effects on global genomic methylation but positive dose-response effects on colonosphere formation in vitro in HCT116 and LS174T cells, but not in the SW480 cell line.	[144]
Hepatocarcinoma tissues and SMMC-7721cell line	MeCP2	Down- and up-regulation by siRNA and plasmid, respectively.	*MeCP2* over-expressed in HCC tissues compared to adjacent noncancerous tissues. Up- and down-regulation increase or decrease proliferation, respectively, through modulation of the Sonic hedgehog signaling pathway.	[145,146]
Normal prostate epithelial cells and cancer prostate cell lines (LNCaP, PC3, DU-145)	MeCP2	Down- and up-regulation by shRNA and plasmid, respectively.	Based on growth curves and colony formation assays, down-regulation reduces growth of the normal cells and prostate cell lines, while up-regulation tested in LNCaP cells induce androgen-independent growth and soft-agar colony formation associated with c-MYC protein stabilization.	[143]
Pancreatic (PANC-1, BxPC-3) and prostate cancer (PC3) cell lines	MBD1	Down-regulation by siRNA.	Down-regulation inhibits cell growth and invasion, and induces apoptosis in pancreatic but not in the prostate cell line. MBD1 would be oncogenic in pancreatic, but tumour suppressive in the prostate cell line.	[147]
HBEC3 non-malignant bronchial epithelial cells, and H358 lung cancer cell line	DNMT1	*DNMT1* overexpression and *DNMT1* mutant (deletion of the replication foci targeting sequence).	Overexpression of normal and of mutant *DNMT1* increased anchorage-independent soft-agar colony formation. Pericentromeric DNA repeated sequence (*SAT2*) hypomethylation, but tumour suppressor gene promoter hypermethylation.	[148]
Non-tumorigenic astrocytes (Astro#40), mammary epithelial cells (MCF10A), lung fibroblasts (Wi-38), and mesothelial cells (Met5A)	DNMT1	Disruption of the DNMT1/PNCA/UHRF1 complex by interfering *DNMT1* plasmid.	Induction of cancer phenotype including increased cell proliferation, resistance to irradiation-induced ceall death, injections of cells caused tumours in mice. Global and promoter hypomethylation preceeding chromosomal aberrations.	[148,149]
Human bronchial epithelial cells (HBEC2 immortalized with hTERT and CDK4), and 22 non-small-cell lung cancer cell lines	DNMT3B	Overexpressed (5-20 fold over control) to human tumor levels.	Accelerate carcinogen-induced oncogenic transformation (colony formation in soft agar and acquired mesenchymal-like morphology). Three-fold increase in the number of genes epigenetically silenced.	[150]
Mesenchymal stem cell lineages	DNMT1	(i) Engineered methylated ssDNA targetted to promoter attracting DNMT1. (ii) 5-aza-dC induced promoter hypomethylation.	Gene down-regulation (*HIC1*, *RASSF1A*). Enhanced growth in soft agar. Increased cell migratory transwell invasion. Drug resistence. Soft tissue sarcoma in nude mice.	[151]
Gastric (AGS, SGC7901, MKN45), colorectal (HT29, HT116, RKO, DLD1, SW620)	UHRF1	cDNA up-regulation, or short-hairpin RNA down-regulation.	In both gastric and colorectal cancer cell lines, down-regulation of *UFRF1* reduces cell proliferation, migration and invasion properties, and tumour growth in athymic mice.	[48,152,153]
Bladder invasive (253J, T24, and KU7) and non-invasive (RT4, RT112, DSH1) cancer cell lines	UHRF1	Engineered up-regulation, or siRNA down-regulation.	Increase and decrease transwell invasion. *UHRF1* up-regulation hypermethylates and silences the metastasis suppressor *KiSS1*.	[154]
Hepatocarcinoma cell lines (HepG2, HCCLM3)	UHRF1	Down-regulation by siRNA	Down-regulation decrease cell proliferation, transwell migration and invasion, and tumour growth in nude mice. Up-regulated in patients with hepatocarcinomas. Patients with elevated expression had shorter relapse-free survival time.	[155]
Breast cancer cell line (MDA-MB-231-1833)	TET1 and its target HOXA gene cluster	Up-regulation of *TET1* leading to its auto-up-regulation.	Suppress cell invasion in vitro, and xenograft tumour growth and invasiveness.	[156]
Non-malignant bronchial epithelial cells (HBEC3) and metastatic non-small cell lung cancer cell ine (H1299)	TET1	Transfection of *RAS* oncogene	Suppressed *TET1*, *TSG* and *H19* expression, and induce soft-agar colony formation as oncogenic cell transformation.	[157]
MCF7 and MCF10 breast cancer and epithelial cell lines, respectively	TET1, 2, and 3.	Engineered expression of *miR-22*	Down-regulation of *TETs*. Epithelial-mesenchymal transition driven by *miR-200* in MCF10, non-metastatic to metastatic properties in MCF7 cells.	[158]
Primary epithelial colon cells and colon cancer cell lines (Caco-2, SW48)	TET1	Down- and up-regulation by shRNA and Doxycycline-inducible *TET1*-promoter.	Down- or up-regulation promote or decrease cell proliferation, respectively. Up-regulation decreases colonosphere formation in vitro and tumor size from injected cells in nude mice. Hypermethylation and down-regulation of WNT pathway inhibitors (*DKK3*, *DKK4*).	[159]
Primary cells and hepatocellular carcinoma cell lines (Hep3B, MHCC97L)	SETDB1	Down-regulation by shRNA	Reduce cell proliferation in vitro, suppressed orthotopic tumorigenicity and metastasis in vivo. Associations with clinical prognosis.	[160,161]
Primary cells and gastric cancer cell lines (MKN74, MKN45, AGS, NUGC3)	SETDB2	Down-regulation by siRNA, up-regulation by vector transfection.	Decreased and increased cell proliferation, migration, and invasion, respectively. Associations with clinical prognosis.	[162,163]
Prostate normal epithelial (RWPE1) and metastatic cancer cell lines (PC3, LNCaP, DU145)	BAZ2A	Down-regulation by siRNA	Reduce metastatic cancer cell proliferation, invasion and migration based on in vitro assays. Associations with clinical prognosis.	[164]

**Table 5:** Selected in vitro experimental studies describing causal, toxin-induced epigenetic pathways leading to cancer-like phenotypes. Experiments in human and rodent in vitro cell transformation systems establishing mechanistic links: (including experiments that “show it; block it; induce it”). Rows are arranged by cell type.

Cell Type	Carcinogen(s)	Epigenetic Perturbations Associated with Treatment	Experiments Blocking (Reversal) or Inducing a Molecular Perturbation Associated with Chemical Treatment	Phenotypic Effects of Molecular Perturbations	References
Syrian hamster embryonic (primary)	Benzo[*a*]pyrene, 3-MCA (5–10 µg/mL)	*H19* long non-coding RNA expression suppressed in transformed colonies; loss of HpaII DNA restriction enzyme cutting; hypermethylation	Transfection/plasmid -based re-expression (reversal)	*H19* re-expression caused faster growth in vitro; slower tumour growth in vivo	[291]
Syrian hamster dermal (primary)	Benzo[*a*]pyrene (1–2 µg/mL), NiCl_2_ (250 µM)	*p16* promoter hypermethylation; silencing of *p16* expression	Reversal of *p16* repression with 5-azaC treatment (demethylating)	Associated with immortalization (post-stasis/SIPS); induced re-expression associated with return to senescence	[294]
Human breast epithelial cells (primary)	Benzo[*a*]pyrene (1 µg/mL)	10 s–100 s–1000 s stepwise DNA hyper/hypomethylation events by CHIP-microarray analysis; *p16* expression suppressed	Inducing low *p16* expression (shRNA knockdown) permitted more frequent bypass of stasis	Associated with post-stasis (SIPS); post- replicative senescence bypass caused by reduced *p16* expression.	[299]
RWPE-1, UROtsa immortal human prostate epithelial and urothelial cells	Arsenite (1 µM)	100 s–1000 s hyper/hypomethylation events; link between histone3-lysine 9-trimethylation domains of stem cells and DNA hypermethylation	–	Associated events with oncogenic transformation	[304]
RWPE-1 human prostate epithelial cells (immortal)	Arsenic (100 ng/mL) and Estrogen (100 pg/mL), in combination	Hypermethylation-mediated silencing of *MLH1*	–	Associated with oncogenic transformation	[305]
RWPE-1 human prostate epithelial cells (immortal)	Cadmium chloride (10 µM)	Global hypermethylation; *p16*, *RASSF1A* promoters hypermethylation; reduced expression of *p16*, *RASSF1A* tumour suppressor mRNAs; increased DNMT activity	Reversal of *p16* and *RASSF1A* repression with 5-azaC treatment (demethylating); partial reversal of repression with procainamide (specific DNMT1 inhibitor)	Global and gene-specific hypermethylation associated with tumourigenicity in nude mice	[306]
16HBE human bronchial epithelial cells (immortal)	NiS (1–2 µg/cm^2^)	DNMT1 up-regulation; *MGMT* gene silencing; promoter hypermethylation; reduced histone 4 acetylation; reduced histone 3 lysine 9 acetylation/methylation ratio	Block *DNMT1* expression with shRNA in transformed cells	Blocked DNMT1 reduced growth of the transformed cells; caused re-expression of *MGMT* by reversion of epigenetic changes	[307]
Human bronchial epithelial cells (immortal)	MNU (500 µM) and benzo[*a*]pyrene diol epoxide (BPDE, 50 nM)	Growth in soft agar; DNMT up-regulated; several genes with hypermethylated promoters	Block *DNMT1* expression with stable shRNA;reversal of repressed miR-200 family epithelial mediators with 5-azaC treatment (demethylating)	Blocked *DNMT1* expression prevented chemical transformation; reversed transformed phenotype and gene silencing	[308,309]
Human bronchial epithelial cells (immortal)	MNU (1 mM) and benzo[*a*]pyrene diol epoxide (BPDE, 100 nM)	*DNMT3B* overexpressing clones were transformed more frequently by chemicals; many polycomb-targeted genes (*MAL*, *OLIGO2*) were hypermethylated and stably marked with H3K27Me3 and H3K9Me2 repressive marks in transformed cells (PRC2 thought to be recruited by the hypermethylation)	Re-expression of silenced genes was achieved by treatment with 5-Aza-dC (DNA methyltransferase inhibitor) and with Trichostatin A (histone de-acetyltransferase inhibitor)	Cells transformed to anchorage-independent growth; plasmid-based re-expression of *MAL* and *OLIGO2* suppressed anchorage-independent growth in vitro and tumour growth in nude mice	[150]
Human lung tumour cell lines (A549, Calu-6)	Tobacco smoke condensate (0.0005–0.003 puffs/mL)	Tumours in nude mice enhanced; recruitment of Polycomb group proteins (BMI1, SUZ12, Ezh2, SirT1) to suppressed *DKK1* gene (an inhibitor of WNT pathway)	Induced WNT achieved by blocking (using stable shRNA knockdown) of *DKK1* (a WNT pathway inhibitor); knockdown of Polycomb proteins relieved *DKK1* suppression	Experimental WNT up-regulation enhanced tumourigenicity in nude mice: *DKK1* remained silenced in tumours	[310]
Human lung tumour cell lines (A549; H292)	Tobacco smoke extract (0.6–2.5%)	Demethylation of synuclein-gamma (*SNCG*) CpG island; increased expression of *SNCG* in A549 *SNCG*-nonexpressing cells	siRNA knockdown of *SNCG* mRNA; exogenous expression of DNMT3B decreased *SNCG* expression in H292 *SNCG*-expressing cells with an unmethylated CpG island	*SNCG* knockdown during tobacco smoke extract exposure suppressed the invasive phenotype induced by the extract	[311]
Primary airway epithelial cells; immortal bronchial epithelial cells; lung cancer cell lines	Cigarette smoke condensate (25 µg/mL)	Repression of microRNA *miR-487b* expression by CSC ; up-regulation mRNA for *miR-487b* targets that are promoters of malignant growth; increased *miR-487* genomic methylation and nucleosome occupancy	*miR-487b* repression reversed with 5-aza-dC methylation inhibitor	Engineered expression of *miR-487b* restored proliferation and invasion of lung cancer cells in vitro and in vivo	[312]
Immortalized human bronchial epithelial line	MNU (500 µM) and benzo[*a*]pyrene (50 nM)	Increased histone deacetylase expression; increased *DNMT1* expression; hypermethylation of oncogenesis-related gene promoters	*HDAC* overexpression causes *DNMT1* expression throughout cell cycle; *HDAC* knockdown or inhibitor (valproic acid) decreases DNMT1 levels; the inhibitor decreased methylation of silenced genes, reactivating expression	Carcinogen-induced anchorage-independent colony formation; HDAC inhibitor decreased this	[313]
Adenovirus 12/SV40 hybrid virus-immortalized human bronchial epithelial cell line BEAS-2B	NiCl_2_ (100 µM), or 1% O_2_ (hypoxia)	H3K9me2 repressive mark increased at Spry2 gene promoter; JMJD1A histone demethylase activity decreased; *SPRY2* gene expression (mRNA and protein) decreased	*SPRY2* over-expression from stable transformation of an expression vector	Increased *SPRY2* expression decreased NiCl_2_-induced anchorage independent growth	[57]
Adenovirus 12/ SV40 hybrid virus-immortalized human bronchial epithelial cell line BEAS-2B	CdCl_2_ (10-100 nM)	Transformants displayed increased anchorage-independent growth; increased migration; *MGMT* down-regulated by epigenetic mechanisms, with diminished capacity to repair alkylated DNA damage	5-aza-dC inhibitor of DNA methylation; sodium butyrate inhibitor of histone deacetylation	*MGMT* expression was increased in the transformed cells	[314]
Neonatal human epithelial keratinocytes	2,3,7,8-TCDD (1 nM)	TCDD repressed transcription of *p16/INK4A*, *p14/ARF*, *p53*, *Rb* genes; *p16* promoter DNA was methylated in response to TCDD	5-aza-dC treatment reversed *p16/INK4A* and *p53* repression by TCDD	-Association with extended lifespan in culture	[315]
Immortal adult skin keratinocytes (HaCaT)	Arsenite (1 µM)	Arsenite repressed expression of Let-7c miRNA	5-aza-dC inhibitor of DNA methyltransferases prevented arsenite-induced repression of *Let-7c* miRNA; overexpression of *Let-7c* by transfection blocked activation of the RAS/NF-kB signalling pathway	-Overexpression of *Let-7c* miRNA reduced spheroid formation in non-adherent dishes and colony formation in soft agar by arsenite-transfomed cell; -overexpression of *Let-7c* RNA abolished tumour growth in nude mice	[316]
Human urothelial cells (immortal)	Sodium arsenite 0.5 µM	Overexpression due to hypomethylation of lipocalin-2 gene promoter; anchorage-independent growth in vitro	Blocked expression: stable lipocalin-silenced transformed cells	Lipocalin-silenced transformed cells showed significantly less anchorage-independent growth	[317]
Immortal human urothelial (UROtsa)	As(III); monomethyl arsenous acid (MMA, 50 nM); NaAsO_2_ (1 µM)	*WNT5A* gene expression greatly increased in malignantly transformed variants of UROtsa cells even in absence of the agents; oncogenic transformation was associated with decreased repressive histone marks (H3K27me3; H3K9me2) and increased activating histone marks (H3K9 and14Ac; H3K4me2) in *WNT5A* promoter region	Histone deacetylase inhibitors trichostatin A and sodium butyrate activated *WNT5A* gene expression in the hypoacetylated parental UROtsa cells; siRNA knockdown of *WNT5A* expression	Knockdown of *WNT5A* expression inhibited anchorage independent growth	[318]
Human B lymphoblast HMy2.CIR line	CdCl_2_ (5–100 nM)	Increased cell proliferation; increased *DNMT1*, *DNMT3B* mRNA; increased global DNA methylation; decreased *p16* mRNA, protein; increased *p16* CpG island methylation	5-aza-dC inhibition of DNA methylatransferase reversed the repression of *p16* expression	Cd-stimulated cell proliferation was totally eliminated by 5-aza-dC treatment	[319]

**Table 6:** In vitro cell screening assays with cell-average readouts for effects on epigenetic pathway components.

Assay Name/Epigenetic Target	Brief Methodology	Application/Throughput	Confirming Assays	Comments/Reference
qRT-PCR *BMI1* (PRC1 component, see Section 2.1.1)—a gene negatively regulated by the BAF trithorax SWI/SNF chromatin remodelling complex	Standard RT/PCR method; 18 h cell exposure; lysis; 384 well plates; up-regulated *BMI1* mRNA indicated chemical inhibition of the BAF complex.	Library of ~30,000 small molecules (novel and pharmacologically active); one concentration: 10 μM	▪ *BMI1* luciferase knock-in reporter vector; ▪ qRT-PCR ▪ esBAF-regulated gene battery with other polycomb complex genes	20 compounds identified that mimic a KO of the Brg1 subunit of the BAF complex [141]
RASL—Seq (modified) RNA species encoding epigenetic writers, erasers/editors/readers	▪ Cell lysates, 384-well plates ▪ Ligation of modified oligonucleotide probes on mRNA targets ▪ Barcoding PCR amplification ▪ Pooled samples, Next Gen Seq	▪ Probe sets for 77 RNAs in a small molecule screening (4 doses ea.) for effects on B-cell phenotypes; ▪ Can analyse up to 36,000 samples pooled per sequencing reaction	–	Up to 100 RNAs essential to epigenetic pathways could simultaneously be monitored across 1000+ treatments at multiple doses. With fewer RNA species targeted, more detailed dose-response curves could be generated. A focus would be warranted on the 23 epigenetic pathway components covered in this review with evidence for causal participation in repressing *TSG* expression and increasing cell transformation [327]. A related targeted high-throughput transcriptomics analysis, for 1767 genes, has recently been applied to induced pluripotent stem cell-derived cardiomyocytes and hepatocytes exposed to DMSO-soluble extracts of 21 petroleum substances [328]
MethyLight high throughput RT-PCR assay methylated or unmethylated genomic sequences	▪ Isolate genomic DNA and bisulfite convert ▪ Fluorescence-based PCR with primers and a probe that overlap potential CpG methylation sites.	▪ Mlh1 promoter (target); ▪ Methylation assessed in a panel of 25 human tumour/normal match-paired tissue samples	Bisulfite sequencing analysis	▪ Sensitive to 1 methylated allele among 10,000 unmethylated equivalents. ▪ Authors suggest usage for 1000 s of samples, but MethyLight results have not yet been reported in a chemical library screening. Methylated promoters controlling *TSG* expression that influence cell transformation would be informative targets in treated cell populations [329]
Immunofluorescence detection Specific cell proteins	▪ Proteins in fixed, cultured cells are detected directly in microplates ▪ Primary antibody, secondary antibody, and DNA/cell stains; In Cell Western™	Detection of effects on stem cell differentiated protein (myoglobin), across 4 concentrations, for 30/309 environmental chemicals	–	No reported use to detect chemical effects on essential epigenetic pathway components [330]
Luminogenic HDAC (class I and II) assay (HDAC-Glo I/II)	▪ Cell-based qHTS approach ▪ Cell lines (5) cultured in 1536-well plates ▪ Cells incubated with a cell-permeable HDAC I/II substrate which is converted to a luciferase substrate by deacetylation ▪ Optimized assay conditions	▪ NCATS Pharmaceutical Collection screened for inhibitor activity (2527 compounds, 8 concentrations each) ▪ 43 active compounds identified from diverse structural classes	Selected compounds retested at 11 concentrations in the cell-based assay and in fluorogenic HDAC isoform-specific biochemical assays	Increased HDAC I/II activities were detected in cancer-derived cell lines, as compared to an embryonic kidney cell line, indicating that there is potential to detect activation of HDAC activity by chemicals, as well as inhibition of activity [331]

**Table 7:** In vitro cell screening assays with cell-by-cell readouts for effects on epigenetic pathway components.

Single-Cell Assay Name/Epigenetic Target	Brief Methodology	Application/Throughput	Confirming Assays	Comments/Reference
Locus Derepression Assays Epigenetically silenced reporter gene(s) promoters	▪ Based on a GFP reporter gene under the control of the cytomegalovirus (CMV) promoter stably integrated and epigenetically silenced in the genome of C127 mouse mammary adenocarcinoma cells ▪ 1536-well format; ▪ 30 h incubation ▪ Expression detected with a laser scanning microplate cytometer	Screening of a chemical library of over 280,000 compounds, each in a 6-point concentration series; identified 550 compounds with good quality response curves	▪ GFP mRNA expression (q/RT-PCR) was increased by a tested subset of positive compounds ▪ Selected compounds activated other silenced genes, including *TSG* p21/Cdkn1a, in human cancer cells. ▪ Increased activating and repressive histone methylation marks at the activated promoter	Cancer selective activity of some of the compounds was explored by global gene expression profiling [332]
	▪Uses a two-component reporter-gene system: (1) Trip10 gene promoter positively controls Tet repressor expression, but is silenced by targeted methylation; (2) CMV promoter with two Tet repressor binding sites permits EGFP expression when Tet repressor expression is silenced. ▪ 5 day chemical treatment of human MCF7 cells; ▪ EGFP expression/repression monitored by fluorescence microscopy and high-content image analysis.	▪ 169 compounds with presumptive DNA methyl transferase inhibitory activity (procainamide derivatives) were screened at a single concentration ▪ 46 compounds significantly repressed EGFP expression by reactivating the silenced Tet repressor.	▪ ELISA and Western blotting confirmed suppression of EGFP expression ▪ Demethylation of silenced, endogenous gene, Gstp1, was detected ▪ Global demethylation measured by differential microarray hybridization.	The feasibility of the two-component reporter system for chemical screening was established. This system could be adapted to report on methylation of selected *TSG* promoters [333]
Locus Repression Assay Actively expressing reporter gene promoter	▪ Uses a two-component reporter gene system, similar to the derepression assay, above, except that reporter component 1 is an unmethylated *TSG* promoter (e.g., *HIC1*, or *RASSF1*) which allows expression of the Tet repressor; expressed Tet repressor inhibits EGFP expression of reporter component 2; ▪ Human bone marrow mesenchymal stem cells were transfected and used for targeted promoter methylation experiments	Targeted methylation and silencing of either *HIC1* or *RASSF1* promoters permitted expression of EGFP	▪ Targeted promoter methylation confirmed by methylation-specific PCR ▪ Targeted methylation of *TSG*s induced genome-wide DNA methylation changes ▪ Concurrent targeted methylation of *HIC1* and *RASSF1A* promoters oncogenically transformed MSCs and transformation was reversed by 5-aza-dC.	High throughput chemical screening for *TSG* locus repression may be feasible in this two component system, although none was attempted in this report. This approach may be useful to monitor a range of *TSG* promoter reporter constructs, including the *p16/INK4A* promoter, for silencing among cells during chemical treatments. [151]
MeC phenotyping (3D-qDNA methylation imaging) Genomic DNA in situ	▪ Fixed DU145 prostate cancer and Huh7 hepatocarcinoma cells in 12-well plates; ▪ Processed for anti-5-methylcytosine antibody immunofluorescence; counterstained with DAPI; ▪ Image analysis of nuclear MeC and DAPI intensity distributions and mean intensity signals	Dose-response analysis of two DNA methyltransferase inhibitors was characterized in detail.	MethyLight assay, targeting DNA repetitive elements	The authors suggest that nuclear MeC/DAPI co-distribution is a more robust measure than just MeC intensity [334]
Immunofluorescence detection/high content imaging assays Global histone marks in situ	▪ 384 well plates; fixed cells, human breast epithelial cancer; ▪ Anti H3K27me3 primary antibody; Alexa Fluor secondary antibody; nuclear stain; ▪ High content imaging	▪ Screening of a Biologically Diverse Compound Set (5853 compounds), each at 5 micromolar ▪ 467 H3K27me3 enhancers; 28 H3K27me3 suppressors identified.	“Hits” were confirmed by an 11-point dose response curve and in further biochemical assays with peptide or nucleosome targets for EZH2 methyltransferase activity.	▪ No cellular effects were measured for the compounds that affect H3K27me3 levels; ▪ Many active compounds may have “upstream” pathway activities controlling EZH2 enzyme activity. Other repressive HPTM targets controlling *TSG* silencing and cell transformation (H3K9me2/3) could be assayed in a similar manner [335]
	▪ 384-well plates; fixed cells; ▪ MDA-MB231, MCF7 breast adenocarcinoma and Hela cervical epithelium cell lines ▪ H3K27me3-, H3K27ac-specific primary antibody (and others); fluorescent-conjugated secondary antibody; ▪ Confocal image acquisition	Screening of 9 pyridone EZH2 inhibitors as proof-of-concept.	–	▪ The authors suggest that miniaturized assay (1536 wells) would permit large-scale screening of several million compounds [336]
	▪ Exogenous expression of JMJD3 H3K27me3 demethylase (Table 1, eraser) in human embryonic kidney HEK293 cells; 384-well plates; fixed cells ▪ H3K27me3-specific primary antibodies (and others) and species-specific fluorescent secondary antibodies ▪ Quantitative imaging analysis	Screening of an 87,500 compound library at 10 micromolar; 3524 “hits” inhibiting demethylase activity were identified	“Hits” were confirmed by an 11-point dose response curve, using the same assay and in a biochemical assay for JMJD3 demethylase activity	▪ The single concentration screening design was shown to be reproducible in two independent screenings [337]

## Supplementary Materials

Supplementary materials can be found at www.mdpi.com/1422-0067/18/6/1179/s1, Abbreviations can be found in the Supplementary materials.
