# Supplementary material for: A Tox21 Approach to Altered Epigenetic Landscapes: Assessing Epigenetic Toxicity Pathways Leading to Altered Gene Expression and Oncogenic Transformation In Vitro
Source: Int J Mol Sci. 2017 Jun 1;18(6):1179. doi: 10.3390/ijms18061179 (PMC5486002; doi:10.3390/ijms18061179)
Supplement: Supplementary file 1 [file ijms-18-01179-s001.zip › SUPPLEMENTARY SECTION _Text S2.pdf]

Supplementary Text S2.

Establishment and persistence of oncogenic memory across cell generations

Epigenetic memory requires that histone post-translational modifications (histone marks), or factors that recruit their writers, are passed forward through cell generations. In order to provide stability to a phenotypic memory caused by induced epigenetic lesions, such as oncogenic cellular identities and their underlying gene expression profiles, the altered chromatin architectures need to be continuously re-created during each cell cycle. Chromatin alterations must withstand the extensive nucleosomal reorganizations occurring during each DNA replication fork passage in S-phase and during mitotic chromosome condensation during M phase. Several reviews have been written covering the mechanisms that permit the maintenance of active or silent gene expression states through cell generations, without a change in DNA sequence and in the absence of the original initiating signals [1] [2] [3]. We cover here some of the experimental work that tests the proposed mechanisms for transmission of histone methylation from one cell generation to the next.

The relatively longer half-lives of histone methylation marks may better suit them to the purposes of epigenetic memory than other histone marks [4] and they have more often been the subject of experimental work on their contributions to oncogenic phenotypes in human cells. The current model for self-perpetuating histone marks in human cells (HeLa) is derived from experiments on transmission of the polycomb repressive complex mark, H3K27me3, to the next cell generation [5] . The model explains that: the old histone 3 proteins, with previous methylation marks remaining attached, are distributed evenly onto the two newly replicated DNA strands, while newly synthesized histones are transiently marked by acetylation before transfer to DNA. The acetylation marks are removed from the nascent H3 proteins on DNA and new methylation is re-established, as directed by the old histone modifications that remain in the vicinity of the new histone octamers. The H3K27me3 mark is a binding site for the PRC2 complex, containing the H3K27me3 methylation 'writer', EZH2. Similarly, the H3K9me3 mark is a binding site for heterochromatin protein 1 (HP1), which in turn is a binding partner for the H3K9me3 'writer' SUV39H1.

Among the experiments used to verify this model, luciferase reporter gene constructs were designed that could transiently be bound by the PRC2 complex. The PRC2 complex binding was controlled by tetracycline induced expression of a chimeric protein. The chimera was composed of a specific DNA-binding component (to target the chimera to the upstream promoter region of the reporter gene) and the EED PRC2 component which lead to formation of a trimeric EED/EZH2/SUZ12 complex and formation of H3K27me3 marks at the promoter. Expression from the reporter was repressed for up to four population doublings after washout of the tetracycline inducer in a human embryonic kidney cell line. Genetic experiments varying the functionality of the EZH2 methyltransferase demonstrated that the H3K27me3 mark was necessary for the maintenance of that repression across cell generations.

Another, sophisticated version of gene-targeting technology has been employed in mouse primary cells to assess the persistence of the H3K9me3 mark at an EGFP (enhanced green fluorescent protein) reporter gene, inserted into the first exon of the Oct4 pluripotency factor (a PcG target gene), after removal of the initial targeting stimulus [6]. In this technology, a chimeric small molecule-mediated recruitment (chemically induced proximity; CIP) system which comprises expression of a set of two of chimeric proteins designed to bind different sides of a small molecule, in this case rapamycin. One protein chimera contains specific DNA binding domains that anchor that chimera to the promoter of the reporter construct, while the second chimera contains HP1 which recruits H3K9-specific methyltransferases SUV39H1/2 and SETDB1. Thus, upon addition of the bifunctional small molecule, rapamycin, the anchor is reversibly induced to bind the HP1 partner, which results in tethering the

'writer' methyltransferases to the reporter inserted at the Oct4 locus. This technology was used to demonstrate that the H3K9me3 mark, when increased at the reporter, repressed the EGFP expression, and that the mark was inherited through many cell divisions in a high proportion of the cells, after washout of the initial rapamycin stimulus. The reporter gene also displayed DNA hypermethylation in prolonged presence of the rapamycin inducer and the stochastic re-activation of the EGFP promoter was enhanced by the de-methylating agent 5-aza-2'-deoxycytidine. Greater re-activation occurred in cells that had lower DNA methylation levels, indicating that higher levels of DNA methylation could enhance the permanent heterochromatin silencing of gene expression.

A more recent experimental confirmation of the organized replacement of chromatin components accompanying DNA sysnthesis has been obtained using a technique termed 'nascent chromatin capture' (NCC) [7]. NCC involves short pulse-labelling (20 minutes) of DNA replication forks with a Biotin-dUTP tag, then chromatin crosslinking to the DNA, followed by immunoprecipitation of the biotin-tagged DNA linked to chromatin proteins. This approach permitted the dynamics of histones, their covalent marks and of other chromatin proteins to be measured in G1-S synchronized HeLa cells. The study identified 561 chromatin factors that changed their association with DNA within the first hours post-replication. H3K27me3 and H3K9me3 repressive marks very rapidly re-associated with the nascent DNA. The levels associated with the nascent DNA and levels after 2 hours were similar, supporting the model that these two histone marks are transferred intact with the old histones. In contrast, H4K5ac2, H4K12ac2 histone marks were rapidly lost from the nascent DNA, while H3K9me (monomethylated) continued to accumulate slowly during the chromatin maturation period beyond 2 hours. The PRC2 complex that establishes the H3K27me3 mark was also shown to be associated with both nascent and mature chromatin, consistent with its rapid recruitment by the pre-existing H3K27me3 mark, whereas PRC1 complex was recruited later, during chromatin maturation. In subsequent work it has been shown that for the majority of the histone PTMs, the prereplication level was restored by gradual modification of new histones until they became identical to the old histones, over the period from 2 to 24 h, but that H3K27me3 and H3K9me3 marks were more slowly restored, with continuing addition of these trimethylation marks onto both new and old histones for up to three cell generations after histone incorporation into chromatin [8]. Further, histone PTM levels were shown to be dependent on cell cycle kinetics, suggesting that this mode of PTM maintenance, when affected by cell cycle speed or withdrawal might have impacts on phenotypic plasticity.

The mechanisms for inheritance of the H3K4me3 mark associated with transcriptionally active promoter sites have not yet had detailed studies in rodent or human cells. The existing information on mitotic inheritance was gained from CHIP analysis of several Polycomb/Trithorax -targeted loci in cultured *Drosophila* embryonic cells [9]. When the cells were flow-sorted into early and late DNA replication populations, the abundance of the mark at those loci was observed to have been enriched by a pre-replication boost in early S phase, ahead of its dilution during the completion of DNA replication. This pattern of enrichment resembled that of PcG proteins and the repressive H3K27me3 mark [10]. In staged Drosophila embryos, *in vivo*, the sequence of events appears to differ. Sequential CHIP (re-CHIP) experiments, using first an antibody to proliferating cell nuclear antigen (PCNA), then to the 'Trithorax and PcG methyltransferase 'writers' showed that these proteins were continuously associated with DNA at the replication fork, while the old histones with the activating H3K4me3 or repressive H3K27me3 marks were lost or demethylated in nascent DNA. The unmethylated replacement histones were re-written with the marks, later, during the end of S phase. Proximity ligation and chromatin assembly assays were used to more closely study proximity to the replication fork and temporal recruitment of a larger number of histone-modifying proteins that belong to several different functional groups, including those involved in re-writing H3K4me3 marks [11]. The modifying proteins were shown to be closely associated with PCNA and short nascent DNA fragments , while H3K4me1-3 and other methylation marks were detected only after 1 to 2 hr after replication. It is likely that these proteins may be only

briefly dissociated by the passing replication fork and are held in the vicinity through interactions with PCNA or other replication proteins, then rapidly recruited to nascent DNA. The authors proposed that, in general, histone methylation by itself is unlikely to be an epigenetic mark in this system, and that to re-establish transcriptional states, it is the action of many chromatin proteins acting in concert that drives reconstitution of the chromatin state immediately after DNA replication.

For the H3K4me3 mark, these types of experiments have yet to be reported in rodent or human cells which, unlike *Drosophila,* do not have specific DNA sequence elements for binding TrxG or PcG proteins and may consequently differ in the timing and details of replicating this chromatin mark. The existing data from *Drosophila* leads one to suspect that there are potentially dual mechanisms, in animal cells, based upon the histone methylation writer or the resulting histone mark as the guiding template, so that upon DNA replication, genomic sequences obtain the same activating H3K4me3 modification found in the parental chromatin. Experiments similar to those described above with conditional and gene-targeted modifications of the H3K4me3 mark will be required to gauge the persistence of those modifications in human cells, beyond the period of time in which they were written.

Reference List

1. Sarkies, P.; Sale, J. E. Cellular epigenetic stability and cancer. *Trends in Genetics* **2012**, *28*, 118-127. http://www.scopus.com/inward/record.url?eid=2-s2.0-84857443720&partnerID=40&md5=5abb248231f188400272f77ab5b8d600

2. Budhavarapu, V. N.; Chavez, M.; Tyler, J. K. How is epigenetic information maintained through DNA replication? *Epigenetics and Chromatin* **2013**, *6*, http://www.scopus.com/inward/record.url?eid=2-s2.0-84884759957&partnerID=40&md5=81760387eb4be2874223c4ad347686a2

3. Cheedipudi, S.; Genolet, O.; Dobreva, G. Epigenetic inheritance of cell fates during embryonic development. *Frontiers in Genetics* **2014**, *5*, 19- http://www.scopus.com/inward/record.url?eid=2-s2.0-84897709311&partnerID=40&md5=f1add1ef6327a1f9bd7c55e74868a3c2

4. Barth, T. K.; Imhof, A. Fast signals and slow marks: the dynamics of histone modifications. *Trends in Biochemical Sciences* **2010**, *35*, 618-626. http://www.scopus.com/inward/record.url?eid=2-s2.0-78049246250&partnerID=40&md5=87b61081654268c53f372023c74fc4ea

5. Hansen, K. H.; Bracken, A. P.; Pasini, D.; Dietrich, N.; Gehani, S. S.; Monrad, A.; Rappsilber, J.; Lerdrup, M.; Helin, K. A model for transmission of the H3K27me3 epigenetic mark. *Nature Cell Biology* **2008**, *10*, 1291-1300. http://www.scopus.com/inward/record.url?eid=2-s2.0-55549103314&partnerID=40&md5=920554c4e293d6ff3f4a8c0a4d9f5359

6. Hathaway, Nathaniel-áA; Bell, Oliver; Hodges, Court; Miller, Erik-áL; Neel, Dana-áS; Crabtree, Gerald-áR Dynamics and Memory of Heterochromatin in Living Cells. *Cell* **2012**, *149*, 1447-1460. http://www.sciencedirect.com/science/article/pii/S0092867412006411

7. Alabert, C.; Bukowski-Wills, J. C.; Lee, S. B.; Kustatscher, G.; Nakamura, K.; De Lima Alves, F.; Menard, P.; Mejlvang, J.; Rappsilber, J.; Groth, A. Nascent chromatin capture proteomics determines chromatin dynamics during DNA replication and identifies unknown fork components. *Nature Cell Biology* **2014**, *16*, 281-291. http://www.scopus.com/inward/record.url?eid=2-s2.0-84895455197&partnerID=40&md5=c77a327f5ec7f96d333111a9fd87be02

8. Alabert, C.; Barth, T. K.; Rever+¦n-G+¦mez, N.; Sidoli, S.; Schmidt, A.; Jensen, O.; Imhof, A.; Groth, A. Two distinct modes for propagation of histone PTMs across the cell cycle. *Genes Dev.* **2015**, *29*, 585-590. https://www.scopus.com/inward/record.uri?eid=2-s2.0-84925238653&partnerID=40&md5=08fe59b8519720046dadd32c23ee6bf5

9. Petruk, S.; Sedkov, Y.; Johnston, D. M.; Hodgson, J. W.; Black, K. L.; Kovermann, S. K.; Beck, S.; Canaani, E.; Brock, H. W.; Mazo, A. TrxG and PcG proteins but not methylated histones remain associated with DNA through replication. **2012**, *150*, 922-933. https://www.scopus.com/inward/record.uri?eid=2-s2.0-84865681429&partnerID=40&md5=a0c1b5e25478303dedb2e167d140e94e

10. Lanzuolo, Chiara; Lo Sardo, Federica; Orlando, Valerio Concerted epigenetic signatures inheritance at PcG targets through replication. *Cell Cycle* **2012**, *11*, 1296-1300. http://dx.doi.org/10.4161/cc.19710

11. Petruk, S.; Black, K. L.; Kovermann, S. K.; Brock, H. W.; Mazo, A. Stepwise histone modifications are mediated by multiple enzymes that rapidly associate with nascent DNA during replication. **2013**, *4*, https://www.scopus.com/inward/record.uri?eid=2-s2.0-84906725999&partnerID=40&md5=80004fdcb57308affa9176529aaa7baa
