# Supplementary material for: A Tox21 Approach to Altered Epigenetic Landscapes: Assessing Epigenetic Toxicity Pathways Leading to Altered Gene Expression and Oncogenic Transformation In Vitro
Source: Int J Mol Sci. 2017 Jun 1;18(6):1179. doi: 10.3390/ijms18061179 (PMC5486002; doi:10.3390/ijms18061179)
Supplement: Supplementary file 1 [file ijms-18-01179-s001.zip › List of abbreviations.pdf]

List of abbreviations

Name	Description
53BP1	Tumor protein p53 binding protein 1
2-HG	2-hydroxyglutarate
3-MCA	3-methylcholanthrene
5-Aza-dC	5-aza-2'-deoxycytidine
5caC	5-Carboxylcytosine
5fC	5-Formylcytosine
5hmC	5-Hydroxymethylcytosine
5mC	5-methylcytosine
ac	acetylation
ADD	ATRX-Dnmt3-Dnmt3L
ADP	Adenosine diphosphate
AKT	Serine/threonine protein kinase. Originally, the letter "A" and "K" were to identify stocks of mice wheras "T" was to identify a transforming retrovirus.
AML	Acute myeloid leukemia
ANRIL	Antisense non-coding RNA in the INK4 locus
AP-1	Activator protein 1
Apc	Adenomatous polyposis coli
ARF	ADP-ribosylation factor
Arg	Arginine
ASH1/2	Absent, small, or homeotic discs 1/2
ASH1L	ASH1 like histone lysine methyltransferase
Ash2L	ASH2 like histone lysine methyltransferase
ATM	Ataxia telangiectasia gene mutated serine/threonine kinase
ATP	Adenosine triphosphate
ATR	Ataxia telangiectasia and Rad3 related serine/threonine kinase
ATRX	Alpha-thalassemia and mental retardation X-linked
Aurora B	Aurora kinase B
AXIN2	Axin 2
BAF	B-associated fitness
BAZ2 A/B	Bromodomain adjacent to zinc finger 2A/2B
BER	Base excision repair
BMI-1	BMI1 proto-oncogene, polycomb ring finger
BMP	Bone morphogenetic protein
BPA	Bisphenol A
BPDE	Benzo[a]pyrene diol epoxide
B-RAF	B-Raf proto-oncogene, serine/threonine kinase
BRCA1	Breast cancer 1, DNA repair associated

BRCT	BRCA1 C Terminus
BRG1	Brahma-related gene-1
CAF1	Chromatin assembly factor 1
CBP	CREB binding protein
CBX	Chromodomain-containing CBX family proteins
CD44	CD refers to cluster of differentiation, also known as cluster of designation or classification determinant. The numbering system is used for the identification and investigation of different cell surface molecules.
Cdh1	Cadherin 1
CDK	Cyclin-dependent kinase
CDKN	Cyclin dependent kinase inhibitor
CHD	Chromodomain helicase DNA binding protein
ChIP	Chromatin immunoprecipitation
CIP1	Cyclin-dependent kinase inhibitor
CMV	Cytomegalovirus
c-myc	MYC proto-oncogene
CoREST	Repressor element-1 silencing transcription factor corepressor 1
CpG	5'—Cytosine—phosphate—Guanine—3'
CRABP2	Cellular Retinoic Acid Binding Protein 2
CRBP1	Cellular retinol binding protein 1
CREB	cAMP response element-binding protein
CTCF	CCCTC-binding factor
CTIP2	COUP-TF-interacting protein 2
CUL4	Cullin 4
CxxC5/1	CXXC finger protein 5/1
CYP26A1	Cytochrome P450 family 26 subfamily A member 1
DAPI	4',6-diamidino-2-phenylindole
DDB1	DNA damage-binding protein 1 i
DES	Desmin
dG	Deoxyguanosine
DKK1-4	Dickkopf WNT signaling pathway inhibitors 1-4
Dlx-1-6	Distal-less homeobox proteins 1-6
DMRs	Differentially methylated regions
DMSO	Dimethyl sulfoxide
DNA	Deoxyribonucleic acid
DNMT	DNA (cytosine-5)-methyltransferase
DUSP1-5	Dual specificity phosphatases 1-5
E2F	E2F transcription factor
E3	Ubiquitin-protein ligase

EED	Embryonic ectoderm development
EGFP	Enhanced Green Fluorescent Protein
EGFR	Epidermal growth factor receptor
EHMT1/2	Euchromatic histone lysine methyltransferase 1/2
ELISA	Enzyme-linked immunosorbent assay
EMT	Epithelial-to-mesenchymal transition
ENCODE	Encyclopedia of DNA Elements
Engrailed	Engrailed homeobox 2
Ep-CAM	Epithelial cell adhesion molecule
EphB2	EPH receptor B2
ERK	Extracellular regulated MAP kinase
esBAF	Embryonic stem cell BRG1 ATPase associated factor
ESC	Embryonic stem cells
ESET	Erg-associated protein with SET domain
EZH1-2	Enhancer of zeste 1 polycomb repressive complex 2 subunit
FA	Folic acid
FAD	Flavin adenine dinucleotide
FGF	Fibroblast growth factor
G1	Cell cycle Gap 1 phase
G9a	Histone methyltransferase
GAD1	Glutamic acid decarboxylase 1
GADD34	Growth arrest and DNA damage 34
GFP	Green fluorescent protein
GLP	Glucagon-like peptide-1
Gstp1	Glutathione S-transferase pi 1
H1	Histone protein 1
H19	Long non-coding RNA
H2A	Histone protein 2A
H2A.Z	H2A histone family member Z
H2AX	Histone protein 2AX
H2B	Histone protein 2B
H3	Histone protein 3
H3S10p	Histone H3 serine 10 phosphorylation
H4	Histone protein 4
Ha-ras	Harvey Ras proto-oncogene, GTPase
HCC	Hepatocellular carcinoma
HDAC	Histone deacetylases
HDMs	Histone demethylases
HIC1	Hypermethylated in cancer 1

HMEC	Human Mammary Epithelial Cells
HMGA2	High mobility group at-hook 2
HMT	Histone methyltransferase
HOTAIR	HOX transcript antisense RNA
HOX	Homeobox
HP1	Heterochromatin protein 1
HpaII	Restriction endonuclease, Haemophilus parainfluenzae
HPC1 - 3	Human polycomb 1-3
HPH1 - 3	Polyhomeotic homolog 1-3
HPTM	Histone post-translational modifications
HPV E7	Human papilloma virus E7 oncoprotein
hSETD1A/1B	Human SET domain containing 1A/1B
hSNF5	Human Sucrose Non-Fermentable 5
hTERT	Human telomerase reverse transcriptase
ICF	Immunodeficiency, centromeric region instability, and facial anomalies
IDH 1-2	Isocitrate dehydrogenases 1-2
IGF	Insulin like growth factor
ING	Inhibitor of growth
INK4a	Inhibitor of cyclin-dependent kinase 4a
INK4b	Inhibitor of cyclin-dependent kinase 4b
INO81	Inositol requiring 80
iPSC	Induced pluripotent stem cell
ISWI	Imitation Switch, Chromatin Remodeling
Jak2	Janus kinase 2
JARID1a/1b	Jumonji AT-rich interactive domain 1a/1b
Jhdm1b	JmjC domain-containing histone demethylase 1b
JMJD	Jumonji domain containing protein
K	Lysine
KAT	Lysine acetyl transferases
KDM	Lysine demethylases
Kip2	Kinesin-like protein 2
KISS1	Kisspeptin-1, Metastasis-Suppressor
KMT	lysine methyltransferases
KO	knockout
LEF	Lymphoid enhancer binding factor
Let-7c	MicroRNA let-7c
LHX	LIM/homeobox protein
lincRNAs	Long intergenic non-coding RNAs
lipocalin-2	LCN2

LSD1	Lysine-specific histone demethylase 1
MAL	mal, T-cell differentiation protein
MAP	Mitogen-activated protein kinase
MBD	methyl-CpG- binding domain
MBLR	MEL18- and BMI1-like ring finger protein
MCAF1	MBD1-containing chromatin-associated factor 1
mCpA	Methylated 5'—C—phosphate—A—3'
mCpC	Methylated 5'—C—phosphate—C—3'
mCpT	Methylated 5'—C—phosphate—T—3'
MDM2	Mouse double minute 2
me1	monomethylation
me2	dimethylation
me3	trimethylation
MeC/DAPI	5-methylcytosine(Mec) /4,6-diamidino-2-phenylindole (DAPI)
MeCP2	Methyl CpG binding protein 2
MEFs	Mouse Embryonic Fibroblasts
MEK	Mitogen-activated protein kinase kinases (also known as MAP2K, MAPKK)
MEL18	Polycomb Group RING finger Protein
MGMT	O-6-Methylguanine-DNA Methyltransferase
Mi-2	Chromodomain-helicase-DNA-binding protein
miR	MicroRNA
MIR31HG	MicroRNA-31 host gene
MLH1	MutL homolog 1, colon cancer, nonpolyposis type 2 (E. coli)
MLL 1-4	Acute lymphoblastic leukemia 1-4, aliases for KMT2A Gene
MMA	Methyl Methacrylate
MMP7	Matrix metalloproteinase-7
MMTV	Mouse mammary tumor virus
MNU	N-Methyl-N-Nitrosourea
MOZ	Monocytic leukemia zinc-finger protein
MPP8	M-phase phosphor protein 8
MSH 2-6	DNA mismatch repair protein Msh2-6
MYSM1	myb-like, SWIRM and MPN domains 1
MYST3	MYST histone acetyltransferase (monocytic leukemia) 3
NAD	Nicotinamide-adenine dinucleotide
NANOG	Nanog homeobox Gene
NCATS	The National Center for Advancing Translational Sciences
NDR	Nucleosome depleted region
Next Gen Seq	Next-generation sequencing

NoRC	Nucleolar remodeling complex
NOTCH	Notch (DSL) proteins
NSPC1	Polycomb group RING finger protein 1
NURD	Nucleosome remodeling deacetylase
OCT4	octamer-binding transcription factor 4
OGG1	8-oxoguanine DNA glycosylase
OHdG	8-hydroxy-2' -deoxyguanosine
OIS	Oncogene induced senescence
OLIGO2	Oligodendrocyte transcription factor
p107	107 kDa retinoblastoma-associated protein
p120-catenin	Prototypic member of a subfamily of Armadillo-domain proteins
p130	130 kDa retinoblastoma-associated protein
p14	Tumour suppressor protein also called ARF or $p14^{ARF}$. It is a 14 kDa protein in human encoded from the INK4a/ARF or CDKN2A locus.
p15/INK4B	Cyclin-dependent kinase inhibitor 2B (CDKN2B or p15, $p15^{INK4b}$, and inhibits CDK4)
p16	Cyclin-dependent kinase inhibitor 2A (or p16INK4a, a 16 kDa tumour suppressor protein encoded from the INK4a/ARF or CDKN2A locus). It inhibits CDK4 and CDK6.
p21	Cyclin-dependent kinase inhibitor 1 from the CDKN1A gene. A 21 kDa protein associated primarily with inhibition of CDK2. Also known as $p21^{Cip1}$, or $p21^{Waf1}$.
p300	Histone acetyltransferase p300
p53	Tumor Protein P53
p53BP2	Tumour suppressor p53-binding protein 2
p57/Kip2	Cyclin-dependent kinase inhibitor belonging to the CIP/KIP family
PARG	Poly(ADP-ribose) glycohydrolase
PARP1/2	Poly [ADP-ribose] polymerase 1/2
PAX	Paired box
PcG	Polycomb group
PCNA	Proliferating cell nuclear antigen
PCR	Polymerase chain reaction
PDGFRA	Platelet-derived growth factor receptor A
PGC	Primordial germ cells
PHD	Plant homeodomain
PI3K	Phosphatidylinositol-4,5-bisphosphate 3-kinase
PKC	Protein kinase C
PoT	Pathway of Toxicity
PPP1R15A	Protein Phosphatase 1 Regulatory Subunit 15A

pRB	Retinoblastoma protein
pRB-P	Phosphorylated retinoblastoma protein
PRC1-4	Polycomb repressive complexes 1- 4
PRDM2	PR domain zinc finger protein 2
PRMT	Protein Arginine methyltransferases
PTEN	Phosphatase and tensin homolog
PTM	Post translational modification
PWWP	PWWP domain (Pro-Trp-Trp-Pro residues)
qHTS	Quantitative high-throughput screening
qRT-PCR	Quantitative Reverse Transcription PCR
R	Arginine
RAF	Serine/threonine kinase; Rapidly Accelerated Fibrosarcoma (can be a-, b-c-, or v-RAF)
RAR□2	Retinoic acid receptor beta 2
RARα	Retinoic acid receptor alpha
RAS	proto-oncogene, member of the Ras superfamily of proteins, GTPase
RASL – Seq	RNA-mediated oligonucleotide Annealing, Selection, and Ligation with Next-Gen sequencing
RassF1	RAS association domain family 1
Rb	Retinoblastoma
RBBP4-5	Retinoblastoma binding protein 4-5
rDNA	Ribosomal DNA
REST	Repressor element-1 silencing transcription factor
Rfx5	Regulatory factor X5
Rfxank	Regulatory factor X associated ankyrin containing protein
Rfxap	Regulatory factor X associated protein
Ring1A	Ring finger protein 1A
RNA	Ribonucleic acid
RNF2	Ring finger protein 2
ROS	Reactive oxygen species
S	Serine
SAM	S-adenosyl-L-methionine
SCML1-2	Sex comb on midleg zinc-finger protein 1-2
SET1	Su (var) 3-9, Enhancer-of-zeste and Trithorax1
SETD1A/1B	Su (var) 3-9, Enhancer-of-zeste and Trithorax domain 1A/1B
SETDB 1-2	Su (Var) 3-9, enhancer of Zeste, Thritorax domain, bifurcated 1-2
SFRP5	Secreted frizzled related protein 5
SHE	Syrian hamster embryo cells
Shh	Sonic hedgehog
shRNA	Short hairpin RNA

SIPS	Stress-induced premature senescence
siRNA	Short interfering RNA
SIRT1	Sirtuin 1
SIS	Stress-induced senescence
SIX	SIX homeobox
SMARCAD1	SWI/SNF-related, matrix-associated actin-dependent regulator of chromatin, subfamily a, containing DEAD/H box 1
Sncg	Synuclein-gamma
SNF5	Subunit of the SWI/SNF chromatin remodeling complex
SOX2	SRY-Box 2
S-phase	Cell cycle synthesis phase
SPRY2/4	Sprouty 2/4
SRA	Set and RING finger-associated
SRY	Sex Determining Region Y
ssDNA	single-stranded DNA
SUV39H1/H2	Suppressor of variegation 39 homolog family 1/2
SUZ12	Suppressor of zeste-12
SWI	SWItching deficient
SWR1	ATP-dependent chromatin-remodeling complex
T	Threonine
TCDD	2,3,7,8-Tetrachlorodibenzo-p-dioxin
TCF	T cell-specific transcription factor
TET	Ten Eleven Translocation enzyme
TG:meCG	Thymine-guanine:methylated cytosine-guanine
TGF	transforming growth factor
TIP5	Tonoplast intrinsic protein 5
Tox21	Toxicology in the 21st Century
TP53	Tumor Protein P53
TrxG	Trithorax group
TSG	Tumour suppressor genes
TSS	Transcription start site
TT21C	Toxicology Testing in the 21st Century
TUCP	Transcripts of uncertain coding potential
Twist	Reposition of chromatin remodeling complexes
ub	Ubiquitinylated
UG:meCG	Uracil-guanine:methylated cytosine-guanine
UHRF	Ubiquitin-like, containing plant homeodomain (PHD) and RING finger domain
UIM	Ubiquitin interacting motif
UTX	Ubiquitously transcribed tetratricopeptide repeat, X chromosome

vHMEC	Variant human mammary epithelial cells
v-myc	Myelocytomatosis viral oncogene
WAF1	Wildtype p53-activated fragment 1 (Cyclin-dependent kinase inhibitor)
WDR5	WD (trp-asp) repeat domain 5
WH	Winged-helix
Wnt	Wingless-type MMTV integration site family
Wnt5a	Wnt family member 5A
ZBTB	Zinc finger and BTB domain (bric-à-brac, tramtrack and broad complex)
ZF-CW	Zinc finger motif, cysteine and tryptophan residues
ZFP57	Zinc finger protein 57
α-kg	α-ketoglutarate
