# Supplementary material for: A Tox21 Approach to Altered Epigenetic Landscapes: Assessing Epigenetic Toxicity Pathways Leading to Altered Gene Expression and Oncogenic Transformation In Vitro
Source: Int J Mol Sci. 2017 Jun 1;18(6):1179. doi: 10.3390/ijms18061179 (PMC5486002; doi:10.3390/ijms18061179)
Supplement: Supplementary file 1 [file ijms-18-01179-s001.zip › SUPPLEMENTARY SECTION _Table S1.pdf]

Approaches to determining the mechanistic connections between toxicity pathway perturbations and adverse phenotypes.

In the Tox21 testing paradigm, emphasis is given to measuring phenotypic endpoints in human cell cultures, rather than using cellular extracts or purified molecular components, in order to have the most species-relevant and structurally realistic evaluation of pathway perturbations that would lead to adverse effects in critical cellular systems and behaviour (chemical interference). Ascertaining mechanistic validity and establishing causality in molecular toxicity pathways are two fundamental issues that must be addressed, to provide high-throughput assays that could most effectively inform decision-making in regulatory settings. Criteria for assessing published experimental studies that have investigated causality in epigenetic pathways leading to oncogenic transformation in human cells are summarised here. To aid in determining causality for any step in a molecular pathway leading to toxicity, Hartung *et al.* have succinctly re-stated the experimental steps that would affirm Kock's postulates for experimentally showing that an infectious organism causes a disease as "show it, block it, and induce it" [1]. Accordingly, within epigenetic pathways that involve effects on transcriptional activity:

> "Showing it" could involve using separate methodologies (eg, various measures of RNA abundance; measurements of association between specific DNA segments and components of epigenetic multi-protein complexes) to measure closely-aligned aspects of the molecular event. Performing replicate experiments, and demonstrating concentration-response relationships that parallel those for an adverse cellular event would further solidify a suspected association of a molecular event with the adverse cellular outcome (essentially fulfilling the Bradford Hill criteria for assessing evidence of causation: the association must demonstrate strength; consistency; specificity; temporality; biological gradient; plausibility; and responsiveness to experimental conditions [2]).
>
> "Blocking it" would require using specific chemical antagonists (biological or xenobiotic) or molecular biological tools such as gene knockouts, gene knockdown (RNAi, shRNA) or in some cases, antibodies.
>
> "Inducing it" might employ molecular tools to create controlled induction or repression of the expression among the upstream components of a molecular pathway in the absence of the specific toxicant(s), in order to gage the effect(s) on the downstream pathway steps and, ultimately, the adverse cellular outcomes.

The contributions of the latter two types of experiments in establishing causality between epigenetic pathway perturbations and human cell oncogenic transformation processes are emphasized throughout the sections of this review. Similar considerations of the experimental evidence for causality have been applied in assessing the probable contributions to adverse phenotypes from gene expression changes, in order to strengthen the interpretations of in vivo toxicogenomic results [3]. It is to be expected that high throughput assay designs based upon mechanistic knowledge, rather than correlative associations, would be less likely to falsely

identify risks that might arise when changes in epigenetic controls and subsequent gene expression changes are only coincidental or inconsequential molecular responses, without causal connection to adverse cellular phenotypes. Table S1 presents a comparison between Koch's postulates for proof of causality by infectious disease agents and several analogous postulates re-written for an epigenetic mediator in a molecular pathway of toxicity related to oncogenic cell transformation *in vitro*. These guidelines could be followed to interrogate an established correlation in order to establish confidence that mechanistic relationships exist across the sequence of exposures, epigenetic alterations and adverse outcomes in an *in vitro* setting.

Table S1 Alignment of Kock's postulates for experiments establishing causality in infectious disease and plausible experimental approaches that could establish causal molecular steps in an epigenetic pathway of toxicity leading to adverse phenotypic changes in human cells.

Koch's postulates for experimental proof of causality for microbial pathogens in disease	Experimental evidence for establishing causality in epigenetic pathways of toxicity (PoT):<br>Exemplified by the involvement of epigenetic alterations acting on tumor suppressor gene expression as a causal pathway leading to oncogenic cell transformation.
Establish the correlation:<br>The organism should always be found in animals suffering from the disease and should not be present in healthy individuals	Chemically-induced changes in an epigenetic mediator (DNA methylation; histone modification) should be consistently be correlated with specific phenotypic measured as cell transformation.
Isolate/characterize the agent:<br>The organism must be cultivated in pure culture away from the animal body	Key alterations in the putative epigenetic pathway of toxicity should be measured along with the subsequent changes in RNA/protein expression. Molecular changes induced in the epigenetic pathway should be confirmed with alternate methods (eg., RT-PCR or RNAse protection for mRNA and non-coding RNAs; Antibody-based immunoprecipitation for global histone modifications; immunoprecip/PCR enrichment for specific gene promoters; bisulfite converted pyrosequencing, MSP, melt-curve, etc for DNA methylation).
Experimentally cause the disease:<br>A pure culture, when inoculated into susceptible animals, should initiate the characteristic disease symptoms	Experimental perturbation of the epigenetic pathway components by malipulative, molecular tools (eg RNAi knockdown or; targeted DNA methylation, engineered overexpression) or chemical means (enhancing or blocking writers/erasers/editors/readers) should induce or inhibit the transformed cell phenotype or its characteristic cellular/biochemical activities (altered cell cycle phases, invasive properties, etc).
Confirm that the agent is present in the experimental disease.<br>The organism should be re-isolated from these experimental animals and cultured again in the laboratory, after which it should still be the same as the original organism	Cell-transforming, experimental perturbation of epigenetic pathway components by malipulative, molecular tools should induce the previously correlated changes in specific target RNAs, proteins and biochemical activities. Graded changes made experimentally to epigenetic pathway components (writers/erasers/editors/readers) should generate downstream mRNA, protein and phenotypic changes that are comparable in magnitude to the changes induced by chemical/physical toxins in the experimentally exposed cells.

Reference List

1. Hartung, T.; Hoffmann, S.; Stephens, M. Food for thought Mechanistic validation. *Altex* **2013**, *30*, 119-130. http://www.scopus.com/inward/record.url?eid=2-s2.0-84877615913&partnerID=40&md5=a00fbbdc2885c952108f7a9282a34e05

2. HILL, A. B. THE ENVIRONMENT AND DISEASE: ASSOCIATION OR CAUSATION? *Proceedings of the Royal Society of Medicine* **1965**, *58*, 295-300. http://www.scopus.com/inward/record.url?eid=2-s2.0-31844443695&partnerID=40&md5=81516fcd6172f3952f312b427ce4da78

3. Zheng, J. L.; Parfett, C.; Williams, A.; Yagminas, A.; Zhou, G.; Douglas, G. R.; Yauk, C. L. Assessment of subclinical, toxicant-induced hepatic gene expression profiles after low-dose, short-term exposures in mice. *Regulatory Toxicology and Pharmacology* **2011**, *60*, 54-72. http://www.scopus.com/inward/record.url?eid=2-s2.0-79955987896&partnerID=40&md5=a3e7abb8880a3448288fd13cc2a16ddd
