# Supplementary material for: A Tox21 Approach to Altered Epigenetic Landscapes: Assessing Epigenetic Toxicity Pathways Leading to Altered Gene Expression and Oncogenic Transformation In Vitro
Source: Int J Mol Sci. 2017 Jun 1;18(6):1179. doi: 10.3390/ijms18061179 (PMC5486002; doi:10.3390/ijms18061179)
Supplement: Supplementary file 1 [file ijms-18-01179-s001.zip › SUPPLEMENTARY SECTION _Text S1.pdf]

Supplementary Text S1

The epigenetic landscape metaphor and some recent applications related to human cell oncogenic transformation.

As a starting point in examining how epigenetic pathways of toxicity might be developed as molecular targets for toxicity testing *in vitro*, it is instructive to briefly re-cap the formative beginnings of epigenetic science. The term "epigenetic landscape," originated as an organizing concept formulated over 60 years ago by the developmental biologist, Conrad Waddington [1]. The landscape metaphor was employed to illustrate how a diploid genome might naturally give rise to and eventually restrict cells to an assortment of specific phenotypes during embryonic development - from the beginnings in a single, undifferentiated embryonic progenitor cell, through to the final forms and functions of the various specifically differentiated cell phenotypes within the specialized tissues of a multicellular organism (reviewed by Slack [2,2]). In his forward-thinking exercise, developed primarily before the biochemical characterization of DNA, Waddington envisaged a 3D model for cellular differentiation. It presented developmental choices as a topographic 'landscape' (hills, valleys, canyons, channels) constraining the routes, or channels, through which an undeveloped, pluripotent cell could be directed to navigate while descending through the 'landscape' toward a more differentiated cell species (with lesser developmental potential) (Figure 2). The distinct channels formed within the landscape were hypothesized to be formed above a multidimensional substructure linked to a set of developmental genes that control cellular metabolic processes and form an interconnected framework of information, or 'forces', that are the determinants of the developmental channels. More recently, mathematical methods have been employed to compute quasi-potential landscapes for gene regulatory network dynamics, where stable steady states (attractors) are separated by transitional barriers that need to be overcome to permit state changes from any one attractor to another. The attractors and transitional barriers correspond to the valleys (leading to cell fates) and hills (preventing trans-differentiation or reprogramming of cell identity) portrayed to be the result of forces controlled by the developmental genes in the qualitative Waddington model [3,4]. The landscape metaphor continues to be invoked to interpret alterations in the epigenetic control of gene expression for their effects on cellular phenotype or identity. Examples include: effects related to reprogramming transcription factors used in engineered trans-differentiation of cells [5] or the observed epigenetic disturbances in tumour cells [6]. One view has been presented that proposes an environmentally-induced flattening of the epigenetic landscape in tumour cells (eg by carcinogens or diet, as well as injury and inflammation). The lowering of cellular developmental barriers provides more flexible possibilities for cells to express alternate gene expression signatures, thereby making more paths available to phenotypes that are advantageous to cancer cell survival and growth [7]. Similarly, multi-point perturbations within gene regulatory network dynamics, or in network landscape topography, have been hypothesised to be partly caused by instability in the epigenome, due to chemotherapeutic-induced stress which results in further selectable phenotypic changes in cancer cells [8].

Reference List

1. Waddington, C. H. The epigenotype. 1942. *International journal of epidemiology* **2012**, *41*, 10-13. http://www.scopus.com/inward/record.url?eid=2-s2.0-84863773570&partnerID=40&md5=46da428e2d6dbfc8453d157aba2f605f

2. Slack, J. M. W. Conrad Hal Waddington: The last Renaissance biologist? *Nature Reviews Genetics* **2002**, *3*, 889-895. http://www.scopus.com/inward/record.url?eid=2-s2.0-0036845254&partnerID=40&md5=4c2e844a8cb2c2a570dd06753eb6c249

3. Zhou, Joseph Xu; Aliyu, M. D. S.; Aurell, Erik; Huang, Sui Quasi-potential landscape in complex multi-stable systems. *Journal of The Royal Society Interface* **2012**, *9*, 3539-3553. http://rsif.royalsocietypublishing.org/content/9/77/3539.abstract

4. Bhattacharya, S.; Zhang, Q.; Andersen, M. E. A deterministic map of Waddington's epigenetic landscape for cell fate specification. *BMC Syst. Biol.* **2011**, *5*, 85- http://www.scopus.com/inward/record.url?eid=2-s2.0-79957461243&partnerID=40&md5=324ff4226be68a4e5e4295e289158338

5. Davis, F. P.; Eddy, S. R. Transcription Factors That Convert Adult Cell Identity Are Differentially Polycomb Repressed. *PLoS ONE* **2013**, *8*, e63407- http://www.scopus.com/inward/record.url?eid=2-s2.0-84877032446&partnerID=40&md5=5fc1e3f0cef5ff0e6f2971b30641d8bd

6. Baylin, S. B. The cancer epigenome: Its origins, contributions to tumorigenesis, and translational implications. *Proceedings of the American Thoracic Society* **2012**, *9*, 64-65. http://www.scopus.com/inward/record.url?eid=2-s2.0-84862622426&partnerID=40&md5=65b04ae716d0fe0b706b14bdfa7c3f44

7. Timp, W.; Feinberg, A. P. Cancer as a dysregulated epigenome allowing cellular growth advantage at the expense of the host. *Nature Reviews Cancer* **2013**, *13*, 497-510. http://www.scopus.com/inward/record.url?eid=2-s2.0-84881086323&partnerID=40&md5=0d5129b5ab5ed3d22dc3f1c049e11130

8. Huang, S. Genetic and non-genetic instability in tumor progression: Link between the fitness landscape and the epigenetic landscape of cancer cells. *Cancer Metastasis Rev.* **2013**, *32*, 423-448. http://www.scopus.com/inward/record.url?eid=2-s2.0-84890313470&partnerID=40&md5=682fc5d253298a027058450f478f2bb0
